# Single-nucleus multi-omic profiling of human placental syncytiotrophoblasts identifies cellular trajectories during pregnancy

**DOI:** 10.1038/s41588-023-01647-w

**Published:** 2024-01-24

**Authors:** Meijiao Wang, Yawei Liu, Run Sun, Fenting Liu, Jiaqian Li, Long Yan, Jixiang Zhang, Xinwei Xie, Dongxu Li, Yiming Wang, Shiwen Li, Xili Zhu, Rong Li, Falong Lu, Zhenyu Xiao, Hongmei Wang

**Affiliations:** 1 The Key Laboratory of Organ Regeneration and Reconstruction, State Key Laboratory of Stem Cell and Reproductive Biology, Institute of Zoology, Chinese Academy of Sciences, Beijing, China; 2 Beijing Institute for Stem Cell and Regenerative Medicine, Beijing, China; 3 State Key Laboratory of Molecular Developmental Biology, Institute of Genetics and Developmental Biology, Chinese Academy of Sciences, Beijing, China; 4 Dushu Lake Hospital Affiliated to Soochow University, Suzhou, China; 5 Medical Center of Soochow University, Suzhou, China; 6 Suzhou Dushu Lake Hospital, Suzhou, China; 7 University of Chinese Academy of Sciences, Beijing, China; 8 Center for Reproductive Medicine, Department of Obstetrics and Gynecology, Peking University Third Hospital, Beijing, China; 9 National Clinical Research Center for Obstetrics and Gynecology, Peking University Third Hospital, Beijing, China; 10 Key Laboratory of Assisted Reproduction (Peking University), Ministry of Education, Beijing, China; 11 Beijing Key Laboratory of Reproductive Endocrinology and Assisted Reproductive Technology, Peking University Third Hospital, Beijing, China; 12 School of Life Science, Beijing Institute of Technology, Beijing, China

**Keywords:** Developmental biology, Data mining

## Abstract

The human placenta has a vital role in ensuring a successful pregnancy. Despite the growing body of knowledge about its cellular compositions and functions, there has been limited research on the heterogeneity of the billions of nuclei within the syncytiotrophoblast (STB), a multinucleated entity primarily responsible for placental function. Here we conducted integrated single-nucleus RNA sequencing and single-nucleus ATAC sequencing analyses of human placentas from early and late pregnancy. Our findings demonstrate the dynamic heterogeneity and developmental trajectories of STB nuclei and their correspondence with human trophoblast stem cell (hTSC)-derived STB. Furthermore, we identified transcription factors associated with diverse STB nuclear lineages through their gene regulatory networks and experimentally confirmed their function in hTSC and trophoblast organoid-derived STBs. Together, our data provide insights into the heterogeneity of human STB and represent a valuable resource for interpreting associated pregnancy complications.

## Abstract (web summary)

Multi-omic analysis of single nuclei from 12 human placentas collected during early-stage and late-stage pregnancy characterizes syncytiotrophoblast diversity. Gene regulatory network analysis implicates candidate lineage regulators such as STAT5A and CEBPB.

## Main

The placenta, which is an integral part of the maternal–fetal interface, serves the critical function of supplying nutrition and oxygen to the developing fetus and removes waste materials and carbon dioxide. It is a transient organ that responds to developmental cues and environmental stimuli during pregnancy^1–3^. The human placenta develops during implantation when trophoblasts differentiate from the trophectoderm (TE) of the blastocyst^4,5^. Following implantation, the TE gives rise to the following three critical trophoblast cell types: cytotrophoblast cells (CTBs), extravillous trophoblast cells (EVTs) and multinucleated syncytiotrophoblast (STB). CTBs can act as progenitor cells that differentiate into EVTs or fuse to the multinucleated STB^6^. The STB is in direct contact with the maternal tissue and blood, carrying out various functions such as hormone synthesis, immunological defense and active transport during pregnancy^5^, and thus has an essential role in maintaining a successful pregnancy^7–9^. Insufficient placental development can lead to conditions such as preeclampsia and intrauterine growth restriction^10–13^.

The development of the multinucleated STB involves complex structural arrangements and functionalization^14^. The process of multinucleation in the STB is crucial for protecting the placenta against pathogens. The absence of multinucleated STB increases susceptibility to infections at the maternal–fetal barrier. As pregnancy progresses, aged nuclei known as syncytial knots are gradually replaced by the CTB nuclei underneath^15–18^. At term, the number of STB nuclei reaches ~58 billion^19,20^. Previous histological studies suggested the existence of subpopulations of nuclei within the STB^21–24^. However, whether the STB nuclei exhibit development-dependent transcriptional and functional dynamics remains under-appreciated.

Our knowledge of the cell-type-specific transcriptional landscape in the human placenta has been reinterpreted in light of single-cell RNA-sequencing (scRNA-seq) data^25–31^. Past scRNA-seq studies have described the transcriptional landscape of the placenta, such as nonproliferative EVTs^27^. However, scRNA-seq has not been applied to STB due to its multinucleated nature, and furthermore, isolating single STB nuclear is difficult, leaving fundamental questions about the heterogeneity of the STB nuclei, gene expression and regulators largely unanswered^32^. The utilization of single-nucleus RNA sequence (snRNA-seq) has transformed our ability to identify various nuclear types, states and genetic diversity within the shared cytoplasm of tissues like muscle, as well as other hard-to-dissociated samples like brain. More recently, snRNA-seq has been used to investigate placental samples during early pregnancy^33^.

Here we constructed a comprehensive map of nuclear identities in the human STB during early and late pregnancy by integrating snRNA-seq and single-nucleus ATAC sequencing (snATAC-seq) on 101,543 placental nuclei. Our study reveals the dynamic heterogeneity of the STB nuclei, their differentiation routes and their potential roles during early and late pregnancy. We identified master transcription factors (TFs) that are biased toward diverse STB nuclear lineages, including MITF, STAT5A, CEBPB and FOSL2. We described their key gene regulatory networks at different stages of pregnancy. We confirmed the presence of STB nuclear subtypes in human trophoblast stem cell (hTSC)-derived STB. Our results indicated that the critical functions of master TFs were partially retained in in vitro models of STB differentiated from hTSCs and trophoblast organoids. Taken together, our work provides a comprehensive analysis of STB nuclear heterogeneity, highlights new regulatory mechanisms and represents a valuable resource for interpreting associated pregnancy complications.

## Results

### snRNA-seq and snATAC-seq profiling of human placentas revealed dynamic features and heterogeneity of STB nuclei in early and late pregnancy

**Fig. 1:**
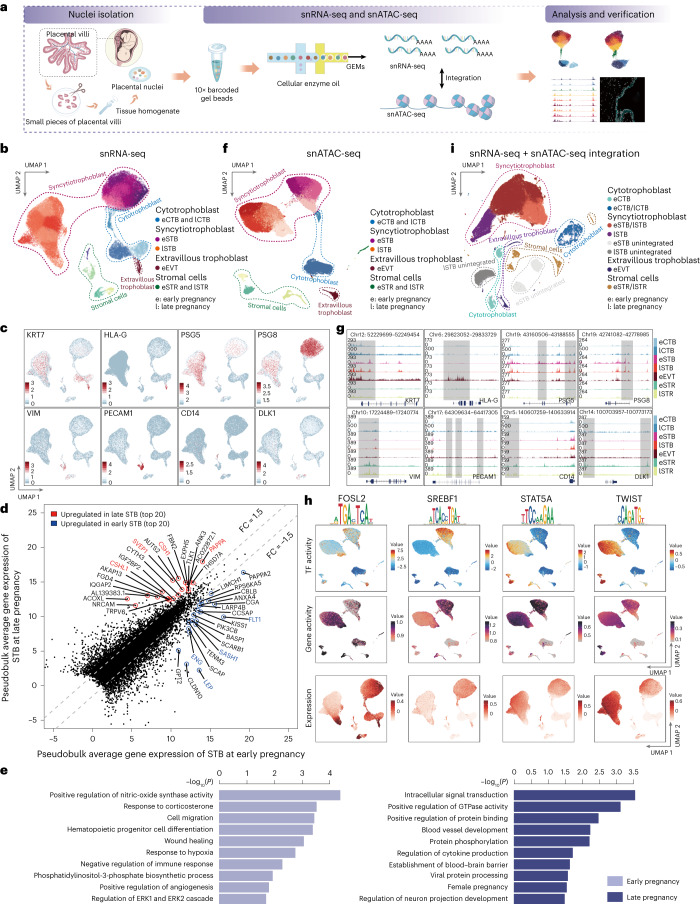
Single-nucleus transcriptomics and chromatin accessibility profiling of human placentas in early and late pregnancy. **a**, Schematic representation of the samples used in this study, nuclear isolation, sequencing experiments, downstream bioinformatic analyses and experimental validation. **b**, UMAP embeddings of snRNA-seq nuclei where dots correspond to individual nuclei. **c**, Annotation of the snRNA-seq clusters based on stage information and marker gene expression project on UMAP embeddings. The relative expression levels are presented with color intensities. Gene expression raw counts were normalized by depth, logarithmized and *z* score scaled, and finally smoothed with imputation. **d**, Scatter plot shows DEGs in the STB nuclei in early and late pregnancy. DEGs are identified with the Wilcoxon test (*P*_adj_ ≤ 0.01, log_2_(FC) ≥ 0.25). Top 20 genes are highlighted and labeled. **e**. GO enrichment associated with DEGs for the STB nuclei in early and late pregnancy. GO enrichment analysis is tested by one-sided Fisher’s exact test with a default *P* < 0.1. **f**. UMAP embeddings of snATAC-seq nuclei where dots correspond to individual nuclei. Abbreviation rules as in **b**. **g**, snATAC-seq genomic tracks along denoting chromatin accessibility peaks of marker genes for each cluster. Gray boxes highlight specific accessible regions associated with marker genes. **h**, TFs overlay on snATAC-seq UMAP (TF activity scores (top) and gene-activity scores (middle)) and snRNA-seq (gene expression level (bottom)) for TF genes, *FOSL2*, *SREBF1*, *STAT5A* and *TWIST*. The TF activity, gene activity and relative expression levels are presented with color intensities. **i**. The integrated UAMP plot of STB nuclei from both the snRNA-seq data and the snATAC-seq data. Abbreviation rules as in **b**. eSTB unintegrated, unintegrated snRNA-seq data and the snATAC dataset from eSTB nuclei in early pregnancy; lSTB unintegrated, unintegrated snRNA-seq data and the snATAC dataset from eSTB nuclei in late pregnancy. Source data

To comprehensively understand the human placental nuclear repertoire and its regulatory mechanisms, we applied 10x Genomics snRNA-seq and snATAC-seq techniques to nuclei isolated from 12 healthy placentas, six each in early (6–9 weeks of gestation) and late pregnancy (38–39 weeks of gestation). We took measures to eliminate potential sex bias by collecting an equal number of placentas from each sex before sequencing (Supplementary Fig. 1a,c). Detailed information on the sample collection is presented in Supplementary Table 1 and Supplementary Note 1. A graphical overview of the methodology is presented in Fig. 1a.

After stringent filtering, we retained 50,850 nuclei and 50,693 nuclei for snRNA-seq and snATAC-seq, respectively, for subsequent analyses (Supplementary Table 1; Methods). Sample preparation batch correction methods were applied to both datasets, resulting in strong correlation (Supplementary Fig. 1d) and high-quality data (Supplementary Fig. 1e,f). Batch-corrected transcriptomic and epigenomic datasets were clustered using the Scanpy^34^ and SnapATAC v2 (ref. ^35^) package accordingly and visualized using uniform manifold approximation and projection (UMAP) dimension reduction (Fig. 1b,f). Seven major nuclear clusters were annotated using combined pregnancy stage information and canonical markers, identifying early and late populations of CTB (eCTB, lCTB), STB (eSTB, lSTB) and stromal cells (eSTR, lSTR), as well as an early EVT (eEVT)—e for early pregnancy and l for late pregnancy (Fig. 1c, Supplementary Fig. 1g–i and Supplementary Note 2). The ratio of spliced and unspliced RNA was relatively similar among those annotated nuclear types, indicating that nuclei in the STB have comparable transcriptional capabilities to other cell types (Supplementary Fig. 1b and Supplementary Note 3). Interestingly, CTB, EVT and STR nuclei at different gestational stages clustered closely together, while STB nuclear clusters exhibited distinct differences between early and late pregnancy, suggesting greater functional disparities (Fig. 1b).

Differentially expressed genes (DEGs) and gene ontology (GO) analysis were conducted on the STB nuclei during early and late pregnancy to identify functional differences (Fig. 1d,e, Supplementary Table 2 and Supplementary Note 4). We found the enrichment of GO terms in the early pregnancy-related STB DEGs included cell migration, response to hypoxia and positive regulation of angiogenesis. Those functions are crucial for the proper function of the human placenta in a physiological low-oxygen environment in early pregnancy before the maternal blood flows into the intervillous space^2,14^. In late pregnancy, enrichment of GO terms included positive regulation of GTPase activity, viral protein processing and intracellular signal transduction. These GO functions together with others might reflect the need for transportation, resistance against viral infection and maximum nutrient requirements for successful fetal delivery.

We next sought to determine whether dynamic STB gene expression in early and late pregnancy correlated with accessible chromatin sites. We analyzed the snATAC-seq data and detected consistent nuclear types based on gene-activity scores (a quantitative measure specific to snATAC-seq data that signifies the extent of gene accessibility) of marker genes from our snRNA-seq analysis, including eCTB, lCTB, eSTB, lSTB, eEVT, eSTR and lSTR (Fig. 1f). Cluster annotation based on gene-activity score was validated by aggregated snATAC-seq tracks around marker genes for each cluster (Fig. 1g and Supplementary Fig. 1j). Notably, the UMAP analysis revealed distinct distributions of STB clusters in early and late pregnancy, a pattern reminiscent of the findings obtained from snRNA-seq analysis. These results indicate a consistent gene expression and chromatin accessibility in placental nuclei in early and late pregnancy, reflecting varied regulatory mechanisms and functions in the STB at different gestational stages.

To identify potential TFs that may regulate dynamic STB nuclear gene expression and functions during different gestation stages, we used chromVAR to identify enriched TF binding motifs from the JASPAR 2020 database that were present within the accessible chromatin^36^. Motif enrichment analysis revealed gestation stage-specific enrichment of binding motif on several TFs, including FOSL2, SREBF1, HES2, CEBPA, HIF1A and MITF in early pregnancy and STAT5A, TWIST, STAT6, GCM1, RORA and NR3C2 in late pregnancy (Fig. 1h and Supplementary Fig. 1k). We further demonstrated concordance by direct transcript measurement from the snRNA-seq dataset, inferred gene-activity scores representing the degree of gene accessibility from the snATAC-seq dataset and TF activity using chromVAR, which identified enriched binding motifs of key TFs on the UMAP (Fig. 1h and Supplementary Fig. 1k). In addition, we observed specific enrichment of the HIF1A binding motif in STB during early pregnancy, suggesting potential responsiveness to HIF1A. This could be in response to the normal low-oxygen concentration in the intervillous space in the early placenta, but HIF1A can also be activated by growth-promoting kinases^37,38^. It is worth mentioning that oxygen levels in the early placenta, although relatively low compared to those in the later stages of pregnancy, still fall within the normal range. We also identified STAT6 and STAT5A as key TFs in STB in late pregnancy. STAT signaling pathway was reported to be involved in regulating PAPPA, a serum marker used in screening for developmental abnormalities, in vitro^39^.

The correlation between chromatin accessibility and downstream gene expression signatures emphasizes the potential of integrating snATAC-seq with snRNA-seq data. To accomplish this, we combined several recently developed methods, which are as follows: (1) we removed the batch effects using Harmony^40^; (2) we analyzed our snATAC-seq datasets in early and late pregnancy using SnapATAC v2 (ref. ^35^), and snRNA-seq datasets were analyzed through Scanpy^34^; and (3) the final merged snRNA-seq and snATAC-seq data were further integrated using liger (Supplementary Fig. 2a)^41^. Our analysis revealed that nuclear clusters classified based on chromatin data and transcriptome data were grouped together in the integrated UMAP space (Fig. 1i, Supplementary Fig. 2b–d and Supplementary Note 5).

### Nuclear heterogeneity and a differentiation trajectory bifurcation in the human placental STB in early pregnancy

**Fig. 2:**
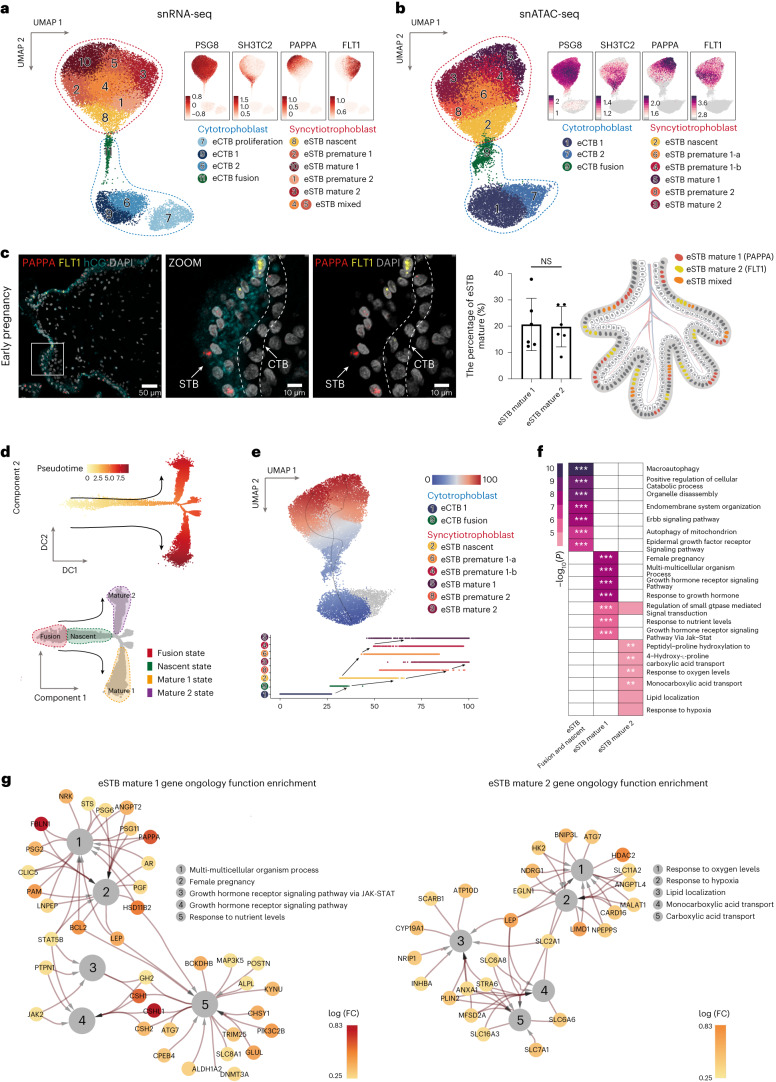
The heterogeneity of CTB and STB nuclei in early pregnancy. **a**, UMAP shows CTB and STB nuclei profiled with snRNA-seq in early pregnancy. The upper right UMAP indicates the expression patterns of representative marker genes for STB nuclear subclusters. The expression levels are presented with color intensities. **b**, UMAP shows CTB and STB nuclei profiled with snATAC-seq in early pregnancy. The upper right UMAP indicates the gene-activity scores of representative marker genes for STB nuclear subclusters. The activity scores are presented with color intensities. **c**, smFISH staining of indicated marker genes (*PAPPA*, *FLT1* and hCG) characterizes eSTB mature 1 and eSTB mature 2 in early pregnancy. Statistical analysis of the proportion of eSTB mature 1 and eSTB mature 2 (middle). Data are shown as mean ± s.d. Unpaired two-tailed *t* test. *n* = 6 donors. The schematic represents the distribution of eSTB mix, eSTB mature 1 and eSTB mature 2 in the early placental STB (right). **d**, Pseudotime ordering of CTB and STB nuclei profiled with snRNA-seq reveals two differential trajectories (upper). The differentiation time is presented with color intensities. Quantitative classifications for each cluster by differentiation states are shown (bottom). **e**, Differential trajectories visualization on snATAC-seq UMAP (upper) and pseudotime ordering of the STB differentiation trajectories on snATAC-seq (bottom). The differentiation time is presented with color intensities. This trajectory was inferred by a supervised algorithm (Method). **f**, GO enrichment reveals STB nuclear functions. Three DEG sets of the two trajectories are used for the GO enrichment test. The −log_10_ *P* value of the enrichment is presented with color intensities. GO enrichment analysis is tested by one-sided Fisher’s exact test and *P* values are adjusted for multiple comparisons with the Benjamini–Hochberg method. **P_adj_ < 0.01; ***P_adj_ < 0.001. **g**, Functional networks visualize the GO enrichment results. Nodes represent GO terms and DEGs, whereas edges represent a gene’s membership in the GO term. Node color, log(FC) of DEGs; edge width, the signification of enrichment test *P* adjust value. GO enrichment analysis is tested with the same statistic method as **f**. NS, not significant. Source data

We initially observed the segregation of STB nuclei into subclusters using snRNA-seq, snATAC-seq and integrated UMAP (Fig. 1b,f,i). To investigate the details of nuclear heterogeneity within the STB during early pregnancy, we conducted a focused analysis of 23,702 nuclei from the eSTB and its progenitors, the eCTB, as shown in Fig. 1b. We identified ten subclusters based on snRNA-seq datasets with four eCTB subclusters (clusters 7, 9, 6 and 11, *DNMT1*, in early pregnancy) and six eSTB subclusters (clusters 8, 2, 10, 1, 3, 4 and 5, *PSG8* and *CGA*, in early pregnancy) (Fig. 2a and Supplementary Fig. 3a). For the eCTB subclusters, we annotated eCTB proliferation (cluster 7, *MKI67*), eCTB fusion (cluster 11, *ERVFRD-1*) and two intermediate clusters, eCTB1 (cluster 9) and eCTB2 (cluster 6). Our eCTB subclusters annotations aligned with those of a previous study^27^. The consistency between scRNA-seq and snRNA-seq results confirmed the reliability of our analytical method.

Interestingly, we observed the distinct gene expression patterns in the eSTB nuclear subclusters (Fig. 2a and Supplementary Fig. 3a). Cluster 8, which expressed *SH3TC2*, was located in proximity to cluster 11 (eCTB fusion) on the UMAP plot and was annotated as eSTB nascent. Other nuclei, with low *SH3TC2* expression and high *LEP* expression, were considered nuclear subclusters of the eSTB that gradually matured. Clusters 10 and 3, located at the edge of the UMAP, were considered the most matured STB clusters and were annotated as eSTB mature 1 (cluster 10 with highly expressed *PAPPA*) and eSTB mature 2 (cluster 3 with highly expressed *FLT1*). Clusters 2, 1, 4 and 5 were annotated as premature or mixed STB nuclei, with lower expression of *PAPPA* or *FLT1* than nulcei in cluster 10 or cluster 3, and were annotated as eSTB premature 1 (cluster 2), eSTB premature 2 (cluster 1) and eSTB mixed (clusters 4 and 5). Overall, our results demonstrate considerable transcriptional heterogeneity within the STB nuclei in early pregnancy. To make cell/nuclear type nomenclature clear, we have included a table outlining the abbreviations, full names and detailed explanations of the eSTB nuclear subclusters (Supplementary Table 3).

To investigate the correlation between chromatin accessibility and eSTB nuclear gene expression, we analyzed snATAC-seq data for 22,785 eSTB and eCTB nuclei. Nine nuclear clusters were identified based on their gene-activity scores of known markers identified in snRNA-seq (Fig. 2b and Supplementary Fig. 3b). Notably, our analysis showed high concordance between the assigned subclusters in the snATAC-seq and snRNA-seq datasets, except for the proliferative eCTB cluster, potentially due to the loss of accessible chromatin during mitosis (Fig. 2a,b)^42,43^. We further confirmed the presence of distinct subtypes of STB nuclei during early pregnancy through single-molecule fluorescence in situ hybridization (smFISH; Fig. 2c and Supplementary Note 6). Overall, our multi-omic analysis revealed the heterogeneous nature of STB nuclei in early pregnancy at both the gene expression and epigenetic levels, which was missed by previous scRNA-seq analyses of placentas.

The maturation and development of STB nuclei remain poorly understood although there are suggestions that the STB nuclei may be of varying ages^22^. Pseudotime trajectory analysis of snRNA-seq data and differentiation pseudotime from snATAC-seq data, using a supervised method described in ref. ^44^, suggested the existence of a bifurcated developmental trajectory rooted from a common progenitor population for STB nuclei (eSTB nascent) toward two mature populations, namely eSTB mature 1 and eSTB mature 2. Notably, there is also a premature state that connects these STB subclusters at the transcriptional and epigenetic levels, which has not been described previously (Fig. 2d,e, Supplementary Fig. 3c and Supplementary Note 7).

Specifically, DEGs and GO enrichment analysis unveiled the involvement of nascent STB nuclei in diverse biosynthetic processes, possibly coordinated by the ERBB and EGFR signaling cascades. Interestingly, eSTB mature 1 nuclei were associated with various growth hormone signaling pathways (Fig. 2f,g, Supplementary Fig. 3d–g, Supplementary Table 4 and Supplementary Note 8), while eSTB mature 2 nuclei are linked to the oxygen response, lipid localization and carboxylic acid transportation.

Taken together, our results demonstrated nuclear and functional heterogeneity, as well as the route by which these STB nuclei differentiate during early pregnancy.

### Subtype-specific enhancer-gene regulatory network in STB in early pregnancy

**Fig. 3:**
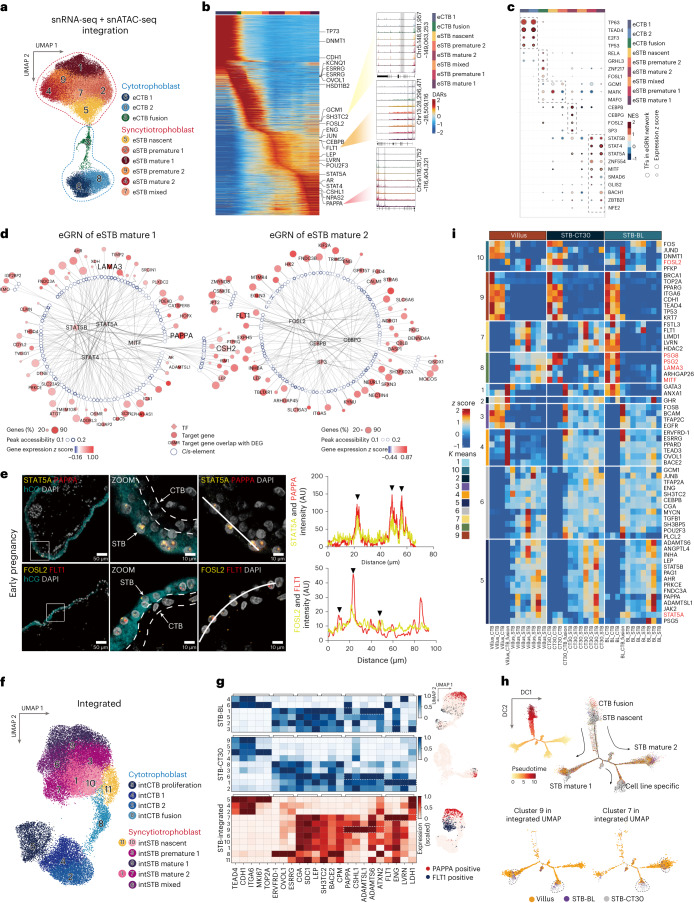
Nucleus-type-specific *cis*-regulation architecture of CTB and STB in early pregnancy. **a**, UMAP shows CTB and STB nuclei profiled with integration of snRNA-seq and snATAC-seq in early pregnancy. **b**, Heatmap shows pseudotime ordering of the 23,746 DARs in early pregnancy (left). Zoomed-in genomic tracks show the *cis*-element accessibility of representative genes. The normalized accessibility of DARs is presented with color intensities. **c**, TF-mining heatmap shows candidate master TFs of each nucleus type. The NES and expression *z* score are presented with dot color and dot size, respectively. Dots with bold edges show selected TFs used for network construction. **d**, Regulatory networks with three layers (TF, *cis*-element and target gene) represent the *cis*-regulatory architecture covering the complete STB differentiation process. The gene percentage, peak accessibility and gene expression score are presented with circle sizes, edge width and color intensities, respectively. **e**, smFISH staining (left) and fluorescence intensities (right) show the colocalization of TFs (*STAT5A* and *FOSL2*) and representative genes (*PAPPA* and *FLT1*) of eSTB mature 1 and eSTB mature 2. hCG, STB marker. The white dotted lines show the outline of CTB. The white lines represent the plotted tracks of fluorescence intensity. **f**, UMAP shows CTB and STB nuclei profiled with integration of snRNA-seq of STB-CT30, STB-BL and placenta villi in early pregnancy. **g**, Heatmaps show marker gene expression in STB-BL, STB-CT30 and placental villi in early pregnancy. The expression levels are presented with color intensities. Binarized gene expression levels (positive or negative) are calculated and visualized beside heatmaps for the two marker genes, *PAPPA* and *FLT1*. **h**, Pseudotime ordering shows three differential trajectories of CTB and STB nuclei (upper left). The differentiation time is presented with color intensities. Quantitative classifications for each cluster (upper right) and specific pseudotime of cluster 9 and cluster 7 on the integrated UMAP (bottom) are shown. **i**, Heatmap shows the different expression patterns of marker genes, master TFs and representative hormones among in vitro trophoblast models and in vivo placental villi in early pregnancy. The *z* scores are presented with color intensities. Examples of genes with similar expression patterns are highlighted in bold black, and those with different patterns are colored in red. Source data

To further investigate the regulatory mechanisms driving the nuclear heterogeneity and lineage specification of eSTB, we performed cross-modality integrative analyses using liger^45^ and divided all eSTB nuclei into nine clusters based on the integrated analysis (Fig. 3a). Major STB nuclear subtypes identified by jointed datasets are comparable to those identified by snATAC-seq or by snRNA-seq separately (Supplementary Fig. 5a,b and Supplementary Note 9).

We sought to determine the master regulators of distinct STB nuclear subtypes and delineate their regulatory landscape along the inferred differentiation trajectories in early pregnancy. We used MACS2 to identify candidate *cis*-regulatory elements (cCREs)^46^. To accomplish this, we conducted peak calling for each cell type individually. Subsequently, we merged all identified peaks, resulting in the identification of a total of 274,189 *cis*-elements across all CTB subclusters and STB subclusters. Among these elements, ~8.66% (23,746) exhibited nuclear-subtype-specific accessibility (Fig. 3b, Supplementary Fig. 4, Supplementary Table 5 and detailed descriptions are shown in Supplementary Note 10). Accessibility dynamics of cCREs associated to genes *SH3TC2*, *FLT1*, *PAPPA*, *ESRRG*, *OVOL2*, *STAT5A*, *HIF1A* and *CEBPB* along the STB nuclear differentiation path of STB nuclei were revealed (Fig. 3b, Supplementary Fig. 4 and Supplementary Note 10). Potential TFs that may serve as master regulators in guiding the differentiation of STB nuclei into two trajectories were also nominated (Fig. 3c and Supplementary Note 11).

To link the nuclear-specific master TFs regulated gene networks and enhancers for the key TFs (enhancer-gene regulatory network (eGRNs)), we calculated the correlation between gene expression and region accessibility for a symmetrical window of 500 kb around each gene transcription start site, leading to an average of six regions per gene and a total of 100,682 raw peak-to-gene links^44^ (Supplementary Fig. 5c). We further examined focused eGRNs for the eCTB fusion, eSTB mature 1 and eSTB mature 2 populations (Fig. 3d, Supplementary Fig. 3e and Supplementary Table 6), including 12 activator TFs with 2,081 enhancers linked to 1,566 genes. Those cistrome TFs we highlighted here, including ZNF217, RELA, MAFK and GRHL3 for eCTB fusion, STAT5A, STAT5B, STAT4 and MITF for eSTB mature 1 and FOSL2, CEBPB, CEBPG and SP3 for eSTB mature 2, may act as master lineage-determining regulators (Fig. 3d and Supplementary Fig. 5d,e). MITF enhancers were linked to several known hormone genes such as *CSH2* and *LEP*. Subcellular colocalization of *STAT5A* with *PAPPA* and *FOSL2* with *FLT1* supported their cooperative function in defining eSTB mature 1 and eSTB mature 2 gene expression and function (Fig. 3e).

### hTSCs and trophoblast organoids based on hTSCs could partially imitate placental STB heterogeneity, regulatory mechanisms and function

The derivation of hTSCs by us and others represent experimentally tractable and gene-editable in vitro model systems for examining aspects of human placental development^47^. However, the extent to which those models recapitulate the heterogeneity of the CTB and the STB nuclear subtypes, as well as the associated regulatory mechanisms we have identified in vivo, remains largely unexplored. Therefore, we used our large snRNA-seq and snATAC-seq datasets from human early placentas as references to better characterize these in vitro models. We used two hTSC cell lines derived from a human early blastocyst (hereinafter referred to as hTSCs-BL)^47^ and first-trimester placental CTBs (hereinafter referred to as hTSCs-CT30)^48^ to show the generalizability of our findings (Supplementary Note 12).

To determine multinucleated STB features in vitro, we conducted snRNA-seq analysis of STB derived from both hTSCs-BL and hTSCs-CT30 (hereinafter referred to as STB-BL and STB-CT30, respectively). We integrated in vitro and in vivo snRNA-seq data from placentas to assess whether our in vitro trophoblast models (hTSCs-BL, hTSCs-CT30, STB-BL and STB-CT30) could mimic the CTB subtypes and STB nuclear subclusters we identified in this study. Using canonical trophoblast markers identified in vivo, we annotated newly generated nuclear/cellular subclusters in the integrated datasets including intCTB proliferation, intCTB1, intCTB2 and intCTB fusion subclusters, as well as intSTB nascent, intSTB premature 1, intSTB mature 1/mature 2 and intSTB mixed subclusters (Fig. 3f,g,i, Supplementary Fig. 6a–c and Supplementary Note 13). However, eSTB mature 1 and eSTB mature 2 were not observed in STB-BL and STB-CT30 as revealed along the integrated differentiation trajectory (Fig. 3h, Supplementary Fig. 6a–f and Supplementary Note 13).

**Fig. 4:**
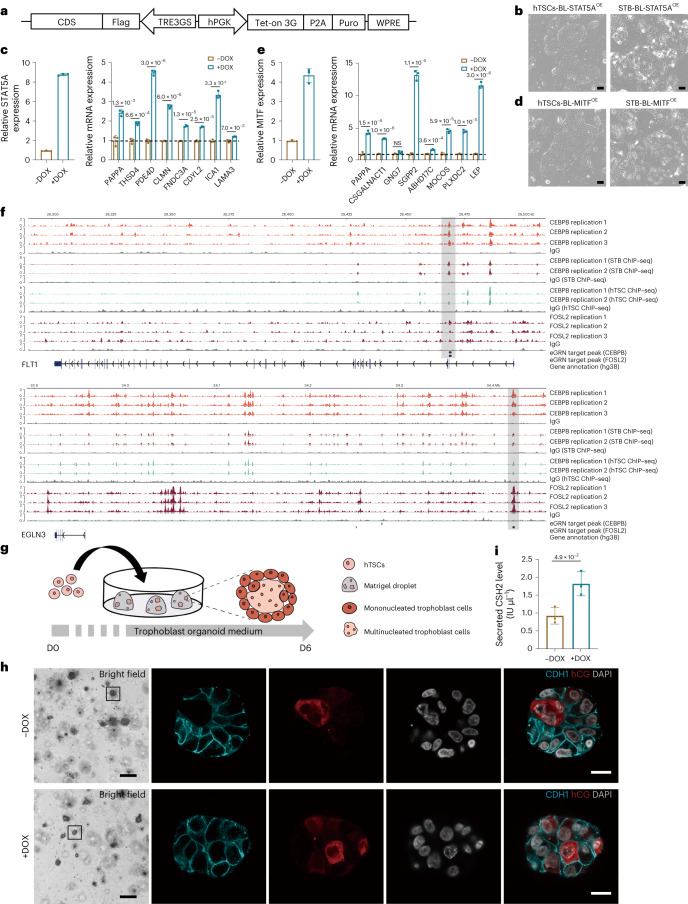
Characterization of similarity of hTSCs and placental organoids when compared to placental villi in early pregnancy. **a**, The schematic illustrates the lentiviral vector used for *STAT5A* and *MITF* overexpression. **b**, Bright-field images show hTSCs-BL-STAT5A^OE^ and STB-BL-STAT5A^OE^. Scale bar: 100 μm. **c**, RT–qPCR analysis of STAT5A and target gene expression in STB-BL with DOX-inducible overexpression of *STAT5A*. Data are shown as mean ± s.d. *P* values by multiple unpaired two-tailed *t* test. *n* = 3 independent experiments. The dotted lines in **c** and **e** represent the baseline 1. **d**, Bright-field images show hTSCs-BL-MITF^OE^ and STB-BL-MITF^OE^. Scale bar: 100 μm. **e**, RT–qPCR analysis of *MITF* and target gene expression in STB-BL with DOX-inducible overexpression of *MITF*. Data are shown as mean ± s.d. *P* values by multiple unpaired two-tailed *t* test. *n* = 3 independent experiments. **f**, Verification and visualization of eGRN enhancer-gene regulatory events near two target genes of two STB mature 2 specific TF regulators, CEBPB and FOSL2. Genomic tracks are calculated and compared with CEBPB and FOSL2 CUT&Tag, CEBPB ChIP–seq datasets, which are prepared with STB-BL and hTSC-BL cell lines. IgG mock is used for negative control. Regulatory events are highlighted with the following criteria: eGRN target gene regions (enhancers) should overlap STB-BL CUT&Tag peak(s) and/or ChIP–seq peak(s) but are depleted in hTSC-BL. **g**, Schematic representation of trophoblast organoids derivation. hTSC-BL are transferred to matrigel droplets on day 0 (D0) and maintained in trophoblast organoid medium to analyze on day 6 (D6). **h**, Bright fields and immunofluorescence analysis of trophoblast markers (*CDH1* and hCG) in trophoblast organoids derived from hTSCs-BL with DOX-inducible overexpression of *MITF*. Scale bars: 250 μm for bright-field images, 20 μm for immunofluorescence images. **i**, ELISA analysis of *CSH2* expression in trophoblast organoids derived from hTSCs-BL with DOX-inducible overexpression of *MITF*. Data are shown as mean ± s.d. *P* values by unpaired two-tailed *t* test. *n* = 3 independent experiments. CDS, coding sequence; hPGK, human phosphoglycerate kinase 1 promoter; P2A: porcine teschovirus-1 2A peptide; Puro, puromycin; TRE3GS, TRE3GS inducible promoter; Tet-on 3G, Tet-on 3G transactivator protein; WPRE, Woodchuck hepatitis virus’s post-transcriptional regulatory element; OE, overexpression. Source data

We investigated the regulatory mechanisms between in vitro and in vivo STB in hTSCs-BL and hTSCs-CT30 with doxycycline (DOX)-inducible overexpression of *STAT5A* and *MITF*. The full-length CDS with a Flag-tag was used due to low expression levels of these genes in in vitro STB compared to their in vivo counterparts in eSTB mature 1 (Fig. 4a–e and Supplementary Table 7). We next aimed to determine whether regulatory mechanisms are conserved between in vitro and in vivo STB. The significant increase in the gene expression such as *PAPPA* and others (Supplementary Note 14) under STAT5A and MITF overexpression in hTSC-BL confirmed our eGRN as shown in Fig. 3d. Similar results were obtained for hTSC-CT30 (Supplementary Fig. 6h–k). CUT&Tag and chromatin immunoprecipitation followed by sequencing (CHIP–seq) experiments were performed and confirmed the consistent regulatory events between the eGRN in placental STB in early pregnancy and STB differentiated from hTSCs (Fig. 4f and Supplementary Note 14). To test these in vitro models, we investigated the role of MITF in the developing trophoblast organoids constructed from hTSCs-BL. We generated trophoblast organoids using hTSCs-BL with DOX-inducible overexpression of MITF. ELISA assays showed a positive correlation between CSH2 secretion and the overexpression of MITF in the trophoblast organoids (Fig. 4g–i).

Overall, our findings indicate that our datasets offer a reliable reference for evaluating in vitro models recapitulating certain aspects of STB heterogeneity, regulatory mechanisms and functions during early pregnancy.

### Matured STB nuclear heterogeneity and function were revealed in late pregnancy

**Fig. 5:**
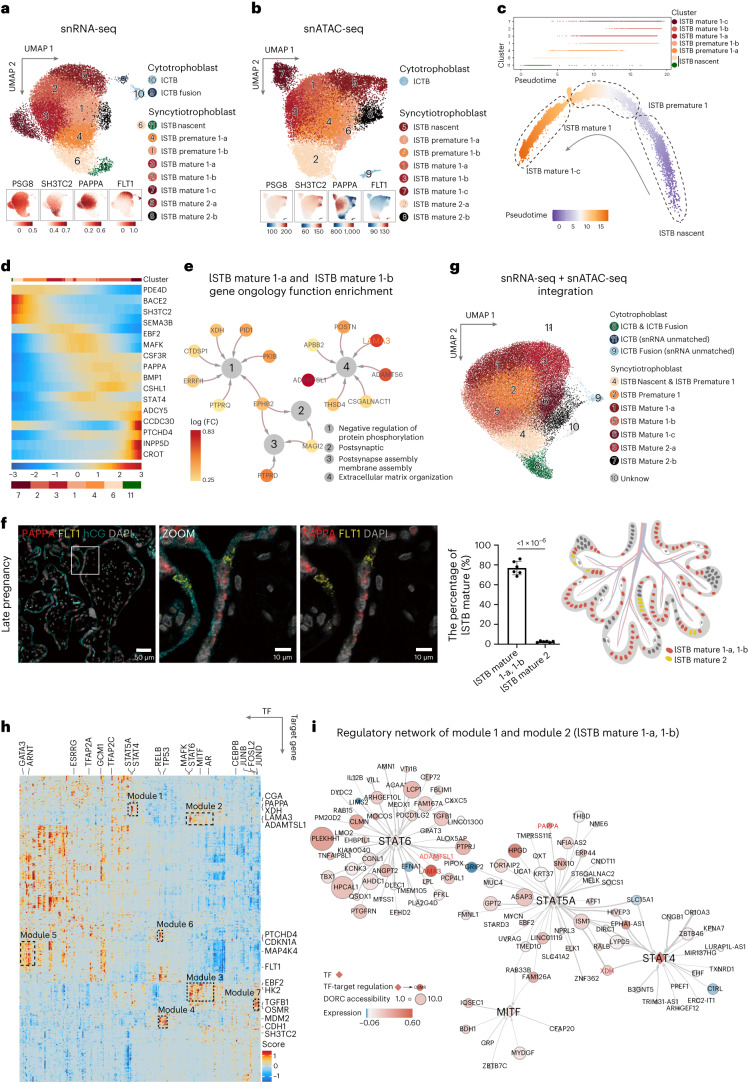
lSTB mature 1 dominates the STB nuclear subclusters in late pregnancy. **a**, UMAP shows CTB and STB nuclei profiled with snRNA-seq in late pregnancy. The bottom UMAP indicates the expression patterns of representative marker genes for STB nuclear subclusters in late pregnancy. The expression levels are presented with color intensities. Gene expression raw counts were normalized by depth, logarithmized and *z* score scaled, and finally smoothed with imputation. **b**, UMAP shows CTB and STB nuclei profiled with snATAC-seq in late pregnancy. The bottom UMAP indicates the gene-activity scores of representative marker genes for STB nuclear subclusters in late pregnancy. The activity scores are presented with color intensities. **c**, Pseudotime ordering of lSTB nuclei profiled with snRNA-seq shows the differentiation trajectory in late pregnancy by monocle 2 DDRTree algorithm. The differentiation time is presented with color intensities. Nuclei of each cluster are aligned above and ordered by pseudotime values. **d**, Trajectory heatmap shows 16 typical new marker genes (row) expressed dynamically along pseudotime ordering (column). The pseudotime time is presented with color intensities. **e**, Functional networks visualize the GO enrichment results. Nodes represent GO terms and DEGs, whereas edges represent a gene’s membership in the GO term. Node color, log(FC) of DEGs; edge width, the signification of enrichment test *P* adjust value. GO enrichment analysis is tested by one-sided Fisher’s exact test and *P* values are adjusted for multiple comparisons with the BH method. **f**, smFISH staining of indicated marker genes (*PAPPA*, *FLT1* and hCG) characterizes lSTB mature 1 and lSTB mature 2 in late pregnancy (left). Statistical analysis of the proportion of lSTB mature 1 and lSTB mature 2 (middle). Data are shown as mean ± s.d. *P* values by unpaired two-tailed *t* test. *n* = 6 donors. The schematic represents the distribution of lSTB mature 1-a, lSTB mature 1-b and lSTB mature 2 in the late placental STB (right). **g**, UMAP shows lCTB and lSTB nuclei profiled with snRNA-seq and snATAC-seq after the python package 'GLUE' integration (Methods). **h**, Heatmap shows the activity of TF-target regulatory modules elicited from the integration of snRNA-seq and snATAC-seq data using the FigR package. Genes represent target genes and candidate TF regulators in rows and columns, respectively. **i**, TF-regulatory network construction with a combination of modules lSTB mature 1-a and lSTB mature 1-b in late pregnancy. The Domains of Regulatory Chromatin (DORC) accessibility and expression level are presented with circle sizes and color intensities, respectively. The network is built with a different method for late pregnancy. Source data

To systematically investigate the development of human placental STB in late pregnancy, we examined our large-scale single-nucleus transcriptomic (23,981 nuclei) and epigenomic (24,692 nuclei) datasets from lSTB and lCTB nuclei. Quality control (Supplementary Fig. 7a–d) and annotation of lCTB subtypes are described in Supplementary Note 15. Gene sets previously used to classify nuclear types in early pregnancy demonstrated notable consistency in late pregnancy samples (Fig. 5a,b, Supplementary Fig. 7e,f and Supplementary Table 3). Cluster 11 and cluster 6 were classified as two nascent nuclear types of STB in late pregnancy with high expression of *SH3TC2* (lSTB nascent 1 and lSTB nascent 2). *PAPPA* expression was substantially increased in late pregnancy and was present in five STB subclusters, including cluster 4 (lSTB premature 1-a), cluster 1 (lSTB premature 1-b), cluster 3 (lSTB mature 1-a), cluster 2 (lSTB mature 1-b) and cluster 7 (lSTB mature 1-c). In contrast, *FLT1* expression was only detected in cluster 5 (lSTB mature 2-a) and partially in cluster 8 (lSTB mature 2-a), which represents a small fraction of nuclei in STB in late pregnancy (Fig. 5a and Supplementary Fig. 7e). Detailed information on the annotation of STB nuclear subtypes is shown in Supplementary Notes 16 and 17. The decreasing expression of FLT1-positive STB nuclei may indicate an intriguing regulatory effect of the altered environmental cues from hypoxia in early pregnancy to normoxia in late pregnancy and dynamic developmental demands. Interestingly, the small population of FLT1-expressing lSTB nuclei showed dramatic closure of the chromatin locus around the *FLT1* gene when compared with early pregnancy (Supplementary Fig. 8b,d). The low chromatin accessibility around the *FLT1* gene expression locus during late pregnancy led us to consider detectable FLT1 RNA in clusters 5 and 8 as residual RNA of the *FLT1* gene from an earlier pregnancy stage.

To further uncover the molecular mechanisms driving lSTB nuclear heterogeneity and place the newly annotated lSTB nuclear subclusters in a defined trajectory, we performed pseudotime analysis (Fig. 5c,d). A linear transcriptional trajectory was revealed for all PAPPA-positive nuclei in late pregnancy. Following the order of pseudotime, we observed that the first cluster of lSTB nuclei was largely composed of nascent nuclei we annotated with specific gene expression of *PDE4D*, *BACE2* and *SH3TC2* (Fig. 5c and Supplementary Fig. 7h–j). lSTB nuclei with high expression of *LAMA3* and located at the end of the trajectory were the most matured nuclear type with *BMP1* highly expressed nuclei located in the middle (Fig. 5c and Supplementary Fig. 7h–j). We further revealed dynamic gene expression throughout a continuum of nuclear-state transition in Fig. 5d and identified DEGs along the trajectory, including *PDE4D*, *SH3TC2*, *PAPPA*, *BMP1*, *STAT5A*, *PTCHD4*, *CCDC30*, *INPP5D* and *CROT*. GO enrichment analysis revealed that extracellular matrix organization (ECM) was highly associated with nuclei in lSTB mature 1-a and lSTB mature 1-b subcluster (Fig. 5e and Supplementary Table 8).

Placenta in late pregnancy was stained with probes targeting genes *PSG8*, *SH3TC2*, *LEP*, *PAPPA* and *FLT1*. The smFISH results for *FLT1* and *PAPPA* suggest that a substantial portion of STB nuclei exhibit *PAPPA* expression, aligning with our bioinformatic analysis (Fig. 5f and Supplementary Note 17).

The integration of snRNA-seq and snATAC-seq through analysis of peaks and gene regulatory proximity to the linear genome reveals strong connections of gene expression patterns and accessibility dynamics of regulatory elements (Fig. 5g, Supplementary Fig. 9a–g and Supplementary Note 18). Subsequently, we performed a TF-mining and TF-regulatory network analysis (FigR, v0.1.0) and identified seven unique modules of genes that are regulated by distinct TFs (Fig. 5h,i and Supplementary Figs. 10 and 11). Notably, key TFs that were enriched in the most matured and functional lSTB mature 1-a and lSTB mature 1-b subclusters, such as STAT6, STAT5A, STAT4 and MITF (module 1 and module 2 as shown in Figs. 5h and 5i), were also important in the eSTB mature 1 subcluster in early pregnancy. GO enrichment revealed a strong association between ECM and the nuclear subtypes of STB in the late pregnancy stage (Supplementary Note 19).

## Discussion

Recently, nuclear-specific functions in the syncytia of various organisms, including the fungus *Ashbya gossypii*^49^, muscle fibers^50^ and other tissue systems, have been documented. However, the nuclear heterogeneity and differentiation in the human placental STB remains largely unexplored^16,18,19,22,51^. Here we leveraged the power of snRNA-seq and snATAC-seq to profile the nuclear diversity and regulatory mechanism from both transcriptomic and epigenetic perspectives. Our analysis demonstrated the dynamic STB nuclear subtypes during early and late pregnancy. It provided evidence of significant heterogeneity in the STB nuclei from a gene expression and regulation perspective, allowing us to identify a dynamic bifurcating trajectory of STB nuclei with distinct biological functions. These findings together with the eGRNs we uncovered lay the groundwork for further exploration of mechanisms driving human placental STB development in supporting successful pregnancy (Supplementary Note 20).

Specifically, integrative snRNA-seq and snATAC-seq analysis of STB nuclear populations in early pregnancy, revealed some TFs, including STAT5A, that might serve as a driving force in guiding the differentiation of STB nuclei towards a more mature state, which is characterized by high levels of *PAPPA* expression. PAPPA, a serum marker used for screening developmental abnormalities in early pregnancy, was detected at increasing concentrations in the maternal circulation during pregnancy^52^. We anticipate that future work will clarify the relationship of TFs we identified here and the conditions associated with PAPPA. Moreover, a direct regulatory network FLT1 controlled by CEBPB and FOSL2 was revealed. This is especially important in the clinical context, as CEBPB was recently reported to be involved in EVT dysfunction in the severe preeclampsia placenta^53,54^. The abnormally overexpressed soluble FLT1 protein in the placenta, which is secreted into maternal blood, is also known to contribute to the pathogenesis of preeclampsia^55^. Therefore further investigation is necessary to fully understand the regulatory roles of CEBPB and FOSL2 in FLT1 expression in the STB during pregnancy and in related diseases.

Newly established hTSCs have been considered a substantial breakthrough in the field^48,56–58^. Our previous work has already proven that hTSCs could be used as a model for human trophoblast differentiation during implantation^59^. Manipulation of the key STB nuclear lineage-determining TFs that we identified here, in hTSCs, could provide a better experimental model in the interpretation of human placenta STB in later stages beyond implantation.

In summary, we have presented a comprehensive characterization of STB nuclear heterogeneity during pregnancy (see the summary model in Supplementary Fig. 13). The data we generated not only enhanced our understanding of the regulatory mechanisms that govern the dynamic development of STB nuclear identities but also established a valuable framework for future studies aimed at interpreting the roles of key molecules associated with placental development and disorders in humans.

## Methods

### Description of human placental donors and ethical approval

The placental specimens used in this study of snRNA-seq and snATAC-seq were collected from six early pregnant women (6–9 weeks of pregnancy) and six late pregnant women (38–39 weeks of pregnancy). Additional three healthy placental tissues from the three early pregnant women (7–8 weeks of pregnancy) and three placental tissues from the three late pregnant women (38 weeks of pregnancy) were used for experimental validation (RNAscope). The placental specimens were all collected from Peking University Third Hospital. Sex identification for samples was analyzed with PCR of the following two sex-linked genes: SRY and NLGN4, before sequencing. This research was approved by the Ethics Committee of the Institute of Zoology, the Chinese Academy of Sciences, and the Ethics Committee of the Peking University Third Hospital under research license (2019) JLS (242-01). All patients participating in the study were healthy pregnant women without any exclusion. Every patient involved in the project has signed the informed consent. All the patients in our research project shared one ethical approval license number.

### Library construction and sequencing

#### Single-nucleus isolation

The villi were separated from the early or term placenta and cut up after removing the blood with PBS. Ice-cold Nuclei EZ lysis buffer (Sigma-Aldrich, NUC-101) containing RNAase inhibitor (Takara, 2313B) and protease inhibitor (Sigma-Aldrich, P8340) was added to resuspend the tissue and grinded with a Dounce tissue grinder (Sigma-Aldrich, D8938). The lysates were bathed in ice and lysed twice. After washing with PBS, the nuclei were split in half and filtered through a 40 μm sieve to proceed with ATAC-seq and RNA-seq.

#### snRNA-seq

Single Cell B Chip Kit (10x Genomics, 1000074) and the nucleus suspension (600 nuclei per microliter determined by CountStar) were loaded onto the Chromium single cell controller (10x Genomics) to generate single-nucleus gel beads in the emulsion (GEMs) according to the manufacturer’s protocol. In brief, single nuclei were suspended in PBS containing 0.04% BSA. About 16,000 nuclei were added to each channel, with 8,000 target nuclei estimated to be recovered. Captured nuclei were lysed, and the released RNA was barcoded through reverse transcription in individual GEMs. Reverse transcription was performed on an S1000TM Touch Thermal Cycler (Bio-Rad) at 53 °C for 45 min, followed by 85 °C for 5 min and held at 4 °C. The cDNA was generated and then amplified, and quality was assessed using an Agilent 4200 (performed by CapitalBio Technology). Next, snRNA-seq library was constructed using Single Cell 3′ Library and Gel Bead Kit V3.1 according to the manufacturer’s instructions. The libraries were finally sequenced using an Illumina NovaSeq 6000 sequencer with a sequencing depth of at least 100,000 reads per nuclei with a pair-end 150 bp (PE150) reading strategy (performed by CapitalBio Technology).

#### snATAC-seq

Following the 10x Genomics single-cell ATAC solution, by using Chromium Chip E Single Cell Kit (1000156) and Chromium Single Cell ATAC Library and Gel Bead Kit (1000110), the nuclei in a bulk sample were partitioned into nanoliter-scale GEMs and a pool of ~750,000 10× Barcodes was sampled to separately and uniquely index the transposed DNA of each nucleus. Libraries were then generated (performed by CapitalBio Technology). The libraries were sequenced using an Illumina Novaseq sequencer with a sequencing depth of at least 25k read pairs per nuclei with a pair-end 50 bp (PE50) reading strategy.

### Massive integration analysis of snRNA-seq and snATAC-seq from two pregnancy periods

#### Identification of nucleus types at a global level

To understand the nuclei type consistent of snRNA-seq and snATAC-seq of the early and late pregnancy, we started the analysis with the Cellranger ‘aggre’ command (without dept normalization) to aggregate libraries. For a quick and general analysis, we used the Python packages Scanpy v1.6.1 (ref. ^34^) and SnapATAC v2.2.0 (ref. ^35^) to perform the upstream analysis. Then each data modality was applied with Harmony^40^ to remove the batch effect, and we then achieved dimension reduction, nuclei cluster partition and annotation with known marker genes. We used customized R codes to visualize the results. To assist cluster annotation in snATAC-seq, the gene-activity score of each gene was quantified by make_gene_matrix function from the package SnapATAC. For integrating the 24 libraries from two modalities at two pregnancy stages, the liger package (v0.5.0)^41^ was finally applied. The nuclear major types from different modalities and pregnancy stages were visualized by marker genes in the integration space.

#### DEG identification of the STB population

To understand the diversity of the STB nuclei in early and late pregnancy, we identified the DEGs according to the snRNA-seq analysis results. We first subset all STB nuclear clusters from the early and late pregnancy data and performed DEG test at the single-nucleus level with the following three methods: Seurat findMarkers (Wilcoxon test, default parameter), Scanpy rank_gene_groups (Wilcoxon test, default parameter) and method from ref. ^60^ (analysis of variance (ANOVA), fold change (FC) > 0.5, *P* < 0.05). We then found a shared set within the top 200 of each sorted DEG gene sets. To directly visualize and compare the gene expression difference between pregnancy stages, we aggregated and calculated a pseudobulk RNA-seq data by stage and donor. We then averaged logarithm of counts per million reads, log_2_(CPM) expression values of the six donors per stage and plotted with scatter plot and labeled the top 20 genes. GO of Biological Process enrichment was analyzed with DAVID^61^ of the knowledge base v2023q2 with share DEG genes.

#### Differential TF identification of the STB population

We applied chromVAR^62^ to perform a quick scan for snATAC-seq data of two pregnancy stages against the JASPAR 2020 motif database^36^. In brief, the genomic positions of each motif were identified in each peak. A deviation score of each motif was calculated for each nucleus, producing a deviation score by nuclei matrix. Then this matrix was *z*-score normalized. We used FindMarkers in the Seurat package to identify differentially enriched motifs with default parameters between early and late pregnancy. TF motifs of interest were selected from the top 50 list and further aligned and visualized with gene-activity score and gene expression data.

### snRNA-seq analysis for STB subtypes in early pregnancy

#### Dimension reduction and graphic clustering for nucleus-type annotation

We used the R package Seurat (v3)^63^ to conduct the general upstream analysis. Generally, we used principal component analysis for dimension reduction with a maximum dimension of 30. We then performed graphic clustering by the ‘Louvain’ algorithm with a start resolution of 0.9. The final resolution was chosen after an iterative tuning for best interpreting of the biological complexity of the placenta sample. We annotated each cluster by marker gene expression.

#### Differential gene expression analysis and GO enrichment analysis

To identify DEGs, we applied Seurat FindAllMarkers with parameters of min.pct = 0.1, logfc.threshold = 0.25. Top 50 DEGs were visualized by heatmap. Clusterprofiler (v3.18.1) was applied for GO enrichment analysis and visualized by customized R script. All DEGs were saved and used for further analysis.

#### Differentiation trajectory reconstruction

We used the R package Monocle2 (ref. ^64^) to conduct pseudotime analysis. DEGs identified before were used as the informative gene set, which would encode the cell differential trajectory. To focus on the STB nuclei differentiation, we excluded the CTB nuclei and started at the fusion-competent CTB nuclei (ERVFRD-1 positive). We set DEGs mentioned above as ordering genes for unbiased trajectory inference. The trajectory tree was then visualized as ‘DDRTree,’ and essential genes during differentiation were examined by heatmap. Each nuclear cluster type was classified along the trajectory path and visualized by customized R scripts.

### snATAC-seq analysis for STB subtypes in early pregnancy

#### Dimension reduction and graphic clustering for nucleus-type annotation

We applied the SnapATAC v2 (ref. ^35,65^) package to perform the general upstream analysis because we found this package used a spectral clustering method as the dimension reduction algorithm, which can better interpret the intrinsic population structure of the STB clusters. Harmony^40^ was applied for batch effect removal. We also compared the dimension reduction results with cisTopic (v0.3.0) with the Latent Dirichlet Allocation (LDA) algorithm, ArchR (v1.0.1) with the iterative Latent Semantic Indexing (LSI) algorithm and Signac (v1.1.0) with the LSI algorithm (data not shown). All these tools produced similar dimension reduction results. For nuclei clustering, we applied the ‘Leiden’ algorithm with a resolution of 0.9 for the initiative graphic clustering. To gain reasonable cluster numbers (which is essential to interpret the nuclei types), we iteratively tested the resolution to optimize the final clustering results. Several known marker genes were used to verify and annotate the clusters.

#### Differentially accessible regions (DARs) identification

We used the function ‘rank_genes_groups’ in the scanpy package with ‘*t* test’ methods to identify DARs. This function used a raw peak count matrix as the input data. We applied a strict filtering threshold of *P* value < 0.001 and a minimal log_2_(FC) of 1. The top 5,000 DARs were kept and used for further analysis.

#### Differentiation trajectory reconstruction

We applied a supervised trajectory inference method^44^ to illustrate the developmental paths of STB nuclei in early pregnancy. Briefly, we tried to fit a candidate trajectory path on a UMAP map and then pseudotime values were assigned to each nucleus. It is based on the hypothesis that UMAP coordinates per se harbor the intrinsic differential information.

### Matching snRNA-seq and snATAC-seq clusters in early pregnancy

#### Calculate gene-activity score for snATAC-seq nuclei

We aggregated and counted the snATAC-seq Tn5 insertion sites within the gene body and a 2 kb extended upstream region to calculate the gene-activity score for each gene of the human gene annotation database (Gencode v41) by SnapATAC v2 function make_gene_matrix.

#### Clusters matching with liger

Similar to the previous massive integration analysis, we applied the liger method for modality integration of the early pregnancy data. We selected the variable genes set from snRNA-seq data with var.thresh = 0.2 and set the *k* = 35. Finally, the Integrative Non-negative Matrix Factorization (iNMF) dimension reduction matrix was calculated with all nucleus ids as the col-names. Louvain clustering was then conducted with resolution = 0.8.

To visualize the integration results, we merged every liger modality dataset and built a Seurat object with both gene-activity score and gene expression data. Then we normalized the data slot with ‘normalizeData’ function in Seurat. We visualized the marker genes in the integration space to annotate liger clusters. snRNA-seq clusters and snATAC-seq clusters were joined and paired according to the integration clusters. A confusion matrix of cluster pairs was also made from paired nuclei with annotation labels in previous results.

#### Quantify and visualize the between-modality alignment result

We applied a customized K-Nearest Neighbors (KNN) algorithm to quantify the nucleus-to-nucleus matching result. In brief, we first randomly picked 50 snATAC-seq nuclei in the liger integration UMAP space and then iteratively found the nearest snRNA-seq nuclei. The results were compared and visualized with a combination of snRNA-seq and snATAC-seq clusters by a scatter plot.

### Peak-to-gene linkage analysis

We used the liger iNMF dimension reduction matrix to pair nuclei of the snRNA-seq data to nuclei from the snATAC-seq data by a simple nearest Euclidean distance strategy^44,66^. This method found the closest RNA nuclei for each ATAC nuclei in the liger iNMF space and built the nuclei pairs. Accordingly, the pairing result was used to impute the expression matrix (gene × nuclei of snRNA) to the imputedRNA matrix (gene × nuclei of snATAC), resulting in two matrics with paired nucleus ids. Then all-to-all correlation was calculated among all gene rows of the imputedRNA matrix against all peak rows of the peak count matrix (peak × nuclei of snATAC). We then filtered the correlation table with a cutoff of correlation co-efficiency of 0.45 and an False Discovery Rate (FDR) cutoff of 0.0001. The resulting peak-versus-gene pairs were defined as peak-to-gene links. Then all peak-to-gene links were aligned with a combination of peak accessibility and gene expression data and visualized as a heatmap. To illustrate the local enhancer–promoter regulatory landscape of essential genes (*PAPPA* and *FLT1*), we plotted genomic regions around genes with the pseudobulk ATAC tracks matching single nuclear gene expression data.

### TF mining and construction of a regulatory network

To decipher the TF regulation network for STB with a multi-omics-based strategy^67^, we reinterpreted the core steps with homemade R codes with slight modifications to perform the TF mining and construct a precise and robust ‘TF—*cis*-element—target gene’ regulatory network (Supplementary Fig. 6g). In brief, the TF motif enrichment tool i-cisTarget^68^ and pycistarget^69^ were used to scan TF motif enrichment in previously identified cluster-specific DARs (coordinates were lifted from hg38 to hg19), with default parameters except for an area under the curve (AUC) threshold of 0.001. We used the motif Position Weight Matrix (PWM) database only. For pycistarget, we used the nonredundant motif database ‘hg38_screen_v10_clust’ for clearer TF gene assignment. The TF motif enrichment results of the above mentioned two packages were collected, merged and parsed, and a maximal normalized enrichment score (NES) was assigned to each TF motif that passed the AUC threshold 3.0. According to the hypothesis that a TF regulator is a true-positive TF regulator only if the TF gene expression is positively correlated with the accessibility of motifs^66^, we calculated Pearson’s correlation for all TF motif NES against TF gene expression. We ranked the correlations for TF genes and visualized all selected cluster-specific TF regulators in a heatmap.

To build the ‘TF—*cis*-element—target gene’ regulatory network, we extracted all candidate target genes for each TF motif in the enrichment table and searched for the linked peaks (*cis*-element) for each target gene within all predefined peak-to-gene links. The final networks were constructed with Cytoscape (v3.9.1)^70^, integrating node and edge attributions according to snRNA-seq or snATAC-seq data.

### Culture of hTSCs

The culture of human trophoblast was performed as described previously^47^. Briefly, the plate was coated with 5 μg ml^−1^ Collagen I (Corning) at 37 °C at least for 1 h. hTSCs were cultured in hTSCs medium (DMEM/F12 (Gibco) supplemented with 0.1 mM 2-mercaptoethanol (Gibco), 0.2% FBS (Gibco), 0.5% Penicillin–Streptomycin (Gibco), 0.3% BSA (Sigma-Aldrich), 1% ITS-X supplement (Gibco), 1.5 μg ml^−1^ l-ascorbic acid (Sigma-Aldrich), 50 ng ml^−1^ Epidermal Growth Factor (EGF, MedChemExpress), 2 μM CHIR99021 (MedChemExpress), 0.5 μM A83-01 (MedChemExpress), 1 μM SB431542(MedChemExpress), 0.8 mM valproic acid (VPA, Wako) and 5 μM Y27632 (MedChemExpress)). hTSCs were dissociated with TrypLE (Gibco) for 8 min at 37 °C, and the cells were passaged to a new Collagen I-coated plate. hTSCs were routinely passaged every 4–5 d at a 1:4–1:6 ratio.

### Lentivirus production

The full-length coding sequence of *STAT5A* and *MITF* were cloned into the pLVX-TetOne-TRE3GS-MCS-2A-PURO lentiviral vector, respectively. The overexpressing lentivirus vector together with packaging plasmids pPAX2 and pMD2.G were cotransfected into HEK293T cells using a polyethyleneimine transfection protocol (Proteintech). The viral supernatants were collected at 48 and 72 h post-transfection and then filtered through a 0.45 μm polyethersulfone (PES) filter.

### Generation of overexpressing hTSCs

hTSCs were preseeded into 12-well plate at a density of 5 × 10^4^ cells per well and were infected with overexpressing lentivirus at a confluency of 30% for 12 h. Then the infected hTSCs were treated with 2 μg ml^−1^ puromycin for 48 h to establish stable overexpressing hTSC lines.

### Differentiation of hTSCs into STB

hTSCs were grown to 80% confluence in the hTSCs medium and dissociated with TrypLE for 8 min at 37 °C. For the induction of STB, hTSCs were seeded in a six-well plate precoated with 2.5 μg ml^−1^ Collagen I at a density of 1 × 10^5^ cells per well and cultured in STB medium (DMEM/F12 supplemented with 0.1 mM 2-mercaptoethanol, 0.5% Penicillin–Streptomycin, 0.3% BSA, 1% ITS-X supplement, 2.5 μM Y27632, 2 μM forskolin (MedChemExpress) and 4% Knockout Serum Replacement (KSR, Gibco). The medium of overexpressing STB was supplemented with 5 μM DOX (MedChemExpress). The medium was replaced every 2 d and analyzed on day 6.

### Generation of trophoblast organoid

The establishment of trophoblast organoids was performed as described previously^71^. Briefly, dissociated hTSCs were suspended in 100 μl Matrigel at a density of 1 × 10^4^. One droplet per well was added to an eight-well ibidi chamber or eight droplets per well were added to a six-well plate. After setting the plate at 37 °C for 15 min, 200 µl or 2 ml hTSC culture medium was loaded per well. In this case, it is called trophoblast organoid medium. The medium of overexpressing organoids was supplemented with 5 μM DOX. The medium was replaced every 2 d and analyzed on day 6.

### Comparison of in vivo and in vitro snRNA-seq

We first analyzed and annotated two datasets of snRNA-seq obtained from hTSCs-BL and hTSCs-CT30 using the Seurat algorithm, similar to the analysis we performed on placenta samples in early pregnancy. We used known marker genes, including *GATA3*, *TEAD4*, *TOP2A*, *PCNA* and *ITGA6*, to annotate proliferative hTSC and CTB. In addition, we annotated STB-BL and STB-CT30 subclusters using markers that were previously used to annotate eCTB fusion (*ERVFRD-1*), eSTB nascent (*SH3TC2*) and eSTB mature (*CGA*, *PAPPA* and *FLT1*). We used the Seurat CCA algorithm^63^ to identify anchor genes and integrated two snRNA-seq datasets from hTSCs-BL and hTSCs-CT30 with snRNA-seq datasets from placental villus in early pregnancy. Subsequently, we jointly annotated the clusters using marker genes and aligned them along the differentiation trajectory using Monocle2 (v2.14.0)^64,72^.

### CUT&Tag

The experiment was conducted according to the instructions provided by Vazyme (TD904). In brief, approximately 5 × 10^4^ cells were collected and counted, then mixed with activated ConA beads and incubated overnight with primary antibodies (CEBPB—Proteintech, 23431-1-AP; FOSL2—USBiological, 351814; rabbit IgG—Abcam, ab172730). The secondary antibody (VazymeAb207) was incubated at room temperature with rotation for 1 h. After removing the supernatant, pA/G-Tnp Pro and TTBL were separately incubated with rotation at room temperature and 37 °C for 1 h. The supernatant was then mixed with DNA Extract Beads Pro to dissolve DNA with ddH_2_O and used directly for PCR amplification. Subsequently, VAHTS DNA Clean Beads were added to purify the DNA, which was then subjected to next-generation sequencing using the Illumina NovaSeq platform.

### ChIP–seq

The experiment of ChIP–seq was performed as previously described^73^. Briefly, about 5 × 10^6^ hTSCs-BL and STB-BL were used for cross-linking by 1% formaldehyde and quenched by 125 mM glycine. Cells were lysed and nuclei were sonicated and diluted to a final concentration of 0.1% by SDS. Protein A/G magnetic beads were added and incubated at 4 °C for 1 h. Precleared chromatin was incubated with primary antibody (CEBPB, 23431-1-AP, Proteintech) or monoclonal rabbit isotype control (Abcam, ab172730) overnight at 4 °C. Then protein A/G magnetic beads were added to capture the specific chromatin complexes. The supernatants were collected with 500 mM NaCl and incubated at 65 °C overnight for reverse cross-linking. Samples were incubated at 37 °C for 1 h with RNase A and were incubated at 50 °C for 1 h with proteinase-K. The released DNA was purified by the QIAquick PCR Purification Kit and processed for sequencing library preparation. Multiplexed ChIP–seq libraries were prepared by NEBNext Ultra II DNA Library Prep Kit (NEB, E7645S). The final libraries and multiplexed libraries were subjected to Illumina NovaSeq 6000 using PE150 (paired-end 150 nt) sequencing.

### Analysis of CUT&Tag and ChIP–seq data

We applied bowtie2 (v2.4.2) with default parameters for raw Fastq reads mapping to human (hg38) reference genome and used MACS2 to call peaks for CUT&Tag and ChIP–seq datasets. We used the ‘reduce’ function from the R package ‘GenomicRanges’ for the merge of peaks of each replication. We then intersected and counted overlap peaks from CUT&Tag and previous eGRN peaks that were assigned to nearby genes by peak-to-gene links. We quantitatively summarized the validation result by the overlap percentage of these two peak sets. Finally, we visualized the regulatory events with CUT&Tag, ChIP–seq tracks and eGRN TF-target peaks tracks for two randomly picked genes within the eGRN target gene list.

### smFISH with RNAscope

Fresh tissues were collected and fixed in 10% neutral-buffered formalin (Sigma-Aldrich) for 18 h at room temperature followed by dehydration in ascending series of ethanol, clearing in xylene, embedding in paraffin and section for smFISH with RNA probes targeting genes *SH3TC2*, *LEP*, *FLT1*, *PAPPA*, *PSG8*, *STAT5A* and *FOSL2* following instructions from RNAscope Multiplex Fluorescent Reagent Kit V2 (Advanced Cell Diagnostics, 323100). First, sections were pretreated with RNAscope hydrogen peroxide, the process of target retrieval and protease plus to unmask target RNA and permeabilize the cells. Second, probes were carefully prepared and used for the hybridization with the target RNA molecules. Third, RNAscope detection reagents were used to amplify the hybridization signals through sequential hybridization of amplifiers and label probes and stained with different fluorescent dyes. Finally, sections were counterstained with DAPI, mounted with fluorescent mounting medium and stored at −20 °C for long-term storage. Images were collected under a Zeiss LSM 880 confocal laser scanning microscope, and the image processing was performed with ZEN software. Additional methods are described in detail in Supplementary Methods.

### Statistics

#### Clustering result refinement

We tuned the clustering result with different graph construction parameters of *k* and community discovery methods (Louvain or Leiden) for snATAC-seq data. After a fix of *k* = 30 and setting the clustering algorithm as ‘Leiden,’ we iterated the community detection resolution and chose the most frequent stable cluster assignment. We then evaluate the cluster results by the Dunn index and Silhouette coefficient.

Statistical analysis was performed using GraphPad Prism software (v9.0.0) by unpaired *t* test or one-way ANOVA analysis. Results were shown as means ± s.d.

### Reporting summary

Further information on research design is available in the Nature Portfolio Reporting Summary linked to this article.

## Online content

Any methods, additional references, Nature Portfolio reporting summaries, source data, extended data, supplementary information, acknowledgements, peer review information; details of author contributions and competing interests; and statements of data and code availability are available at 10.1038/s41588-023-01647-w.

### Supplementary information

Supplementary Information Supplementary Figs. 1–13, Supplementary Notes 1–21, Supplementary Methods, Supplementary References and Supporting data for Supplementary Figs. 1, 4 and 9.

Reporting Summary

Supplementary Tables Supplementary Tables 1–9.

### Source data

Source Data Fig. 1 Gene expression matrix for visualization of stage-specific DEGs.

Source Data Fig. 2 smFISH staining of indicated marker genes (*PAPPA*, *FLT1* and hCG) characterizes eSTB mature 1 and eSTB mature 2 in early pregnancy.

Source Data Fig. 3 Normalized gene expression matrix for comparison of in vivo and in vitro STB population transcriptional signature.

Source Data Fig. 3 smFISH staining of TFs (*STAT5A* and *FOSL2*) and representative genes (*PAPPA* and *FLT1*) of eSTB mature 1 and eSTB mature 2.

Source Data Fig. 4 Immunofluorescence images of trophoblast markers (*CDH1* and hCG) in TO derived from hTSCs-BL and MITF-overexpressing hTSCs-BL.

Source Data Fig. 5 smFISH staining of indicated marker genes (*PAPPA*, *FLT1* and hCG) characterizes lSTB mature 1 and lSTB mature 2 in late pregnancy.

## Data Availability

Sequencing data generated for this study have been deposited in the Gene Expression Omnibus with the accession GSE247038. There are no restrictions on data availability or use. We used the human genome version hg38 for all the analysis steps. The processed results were deposited on the figshare repository, with links: https://figshare.com/s/61db5dc9030e6363267e (early pregnancy snATAC-seq datasets), https://figshare.com/s/ccec028e5a3ec5b20106 (early pregnancy snRNA-seq datasets), https://figshare.com/s/05fdcc5d0c6b46786047 (late pregnancy snATAC-seq datasets), https://figshare.com/s/5a435d5ea48b3d51b40b (late pregnancy snRNA-seq datasets) and https://figshare.com/s/40bc9295d533d163b738 (scRNA-seq and snRNA-seq datasets for cell lines). We also uploaded raw result tables for the construction of eGRN networks (https://figshare.com/s/a3ec1630f75de248698b). Source data are provided with this paper.

## Code availability

Codes for the generation of main result figures can be accessed at GitHub^74^ (https://github.com/wangmeijiao/Human_placenta_multi-omics_snRNA-seq_snATAC-seq; 10.5281/zenodo.10212321), and details are provided upon contact.
