# Supplementary material for: Single-nucleus multi-omic profiling of human placental syncytiotrophoblasts identifies cellular trajectories during pregnancy
Source: Nat Genet. 2024 Jan 24;56(2):294–305. doi: 10.1038/s41588-023-01647-w (PMC10864176; doi:10.1038/s41588-023-01647-w)
Supplement: Supplementary file 1 — Supplementary Figs. 1–13, Supplementary Notes 1–21, Supplementary Methods, Supplementary References and Supporting data for Supplementary Figs. 1, 4 and 9. [file 41588_2023_1647_MOESM1_ESM.pdf]

nature genetics

Article

https://doi.org/10.1038/s41588-023-01647-w

Single-nucleus multi-omic profiling of human placental syncytiotrophoblasts identifies cellular trajectories during pregnancy

In the format provided by the
authors and unedited

Supplementary Information Contents

1 Supplementary Figure 1-13 Page 2-26

2 Supplementary Note 1-21 Page 27-35

3 Supplementary Method Page 36-38

4 Supplementary Reference Page 39-40

5 Supplementary Figure Source Data Page 41-42

20 **Supplementary Figure**

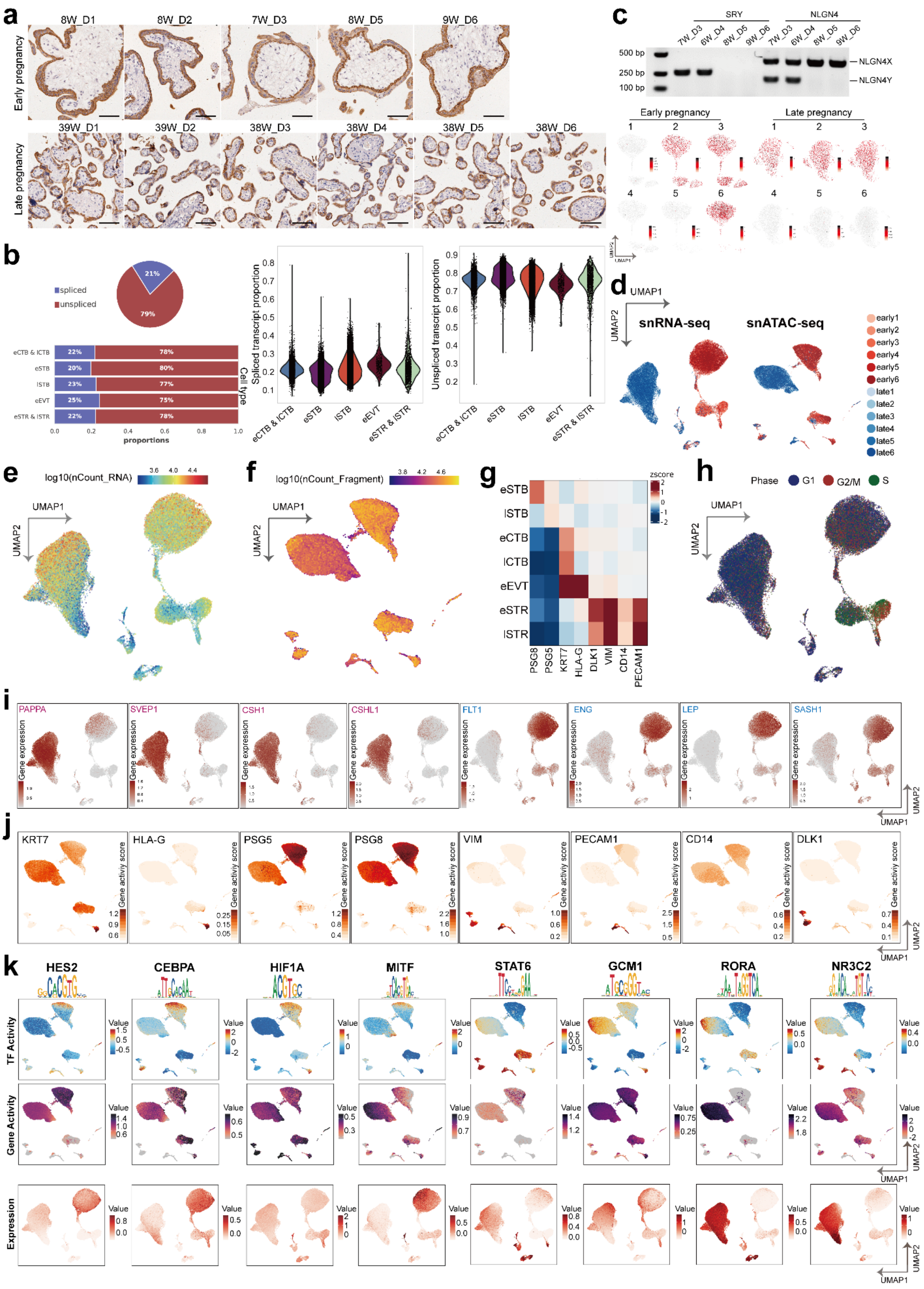

21

Supplementary Figure 1. Single-nucleus transcriptomics and chromatin accessibility profiling in the human in early and late pregnancy.

a. Immunohistochemistry staining of KRT7 shows the morphology of five of six placentas in early pregnancy and six placentas in late pregnancy used for sequencing. n=11 donors. Scale bars: 100 μm.

b. Pie chart shows total proportion of spliced and un-spliced transcripts (upper left). Bar chart shows the average proportion of spliced and un-spliced transcripts in each cell type (bottom left). Violin plots show the proportion of spliced (middle) and un-spliced (right) transcripts in nuclei of each cell type.

c. Gender identification for samples with 1) PCR of two sex-linked genes: SRY and NLGN4 before sequencing (upper) and 2) XIST gene expression after sequencing (lower, numbers refer to donor id). The expression levels are presented with color intensities.

d. UMAP shows the origin of donors for snRNA-seq and snATAC-seq. The abbreviation early1 refers to the data obtained from donor 1 in early pregnancy and similar naming conventions are used for other abbreviations.

e, f. UMAP shows the log10 normalized number of UMI counts (e) and fragment counts (f) of snRNA-seq and snATAC-seq data. The RNA count and fragment count are presented with color intensities.

g. Heatmap of scRNA-seq shows the expression of marker genes of trophoblast cells and stromal cells. The expression zscore is presented with color intensities.

h. UMAP shows the distinct cell cycle in the G1, G2/M, and S phases of snRNA-seq based on CellCycleScoring function of the Seurat package.

i. UAMP of scRNA-seq data shows the expression of early-vs-late DEGs. The expression levels are presented with color intensities.

j. UAMP of scATAC-seq data shows the identity of each cell cluster based on marker gene activity score. The activity score is presented with color intensities.

k. Transcription factors overlay on snATAC-seq UMAP (top) of gene-activity scores (middle) and snRNA-seq (bottom) for early pregnancy active TF genes (HES2, CEBPA, HIF1A, MITF) and late pregnancy active TF genes (STAT6, GCM1, RORA and NR3C2). The TF activity, gene activity and relative expression levels are presented with color intensities.

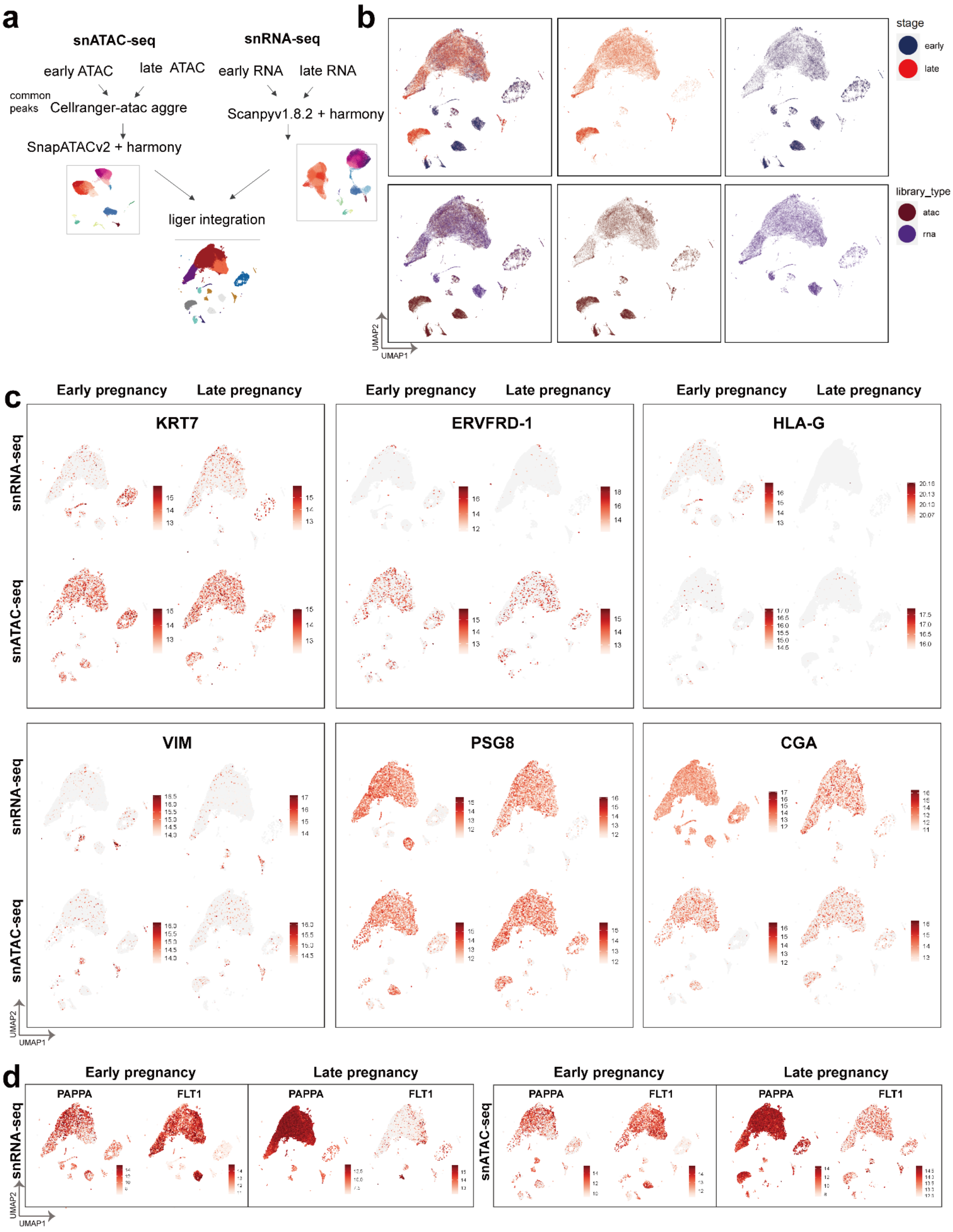
a
snATAC-seq
snRNA-seq
early ATAC
late ATAC
early RNA
late RNA
common peaks
Cellranger-atac aggre
Scanpyv1.8.2 + harmony
SnapATACv2 + harmony
liger integration
b
stage
early
late
library_type
atac
rna
UMAP2
UMAP1
c
Early pregnancy
Late pregnancy
snRNA-seq
snATAC-seq
KRT7
ERVFRD-1
HLA-G
VIM
PSG8
CGA
d
PAPPA
FLT1

Supplementary Figure 2. Massive integration of all twenty-four single nucleus multi-omics sequencing libraries in early and late pregnancy by liger.

a. Schematic diagram shows massive integration strategy for all 10X libraries.

b. UMAP shows integrated snATAC-seq data and snRNA-seq data colored by pregnant stage and dataset, as indicated on the right. early represents the placenta collected in early pregnancy. late represents the placenta collected in late pregnancy. atac represents snATAC-seq data. rna represents snRNA-seq data.

c. UAMP plots of integrated snATAC-seq data and snRNA-seq data show the identity of each cell cluster based on gene activity score and marker gene expression, respectively, in different pregnant stages and datasets. The expression levels are presented with color intensities.

d. UAMP plots of integrated snATAC-seq data and snRNA-seq data show the specific expression of FLT1 and PAPPA in different pregnant stages and datasets. The expression levels are presented with color intensities.

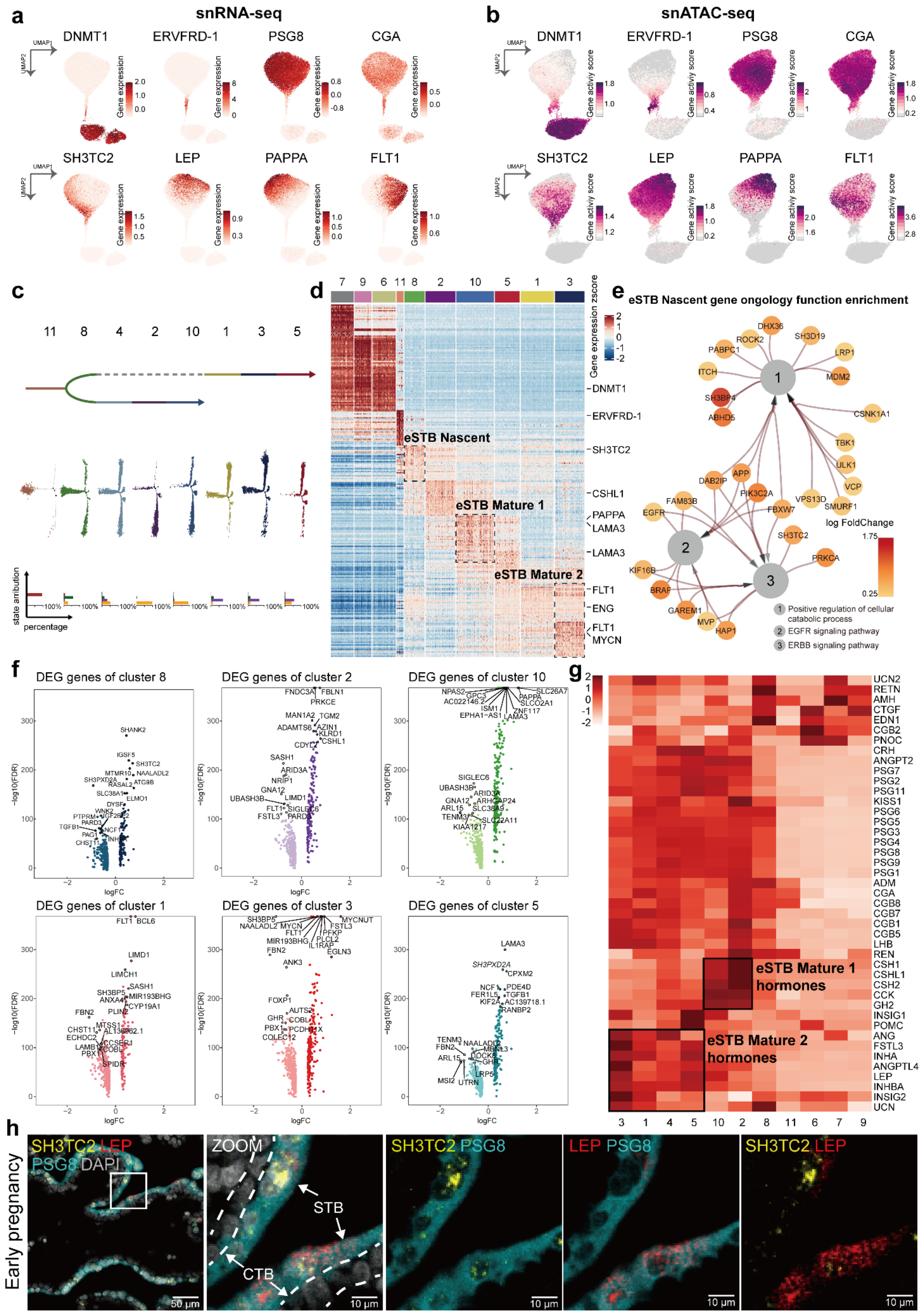

a
snRNA-seq
DNMT1
ERVFRD-1
PSG8
CGA
SH3TC2
LEP
PAPPA
FLT1
Gene expression
b
snATAC-seq
Gene activiy score
c
11 8 4 2 10 1 3 5
state arribution
percentage
d
7 9 6 11 8 2 10 5 1 3
Gene expression zscore
DNMT1
ERVFRD-1
SH3TC2
CSHL1
PAPPA
LAMA3
LAMA3
FLT1
ENG
FLT1
MYCN
eSTB Nascent
eSTB Mature 1
eSTB Mature 2
e
eSTB Nascent gene ongology function enrichment
log FoldChange
Positive regulation of cellular catabolic process
EGFR signaling pathway
ERBB signaling pathway
f
DEG genes of cluster 8
DEG genes of cluster 2
DEG genes of cluster 10
DEG genes of cluster 1
DEG genes of cluster 3
DEG genes of cluster 5
-log10(FDR)
logFC
g
eSTB Mature 1 hormones
eSTB Mature 2 hormones
UCN2
RETN
AMH
CTGF
EDN1
CGB2
PNOC
CRH
ANGPT2
PSG7
PSG2
PSG11
KISS1
PSG6
PSG5
PSG3
PSG4
PSG8
PSG9
PSG1
ADM
CGA
CGB8
CGB7
CGB1
CGB5
LHB
REN
CSH1
CSHL1
CSH2
CCK
GH2
INSIG1
POMC
ANG
FSTL3
INHA
ANGPTL4
LEP
INHBA
INSIG2
UCN
3 1 4 5 10 2 8 11 6 7 9
h
Early pregnancy
SH3TC2 LEP
PSG8 DAPI
ZOOM
STB
CTB
SH3TC2 PSG8
LEP PSG8
SH3TC2 LEP
50 μm
10 μm

Supplementary Figure 3. Distinct CTB and STB nuclear subclusters were identified in early pregnancy.

a. Marker gene expression level projected on the UMAP for the snRNA-seq dataset. The expression levels are presented with color intensities.

b. Marker gene activity scores projected on the UMAP for the snATAC-seq dataset. The activity scores are presented with color intensities.

c. Two-dimensional embeddings (with the DDRTree algorithm) of selected trophoblast nuclei (CTB fusion and STB) show nuclei distribution and quantitative summary along the pseudotime trajectory (related to Figure 2c).

d. Heatmap visualization of differentially expressed genes that were used in Gene Ontology enrichment analysis (related to Figure 2e,f). The gene expression zscore is presented with color intensities.

e. Functional network visualizes the Gene Ontology enrichment in genes of eSTB Nascent. Nodes represent GO term and DEGs and edges represent the signification of enrichment test. Node size: foldchange of DEGs, edge width: *p*.adjust value. The foldchange is presented with color intensities. GO enrichment analysis was tested by one-sided Fisher's exact test and p-values were adjusted for multiple comparison with the Benjamini-Hochberg (BH) method.

f. Volcano plots show the positive and negative differentially expressed genes with significant logarithms Foldchange (logFC) and logarithms False Discovery Rate (logFDR). Positive and negative differential expressed genes are identified for each snRNA-seq cluster, comparing expression level of each cluster to the average.

g. Hormone genes show diverged expression patterns on the heatmap, supporting additional evidence for functional diversity of STB nuclei. The expression level is presented with color intensities.

h. smFISH staining of indicated marker genes (SH3TC2, LEP and PSG8) on human placenta in early pregnancy, indicating the differences among SH3TC2 positive nuclei, PSG8 positive nuclei, and LEP positive nuclei.

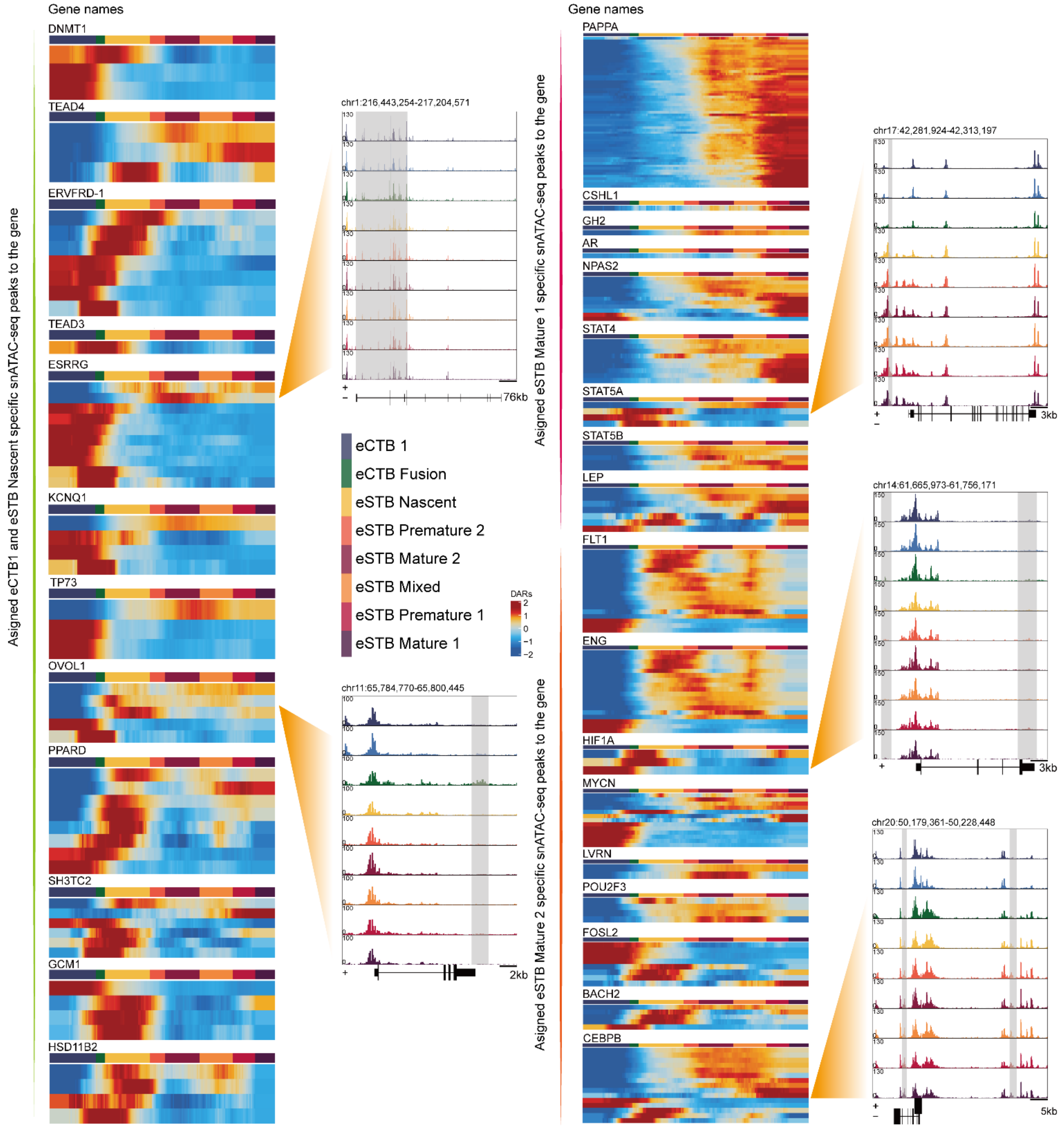
Gene names
DNMT1
TEAD4
ERVFRD-1
TEAD3
ESRRG
KCNQ1
TP73
OVOL1
PPARD
SH3TC2
GCM1
HSD11B2
Asigned eCTB1 and eSTB Nascent specific snATAC-seq peaks to the gene
chr1:216,443,254-217,204,571
76kb
chr11:65,784,770-65,800,445
2kb
eCTB 1
eCTB Fusion
eSTB Nascent
eSTB Premature 2
eSTB Mature 2
eSTB Mixed
eSTB Premature 1
eSTB Mature 1
DARs
Asigned eSTB Mature 1 specific snATAC-seq peaks to the gene
Asigned eSTB Mature 2 specific snATAC-seq peaks to the gene
Gene names
PAPPA
CSHL1
GH2
AR
NPAS2
STAT4
STAT5A
STAT5B
LEP
FLT1
ENG
HIF1A
MYCN
LVRN
POU2F3
FOSL2
BACH2
CEBPB
chr17:42,281,924-42,313,197
3kb
chr14:61,665,973-61,756,171
3kb
chr20:50,179,361-50,228,448
5kb

Supplementary Figure 4. Representative chromatin accessibility of *cis*-elements which locate near important genes along the STB differentiation trajectory.

Cis-elements were ordered by pseudotime value of snATAC-seq trajectory and assigned to the closest gene(s) by distance. Three types of cis-element linked genes were shown: genes enrich CTB and eSTB Nascent high accessible cis-elements (left); genes that show eSTB Mature 2 (top right) specific and genes that show STB Mature 1(bottom right) specific cis-elements. The chromatin landscape around genes of interest (ESRRG, OVOL1, STAT5A, HIF1A, and CEBPB) was plotted right to the heatmap. (Related to Fig. 3b). The DARs are presented with color intensities.

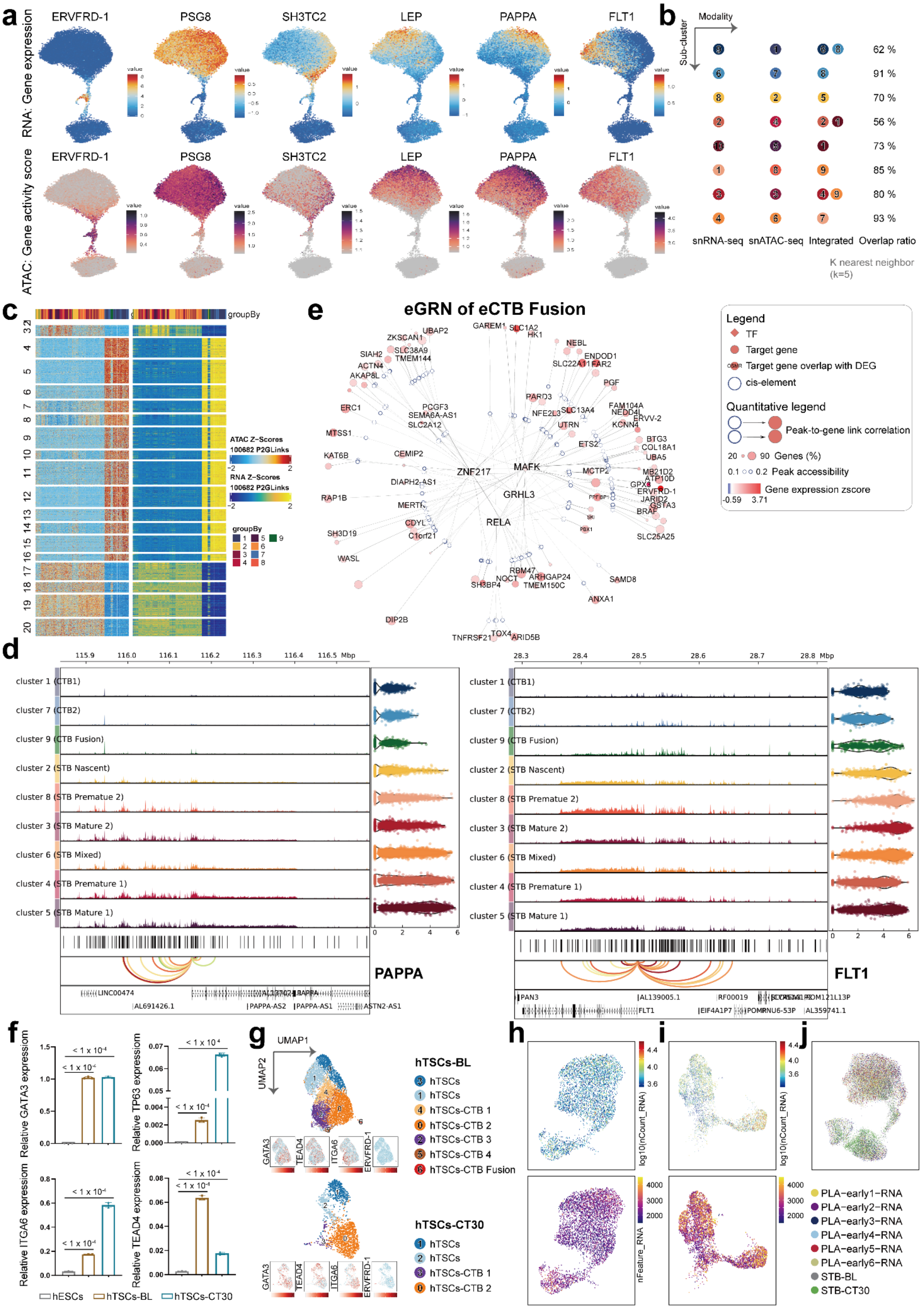

a
RNA: Gene expression
ATAC: Gene activity score
ERVFRD-1
PSG8
SH3TC2
LEP
PAPPA
FLT1
b
Modality
Sub-cluster
62 %
91 %
70 %
56 %
73 %
85 %
80 %
93 %
snRNA-seq
snATAC-seq
Integrated
Overlap ratio
K nearest neighbor (k=5)
c
groupBy
ATAC Z-Scores 100682 P2GLinks
RNA Z-Scores 100682 P2GLinks
e
eGRN of eCTB Fusion
Legend
TF
Target gene
Target gene overlap with DEG
cis-element
Quantitative legend
Peak-to-gene link correlation
Genes (%)
Peak accessibility
Gene expression zscore
ZNF217
MAFK
GRHL3
RELA
d
cluster 1 (CTB1)
cluster 7 (CTB2)
cluster 9 (CTB Fusion)
cluster 2 (STB Nascent)
cluster 8 (STB Prematue 2)
cluster 3 (STB Mature 2)
cluster 6 (STB Mixed)
cluster 4 (STB Premature 1)
cluster 5 (STB Mature 1)
PAPPA
FLT1
f
Relative GATA3 expression
Relative TP63 expression
Relative ITGA6 expression
Relative TEAD4 expression
hESCs
hTSCs-BL
hTSCs-CT30
g
UMAP1
UMAP2
hTSCs-BL
hTSCs
hTSCs-CTB 1
hTSCs-CTB 2
hTSCs-CTB 3
hTSCs-CTB 4
hTSCs-CTB Fusion
hTSCs-CT30
h
i
j
log10(nCount_RNA)
nFeature_RNA
PLA−early1−RNA
PLA−early2−RNA
PLA−early3−RNA
PLA−early4−RNA
PLA−early5−RNA
PLA−early6−RNA
STB-BL
STB-CT30

Supplementary Figure 5. Identification of transcription factor regulators and construction of TF regulatory network during STB nuclei differentiation in early pregnancy.

a. UMAP shows gene expression and activity score of marker genes, which are presented with color intensities.

b. Quantitative summaries of cluster matching reveals good agreement of liger integration of snRNA-seq and snATAC-seq clusters. A 'K nearest neighbor algorithm' was used to quantify the matching (see method).

c. Heatmap shows 100,882 peak-to-gene links by alignment of peak accessibility (left) and imputated gene expression (right). Data of both matrics are Z-score transformed to the same scale and split into 25 groups by the hierarchy clustering algorithm. The 'groupBy' annotation color bar shows cluster names in Figure 2b. The snATAC peak accessibility zscore and snRNA imputation value are presented with color intensities.

d. Illustration of peak-to-gene links with two marker genes, PAPPA (left) and FLT1 (right). Local accessible chromatin landscape is visualized by pseudo-bulk snATAC-seq tracks, along with matched gene expression data (horizontal violin plots) at single nucleus level. Gene annotation is plotted below. Transcription direction is indicated by arrows.

e. The eGRN regulatory network of eSTB Fusion (related to Figure 3d). The gene percentage, peak accessibility and gene expression score are presented with circle sizes, circle edge widths and color intensities, respectively.

f. Quantitative gene expression analysis of GATA3, TP63, ITGA6, and TEAD4 for hESCs, hTSCs-BL, and hTSCs-CT30. Data are shown as mean ± s.d. *P*.value by one-way ANOVA analysis. n = 3 independent experiments.

g. UMAP embeddings and annotations of hTSCs-BL and hTSCs-CT30 profiled with scRNA-seq. UMAP indicates the expression patterns of representative marker genes for CTB subclusters. The expression levels are presented with color intensities.

h, i. UMAP shows the log normalized number of UMI counts (upper) and captured genes (bottom) for snRNA-seq of STB-BL (f) and STB-CT30 (g). The RNA counts are presented with color intensities.

j. The distribution of snRNA-seq nuclei from *in vitro* STB cell lines STB-BL, STB-CT30 and *in vivo* placental villus in early pregnancy of six donors on a Seurat CCA integration UMAP.

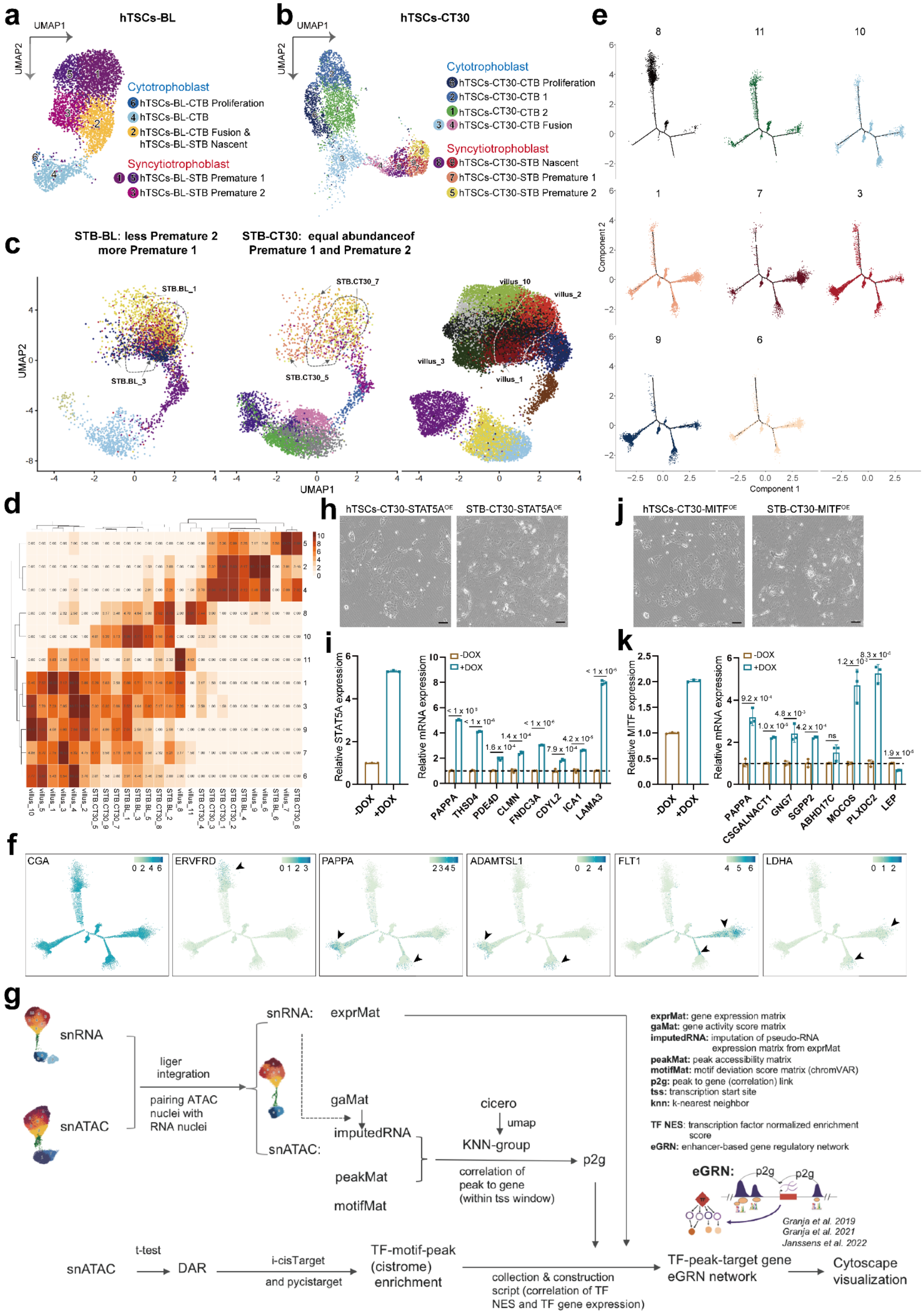
a
hTSCs-BL
UMAP1
UMAP2
Cytotrophoblast
6 hTSCs-BL-CTB Proliferation
4 hTSCs-BL-CTB
2 hTSCs-BL-CTB Fusion & hTSCs-BL-STB Nascent
Syncytiotrophoblast
1 5 hTSCs-BL-STB Premature 1
3 hTSCs-BL-STB Premature 2
b
hTSCs-CT30
Cytotrophoblast
6 hTSCs-CT30-CTB Proliferation
2 hTSCs-CT30-CTB 1
1 hTSCs-CT30-CTB 2
3 4 hTSCs-CT30-CTB Fusion
Syncytiotrophoblast
8 9 hTSCs-CT30-STB Nascent
7 hTSCs-CT30-STB Premature 1
5 hTSCs-CT30-STB Premature 2
c
STB-BL: less Premature 2 more Premature 1
STB-CT30: equal abundanceof Premature 1 and Premature 2
STB.BL_1
STB.BL_3
STB.CT30_7
STB.CT30_5
villus_10
villus_2
villus_3
villus_1
UMAP1
UMAP2
e
8
11
10
1
7
3
9
6
Component 1
Component 2
d
villus_10
villus_5
villus_1
villus_3
villus_4
villus_2
STB.CT30_5
STB.CT30_9
STB.CT30_7
STB.BL_1
STB.BL_3
STB.BL_5
STB.BL_2
STB.CT30_8
villus_8
villus_11
STB.CT30_4
STB.CT30_3
STB.CT30_1
STB.CT30_2
STB.BL_4
villus_9
villus_6
STB.BL_6
villus_7
STB.CT30_6
h
hTSCs-CT30-STAT5A^OE
STB-CT30-STAT5A^OE
i
Relative STAT5A expression
-DOX
+DOX
Relative mRNA expression
< 1 x 10^-5
< 1 x 10^-6
1.6 x 10^-4
1.4 x 10^-4
< 1 x 10^-6
7.9 x 10^-4
4.2 x 10^-5
< 1 x 10^-5
PAPPA
THSD4
PDE4D
CLMN
FNDC3A
CDYL2
ICA1
LAMA3
j
hTSCs-CT30-MITF^OE
STB-CT30-MITF^OE
k
Relative MITF expression
-DOX
+DOX
Relative mRNA expression
9.2 x 10^-4
1.0 x 10^-5
4.8 x 10^-3
4.2 x 10^-4
ns
1.2 x 10^-2
8.3 x 10^-3
1.9 x 10^-5
PAPPA
CSGALNACT1
GNG7
SGPP2
ABHD17C
MOCOS
PLXDC2
LEP
f
CGA
ERVFRD
PAPPA
ADAMTSL1
FLT1
LDHA
g
snRNA
snATAC
liger integration
pairing ATAC nuclei with RNA nuclei
snRNA: exprMat
gaMat
imputedRNA
snATAC:
peakMat
motifMat
cicero
umap
KNN-group
correlation of peak to gene (within tss window)
p2g
exprMat: gene expression matrix
gaMat: gene activity score matrix
imputedRNA: imputation of pseudo-RNA expression matrix from exprMat
peakMat: peak accessibility matrix
motifMat: motif deviation score matrix (chromVAR)
p2g: peak to gene (correlation) link
tss: transcription start site
knn: k-nearest neighbor
TF NES: transcription factor normalized enrichment score
eGRN: enhancer-based gene regulatory network
eGRN:
p2g
Granja et al. 2019
Granja et al. 2021
Janssens et al. 2022
snATAC
t-test
DAR
i-cisTarget and pycistarget
TF-motif-peak (cistrome) enrichment
collection & construction script (correlation of TF NES and TF gene expression)
TF-peak-target gene eGRN network
Cytoscape visualization

Supplementary Figure 6. The comparison of hTSCs and placental organoid with placental villi.

a, b. UMAP shows CTB and STB nuclei profiled with snRNA-seq from STB-BL (a) and STB -CT30 (b).

c. Splitting plots by origin of datasets. Clusters within each dataset are colored separately to clearly show nuclei annotation from predefined STB-BL, STB-CT30 and villus clusters. Overlap of cluster annotations is shown by dotted line and arrows. *In vitro* STB clusters of two STB cell lines co-locate with premature STB clusters *in vivo*, indicating possible incomplete differentiation.

d. Confusion matrix heatmap calculated with predefined clusters in separate sample and integration clusters. Row of matrix: predefined clusters in each sample. Column of matrix: integration cluster. Numbers in heatmap: matching counts. The matching counts are presented with color intensities.

e. Splitting plots of distribution of integration clusters along trajectory paths.

f. Examples of visualization of typical marker genes along trajectory paths. CGA: general STB marker. ERVFRD-1: CTB fusion marker. PAPPA, ADAMTSL1: STB Mature 1 marker. FLT1, LDHA: STB Mature 2 marker. Arrowheads mark the most actively committed state of differentiation by marker gene expression on pseudotime path. The differentiation time is presented with color intensities.

g. Schematic shows the TF-mining and regulatory network construction details in this study.

h. Bright field images show hTSCs-CT30-STAT5A^{OE} and STB-CT30-STAT5A^{OE}. Scale bars: 100 μm. OE, overexpression.

i. RT-qPCR analysis of STAT5A together with its target gene expression in STB-CT30 with DOX-inducible overexpression of STAT5A. Data are shown as mean ± s.d. *P*.values by multiple unpaired two-tailed *t*-test. n = 3 independent experiments. The dotted lines in i and k represent the baseline 1.

j. Bright field images show hTSCs-CT30-MITF^{OE} and STB-CT30-MITF^{OE}. Scale bars: 100 μm.

k. RT-qPCR analysis of MITF together with its target gene expression in STB-CT30 with DOX-inducible overexpression of MITF. Data are shown as mean ± s.d. *P*.values by multiple unpaired two-tailed *t*-test. ns, no significance. n = 3 independent experiments.

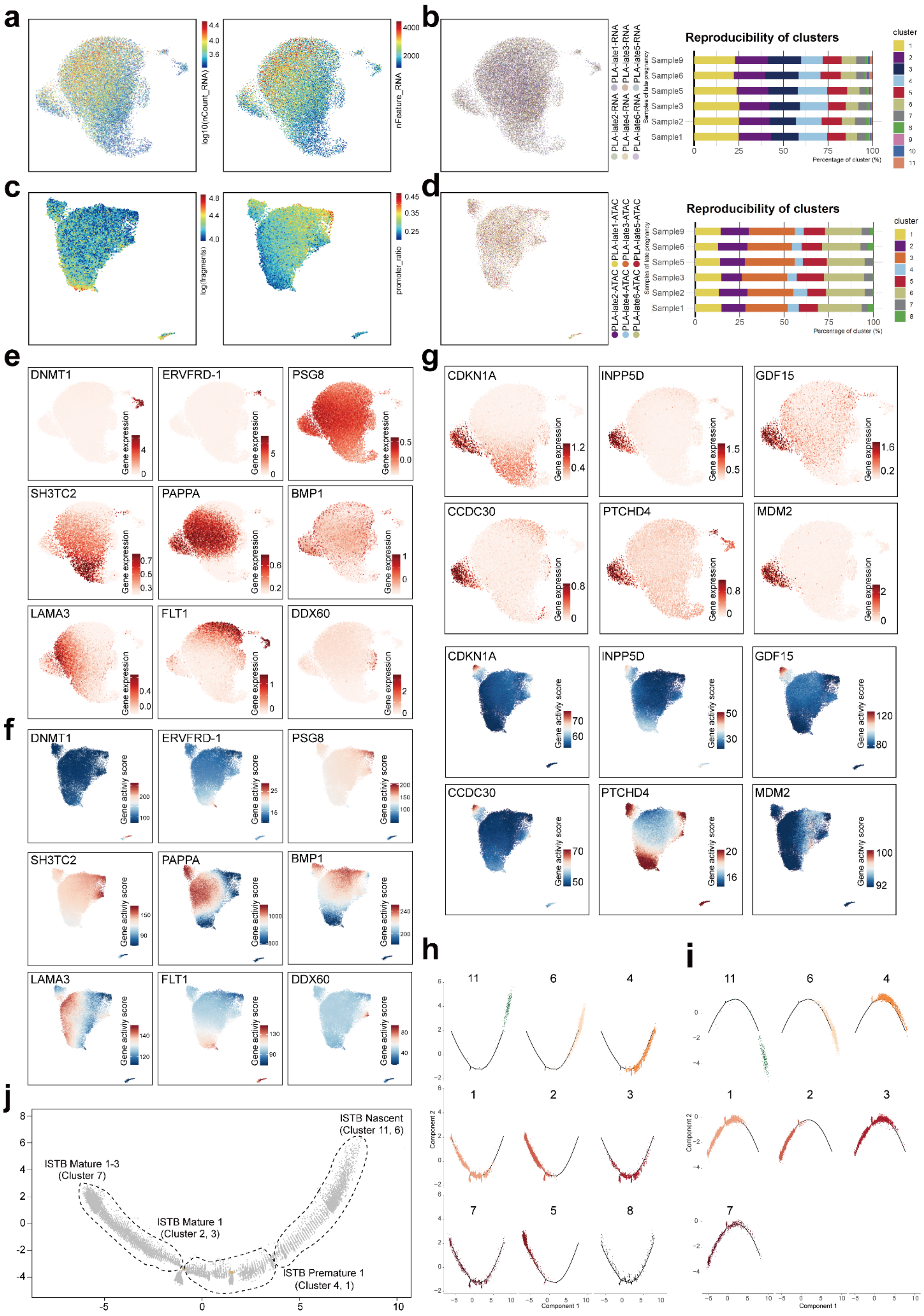
a
log10(nCount_RNA)
nFeature_RNA
b
Reproducibility of clusters
Sample9
Sample6
Sample5
Sample3
Sample2
Sample1
Percentage of cluster (%)
cluster
PLA-late1-RNA
PLA-late2-RNA
PLA-late3-RNA
PLA-late4-RNA
PLA-late5-RNA
PLA-late6-RNA
c
log(fragments)
promoter_ratio
d
Reproducibility of clusters
PLA-late1-ATAC
PLA-late2-ATAC
PLA-late3-ATAC
PLA-late4-ATAC
PLA-late5-ATAC
PLA-late6-ATAC
e
DNMT1
ERVFRD-1
PSG8
SH3TC2
PAPPA
BMP1
LAMA3
FLT1
DDX60
Gene expression
f
Gene activiy score
g
CDKN1A
INPP5D
GDF15
CCDC30
PTCHD4
MDM2
h
i
Component 1
Component 2
j
ISTB Nascent
(Cluster 11, 6)
ISTB Mature 1-3
(Cluster 7)
ISTB Mature 1
(Cluster 2, 3)
ISTB Premature 1
(Cluster 4, 1)

Supplementary Figure 7. Distinct developmental patterns between late pregnancy and early pregnancy based on snRNA-seq and snATAC-seq analyses.

a. UMAP shows the log normalized number of UMI counts (left) and captured genes (right) for snRNA-seq in late pregnancy. The UMI counts and captured genes are presented with color intensities.

b. The distribution of 6 donors on snRNA-seq UMAP and the summary of percentage of each cluster from different donors for the late pregnant placenta.

c. UMAP shows the log normalized fragment counts and promoter ratio (fraction of fragments in promoters) for snATAC-seq in late pregnancy. The fragment counts and promoter ratio are presented with color intensities.

d. The distribution of six donors on snATAC-seq UMAP and the summary of percentage of each cluster from different donors for the late pregnant placenta.

e,f. UAMP shows the specificity of 9 marker gene expression and gene activity score in snRNA-seq and snATAC-seq data. DNMT1: lCTB marker. ERVFRD-1: lCTB Fusion marker. PAPPA: lSTB Mature 1-a, 1-b marker. BMP1: lSTB Premature 1-a, 1-b marker. FLT1: lSTB Mature 2 marker. DDX60: lSTB Mature 2-b marker. All marker genes show consistency between the two data modalities. The expression levels and activity scores are presented with color intensities.

g. UAMP of 6 new marker genes for STB Mature 1-c show potential gene signatures for of this unique cluster of STB nuclei. The expression levels and activity scores are presented with color intensities.

h. i. Distribution of all snATAC-seq clusters along complete trajectory path (h, all lSTB clusters) and lSTB Nascent to lSTB Mature 1 clusters (i, cluster 11,6,4,1,3,2,7).

j. Aggregation of all nuclei clusters along the complete pseudotime trajectory path.

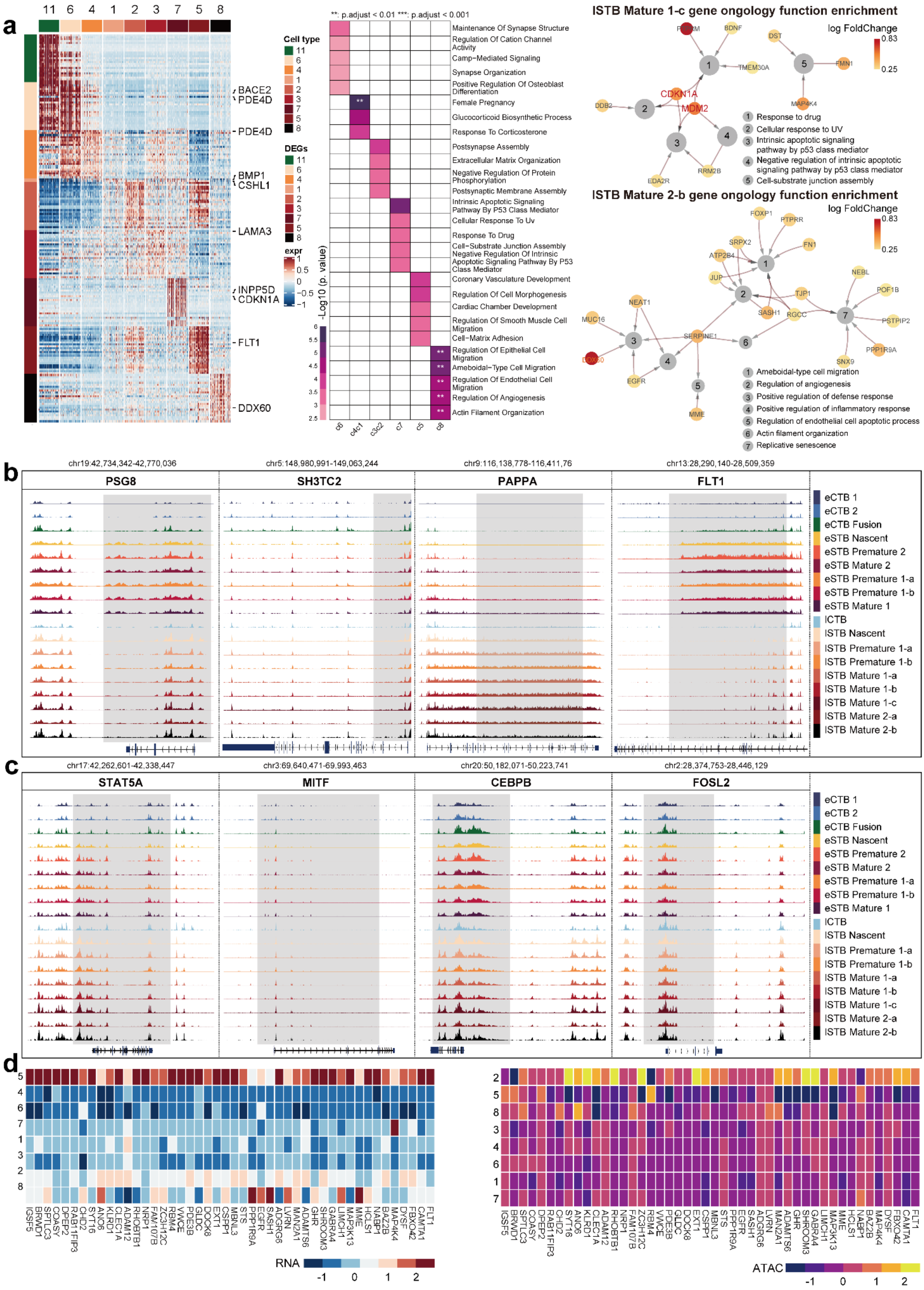
a
Cell type
DEGs
expr
BACE2
PDE4D
PDE4D
BMP1
CSHL1
LAMA3
INPP5D
CDKN1A
FLT1
DDX60
**: p.adjust < 0.01 ***: p.adjust < 0.001
-Log10 (p. value)
Maintenance Of Synapse Structure
Regulation Of Cation Channel Activity
Camp−Mediated Signaling
Synapse Organization
Positive Regulation Of Osteoblast Differentiation
Female Pregnancy
Glucocorticoid Biosynthetic Process
Response To Corticosterone
Postsynapse Assembly
Extracellular Matrix Organization
Negative Regulation Of Protein Phosphorylation
Postsynaptic Membrane Assembly
Intrinsic Apoptotic Signaling Pathway By P53 Class Mediator
Cellular Response To Uv
Response To Drug
Cell−Substrate Junction Assembly
Negative Regulation Of Intrinsic Apoptotic Signaling Pathway By P53 Class Mediator
Coronary Vasculature Development
Regulation Of Cell Morphogenesis
Cardiac Chamber Development
Regulation Of Smooth Muscle Cell Migration
Cell−Matrix Adhesion
Regulation Of Epithelial Cell Migration
Ameboidal−Type Cell Migration
Regulation Of Endothelial Cell Migration
Regulation Of Angiogenesis
Actin Filament Organization
c6
c4c1
c3c2
c7
c5
c8
lSTB Mature 1-c gene ongology function enrichment
log FoldChange
1 Response to drug
2 Cellular response to UV
3 Intrinsic apoptotic signaling pathway by p53 class mediator
4 Negative regulation of intrinsic apoptotic signaling pathway by p53 class mediator
5 Cell-substrate junction assembly
lSTB Mature 2-b gene ongology function enrichment
log FoldChange
1 Ameboidal-type cell migration
2 Regulation of angiogenesis
3 Positive regulation of defense response
4 Positive regulation of inflammatory response
5 Regulation of endothelial cell apoptotic process
6 Actin filament organization
7 Replicative senescence
b
chr19:42,734,342-42,770,036
chr5:148,980,991-149,063,244
chr9:116,138,778-116,411,76
chr13:28,290,140-28,509,359
PSG8
SH3TC2
PAPPA
FLT1
eCTB 1
eCTB 2
eCTB Fusion
eSTB Nascent
eSTB Premature 2
eSTB Mature 2
eSTB Premature 1-a
eSTB Premature 1-b
eSTB Mature 1
lCTB
lSTB Nascent
lSTB Premature 1-a
lSTB Premature 1-b
lSTB Mature 1-a
lSTB Mature 1-b
lSTB Mature 1-c
lSTB Mature 2-a
lSTB Mature 2-b
c
chr17:42,262,601-42,338,447
chr3:69,640,471-69,993,463
chr20:50,182,071-50,223,741
chr2:28,374,753-28,446,129
STAT5A
MITF
CEBPB
FOSL2
d
RNA
ATAC

Supplementary Figure 8. The variation in gene expression, gene openness, and GO function among the STB nuclei in late pregnancy.

a. Gene Ontology of DEGs of each nuclei cluster in late pregnancy (left). The expression level is presented with color intensities. The top 5 terms of GO enrichment reveal the potential STB nuclear functions in late pregnancy (middle, c7: lSTB Mature 1-c, c8: lSTB Mature 2-b). The enrichment is presented with color intensities. Functional networks visualize the GO enrichment results (right). Nodes represent GO term and DEGs while edges represent the signification of enrichment test. Node size: foldchange of DEG. Edge width: *p*.adjust value. The foldchange is presented with color intensities. GO enrichment analysis was tested by one-sided Fisher's exact test and p-values were adjusted for multiple comparison with the Benjamini-Hochberg (BH) method.

b. Gene track plots show local accessible chromatin landscape for 4 marker genes, namely PSG8, SH3TC2, PAPPA, and FLT1.

c. Gene track plots show early and late stage specific chromatin accessibility landscape around master TFs, namely STAT5A, MITF, CEBPB, and FOSL2.

d. Similarity of gene expression and gene activity score (gene openness) for FLT1 and FLT1-like genes in lSTB Mature 2-a (cluster 5, left) in snRNA-seq and lSTB Mature 2-a (cluster 2, right) in snATAC-seq. RNA: snRNA-seq, ATAC: snATAC-seq. The expression level and activity score are presented with color intensities.

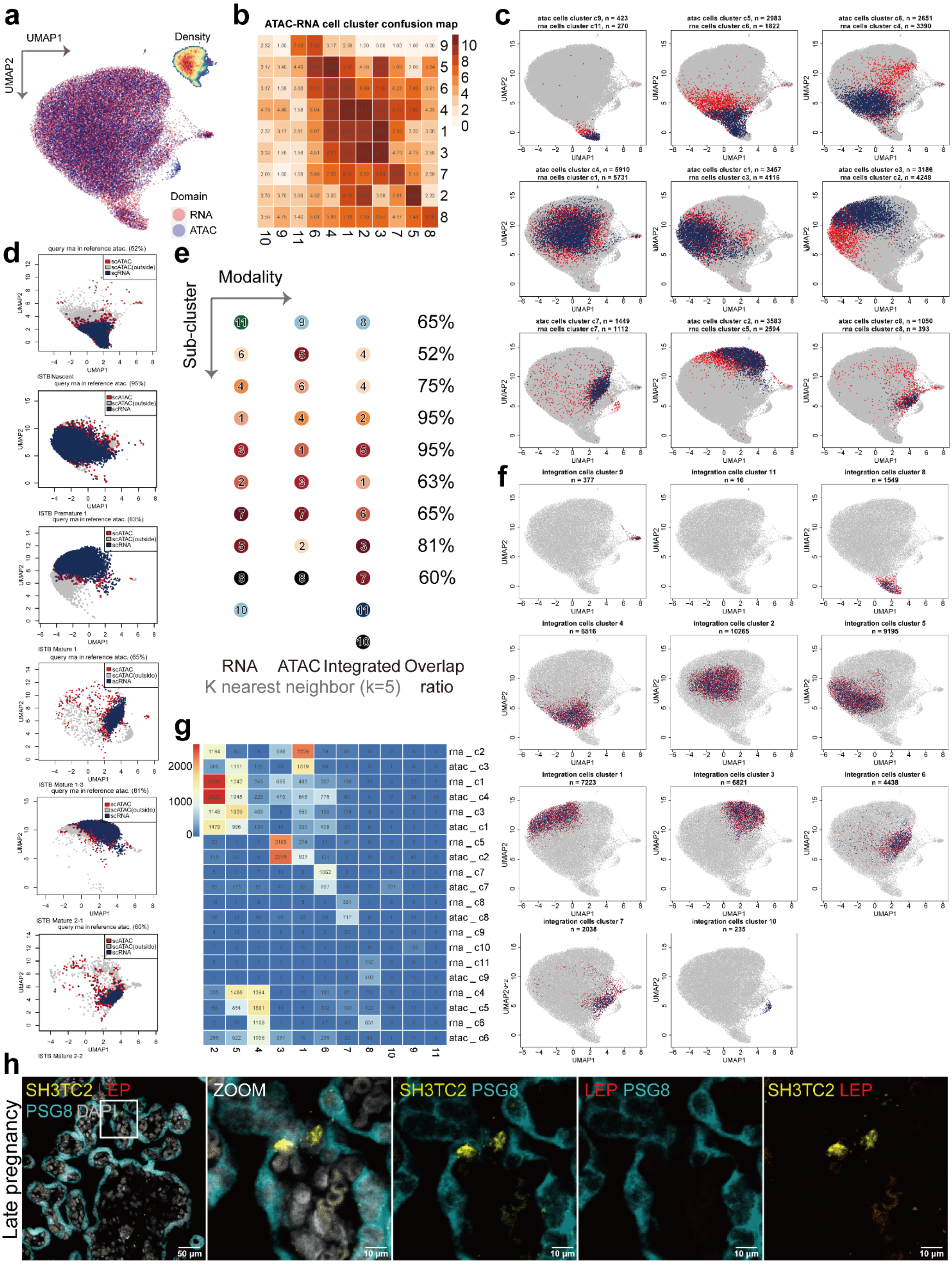

a
b
ATAC-RNA cell cluster confusion map
c
d
e
Modality
Sub-cluster
RNA
ATAC Integrated Overlap
K nearest neighbor (k=5)
ratio
f
g
h
Late pregnancy
SH3TC2 LEP PSG8 DAPI
ZOOM
SH3TC2 PSG8
LEP PSG8
SH3TC2 LEP

Supplementary Figure 9. Quantification of GLUE integration of snRNA-seq nuclei and snATAC-seq nuclei in late pregnancy.

a. The distribution and density (upper right) of nuclei of two data domains (snRNA-seq and snATAC-seq) on UMAP embedding for the late pregnancy. RNA: snRNA-seq, ATAC, snATAC-seq.

b. A confusion map quantifies the matching of snRNA-seq clusters (column) against snATAC-seq clusters (row). The log2 matching count is presented with color intensities.

c. Pairwise cluster distribution on co-embedding UMAP. Dark blue: nuclei in snRNA-seq clusters, Red: nuclei in snATAC-seq clusters.

d. Quantification of matching of nuclei clusters by K nearest neighbor algorithm. rna: snRNA-seq, atac: snATAC-seq. The name of tested nuclei cluster is marked below and the overlap ratio is marked in title.

e. Quantitative summaries of the cluster matching result in the GLUE integration analysis.

f. Distribution of GLUE clusters after integration. Dark blue: nuclei in snRNA-seq nuclei, Red: nuclei in snATAC-seq clusters.

g. Summary table shows GLUE integration cluster composition by annotation with snRNA-seq and snATAC-seq clusters. atac: snATAC-seq, rna: snRNA-seq. The matching count is presented with color intensities.

h. smFISH staining of indicated marker genes (SH3TC2, LEP and PSG8) on human placenta in late pregnancy, indicating the differences among SH3TC2 positive nuclei, PSG8 positive nuclei, and LEP positive nuclei.

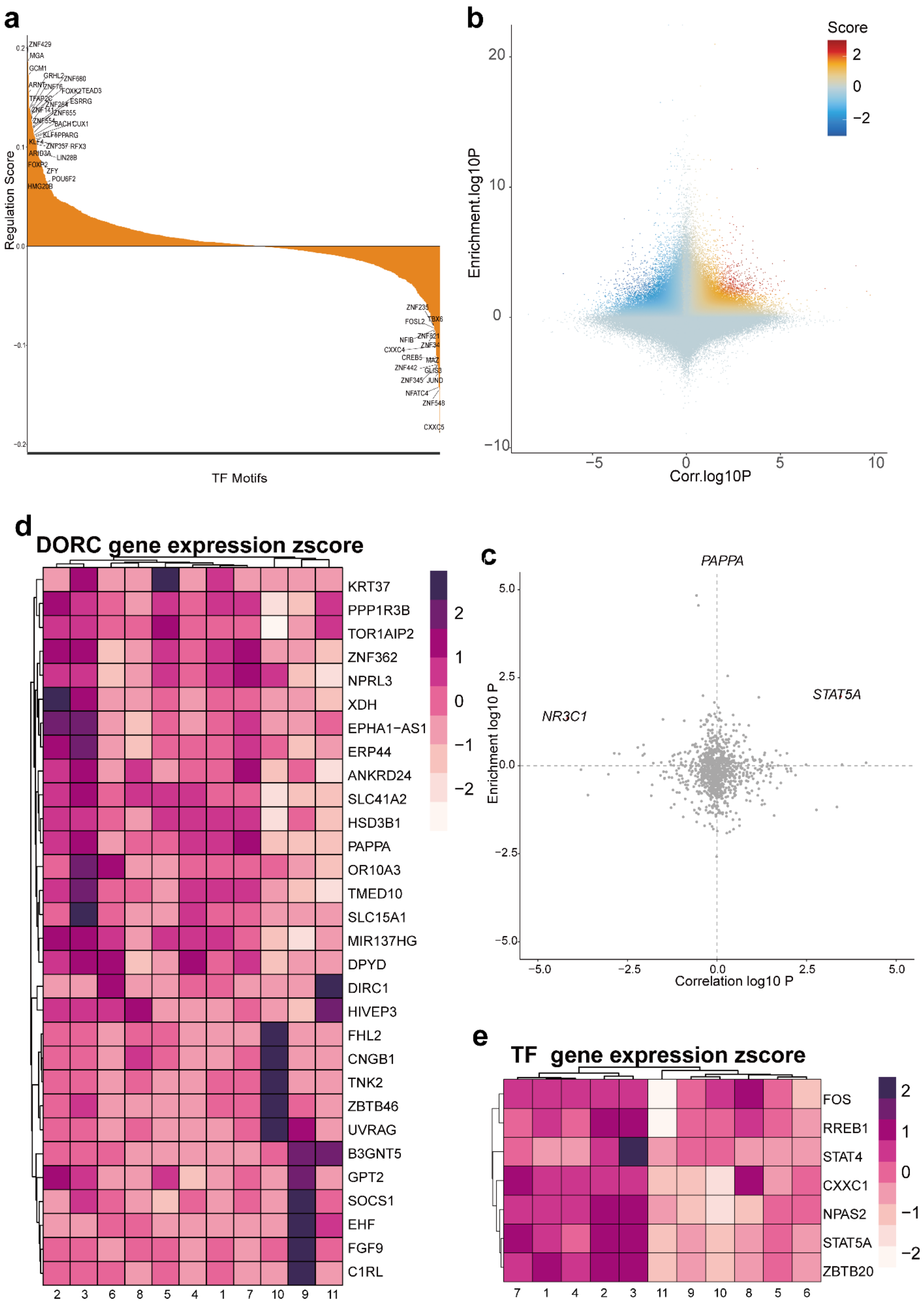
a
Regulation Score
TF Motifs
ZNF429
MGA
GCM1
GRHL2
ZNF680
ARNT
ZNF76
FOXK2
TEAD3
TFAP2C
ZNF284
ESRRG
ZNF655
BACH
CUX1
KLF6
PPARG
KLF4
ZNF357
RFX3
ARID3A
LIN28B
FOXP2
ZFY
POU6F2
HMG20B
ZNF235
FOSL2
TBX6
NFIB
CXXC4
CREB5
MAZ
ZNF442
GLIS3
ZNF345
JUND
NFATC4
ZNF548
CXXC5
b
Score
Enrichment.log10P
Corr.log10P
d
DORC gene expression zscore
KRT37
PPP1R3B
TOR1AIP2
ZNF362
NPRL3
XDH
EPHA1−AS1
ERP44
ANKRD24
SLC41A2
HSD3B1
PAPPA
OR10A3
TMED10
SLC15A1
MIR137HG
DPYD
DIRC1
HIVEP3
FHL2
CNGB1
TNK2
ZBTB46
UVRAG
B3GNT5
GPT2
SOCS1
EHF
FGF9
C1RL
2 3 6 8 5 4 1 7 10 9 11
c
PAPPA
NR3C1
STAT5A
Enrichment log10 P
Correlation log10 P
e
TF gene expression zscore
FOS
RREB1
STAT4
CXXC1
NPAS2
STAT5A
ZBTB20
7 1 4 2 3 11 9 10 8 5 6

Supplementary Figure 10. Systematic identification of TFs along the STB differentiation process.

a. Candidate TF genes are ranked by regulation score (both positive and negative). The top 25 TF genes are marked by gene name.

b. Selection of TF regulators by combination of enrichment score with correlation co-efficiency between upstream regulatory cis-element accessibility of target genes and TF gene expression. A regulatory score is calculated based on these two dimensions and displayed as colored dots. Hot color dots represent positive TF activators and cold color ones represent negative TF repressors. The x-axis p-values were produced by correlation test with the spearman method. The y-axis p-values were produced by one-tailed Z-test.

c. An example scatterplot shows TF activators (STAT5A) and TF repressors (NR3C1) of target gene PAPPA in late pregnancy. Statistic methods used in the x-axis and y-axis were the same as b.

d,e. Heatmaps of lSTB Matrue 1-a, 1-b regulatory show the nuclei cluster expression consistency between target (DORC) genes (d) and TF regulators (e). Most of genes show a relatively high expression profile in cluster 3 (lSTB Matrue 1-a) and cluster 2 (lSTB Matrue 1-b). The expression zscores are presented with color intensities. Related to Figure 5i.

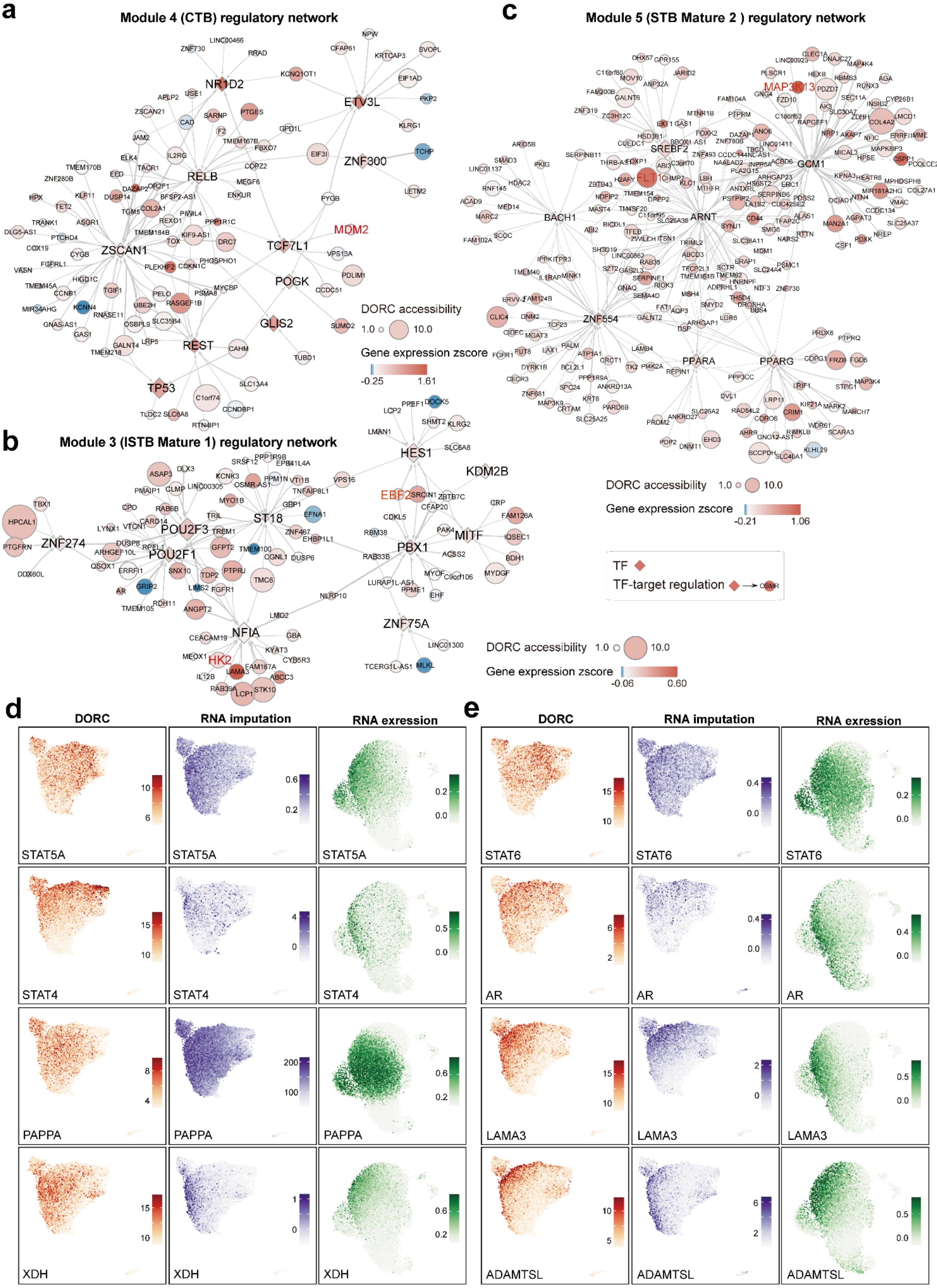
a
Module 4 (CTB) regulatory network
NR1D2
ETV3L
ZNF300
RELB
ZSCAN1
TCF7L1
MDM2
POGK
GLIS2
REST
TP53
DORC accessibility
1.0
10.0
Gene expression zscore
-0.25
1.61
b
Module 3 (ISTB Mature 1) regulatory network
HES1
KDM2B
EBF2
ZNF274
POU2F3
ST18
POU2F1
PBX1
MITF
NFIA
HK2
ZNF75A
DORC accessibility
1.0
10.0
Gene expression zscore
-0.06
0.60
c
Module 5 (STB Mature 2) regulatory network
MAP3K13
SREBF2
GCM1
FLT1
BACH1
ARNT
ZNF554
PPARA
PPARG
DORC accessibility
1.0
10.0
Gene expression zscore
-0.21
1.06
TF
TF-target regulation
OSMR
d
DORC
RNA imputation
RNA exression
STAT5A
STAT4
PAPPA
XDH
e
DORC
RNA imputation
RNA exression
STAT6
AR
LAMA3
ADAMTSL

Supplementary Figure 11. TF-target regulatory modules identify intrinsic TF regulators governing the STB nuclei differentiation in late pregnancy.

a, b, c. Networks show three transcription factor-target gene regulatory modules for lSTB Mature1 (a), lSTB Mature 2 (b), and CTB (c) with typical TFs. The DORC accessibility and gene expression score are presented with circle sizes and color intensities, respectively. Related to Figure 5h.

d, e. Comparison of TF genes and their target genes openness and expression in module 1 (d) and module 2 (e) regulatory network. Target genes are highlighted in red in Figure 5i. The domain of regulation chromatin score (DORC), RNA imputation score and RNA expression level are presented with color intensities.

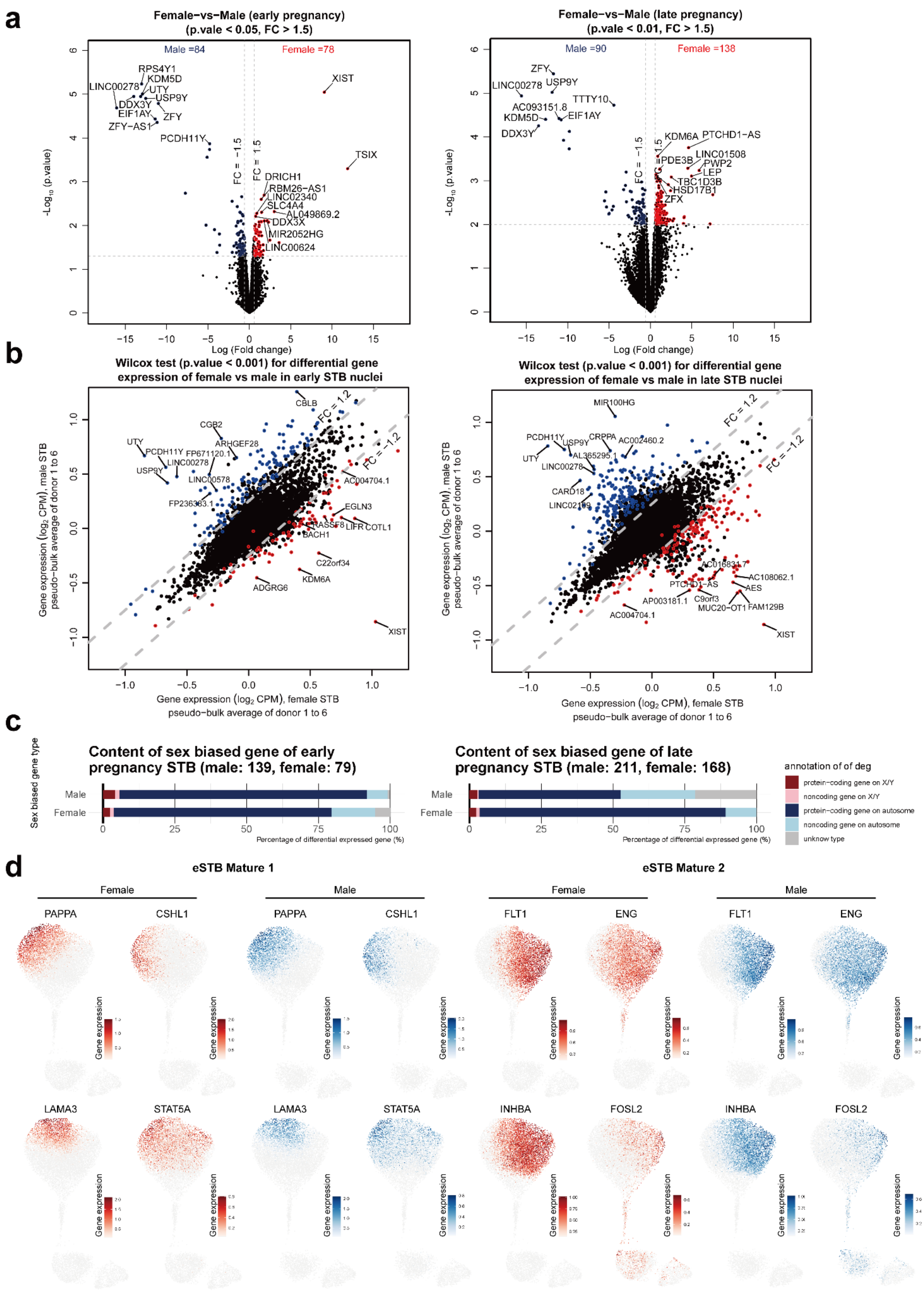
a
Female−vs−Male (early pregnancy)
(p.vale < 0.05, FC > 1.5)
Male =84
Female =78
XIST
TSIX
RPS4Y1
KDM5D
LINC00278
UTY
USP9Y
DDX3Y
EIF1AY
ZFY
ZFY−AS1
PCDH11Y
DRICH1
RBM26−AS1
LINC02340
SLC4A4
AL049869.2
DDX3X
MIR2052HG
LINC00624
FC = −1.5
FC = 1.5
-Log10 (p.value)
Log (Fold change)
Female−vs−Male (late pregnancy)
(p.vale < 0.01, FC > 1.5)
Male =90
Female =138
ZFY
LINC00278
USP9Y
TTTY10
AC093151.8
KDM5D
EIF1AY
DDX3Y
KDM6A
PTCHD1−AS
LINC01508
PDE3B
PWP2
LEP
TBC1D3B
HSD17B1
ZFX
b
Wilcox test (p.value < 0.001) for differential gene expression of female vs male in early STB nuclei
CBLB
CGB2
ARHGEF28
UTY
PCDH11Y
FP671120.1
LINC00278
USP9Y
LINC00578
FP236383.1
AC004704.1
EGLN3
RASSF8
LIFR
COTL1
BACH1
C22orf34
KDM6A
ADGRG6
XIST
FC = 1.2
FC = −1.2
Gene expression (log2 CPM), male STB pseudo−bulk average of donor 1 to 6
Gene expression (log2 CPM), female STB pseudo−bulk average of donor 1 to 6
Wilcox test (p.value < 0.001) for differential gene expression of female vs male in late STB nuclei
MIR100HG
PCDH11Y
USP9Y
CRPPA
AC002460.2
UTY
AL365295.1
LINC00278
CARD18
LINC02109
AC016831.7
AC108062.1
PTCHD1−AS
AES
AP003181.1
C9orf3
MUC20−OT1
FAM129B
AC004704.1
XIST
c
Sex biased gene type
Content of sex biased gene of early pregnancy STB (male: 139, female: 79)
Content of sex biased gene of late pregnancy STB (male: 211, female: 168)
Male
Female
Percentage of differential expressed gene (%)
annotation of of deg
protein−coding gene on X/Y
noncoding gene on X/Y
protein−coding gene on autosome
noncoding gene on autosome
unknow type
d
eSTB Mature 1
eSTB Mature 2
Female
Male
PAPPA
CSHL1
FLT1
ENG
LAMA3
STAT5A
INHBA
FOSL2
Gene expression

Supplementary Figure 12. Sex-biased gene expression in the placenta did not affect the classification of STB nuclear subclusters.

a. Volcano plots show differentially expressed sex-specific genes in early (left) and late (right) pregnancy. snRNA-seq was aggregated as pseudo-bulk RNA-seq data to identify sex-specific gene expression by the one-sided glmQLFTest (FC >= 1.5) function in R package edgeR.

b. Scatter plots show differentially expressed sex-specific genes in the STB nuclei in early (left) and late (right) pregnancy. Differentially expressed genes were identified by two-sided wilcox-test (FC >= 1.2) with the Seurat FindAllMarkers function.

c. Annotation of sex-specific genes in the STB nuclei in early (left) and late (right) pregnancy.

d. UMAP embeddings show differentially expressed genes and TFs in eSTB Mature 1 (left) and eSTB Mature 2 (right) of female and male donors during early pregnancy. The expression levels are presented with color intensities.

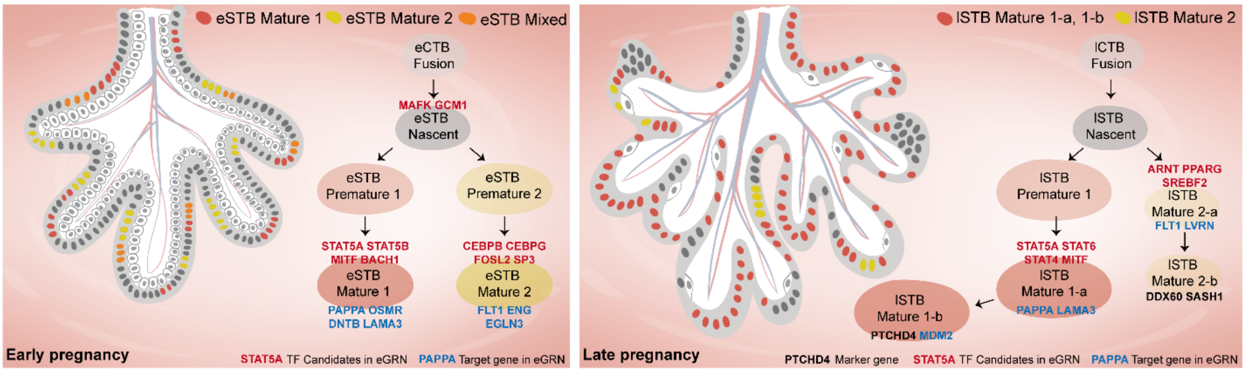

Supplementary Figure 13. Diagram illustrating the newly identified subclusters of STB cells, their proposed differentiation pathway, and the lineage-specific transcription factors (TFs).

Supplementary Note

Supplementary Note 1

To avoid potential sex issues that may influence our main conclusions, we collected an equal number of placentas on each gender confirmed by PCR on gender-specific marker gene SRY and allele for sex chromosomes like NLGN4, before we prepare single nuclei for snRNA-seq and snATAC-seq (Supplementary Fig. 1a and1c, and Supplementary Table 1). Sex-biased gene expression has been reported in various human organ systems, such as the muscle, skeleton, thyroid, and stomach [1]. Moreover, diseases such as cancers and autoimmune disorders have also been documented with sex-specific characteristics [2,3]. To mitigate potential sex bias in our findings, we acquired an equal number of male and female placentas. We identified a total of 372 genes (162 in early pregnancy, 228 in late pregnancy, 18 shared) that showed sex-biased gene expression, with the majority being coding genes located on autosomes. Male and female STB nuclei distributed on UMAP revealed an even distribution of the most notable DEGs, such as PAPPA, CSHL1, LAMA3, and STAT5A in eSTB Mature1, as well as FLT1, ENG, INHBA, and FOSL2 in eSTB Mature2, confirming that our primary conclusions on STB nuclear heterogeneity were unaffected by sex-biased factors (Supplementary Fig. 12). Nonetheless, future studies investigating sex-biased genes in the placenta will provide invaluable insights into the sex-related features and underlying mechanisms of this organ.

Immunohistochemical staining for KRT7 confirmed the typical villous architecture of the placentas (Supplementary Fig. S1a). Equal portions of intact placental nuclei from the same placenta were used for snRNA-seq and snATAC-seq to mitigate differences in nuclear composition and facilitate integrated downstream analysis.

Supplementary Note 2

We annotated seven major nuclear clusters including eCTB (KRT7, cytotrophoblast nuclei at early pregnancy) (early pregnancy hereinafter referred as e), lCTB (KRT7, cytotrophoblast nuclei at late pregnancy) (late pregnancy hereinafter referred as l), eSTB (PSG5, PSG8, syncytiotrophoblast nuclei), lSTB (PSG5, PSG8, syncytiotrophoblast nuclei), eEVT (HLA-G, extravillous-trophoblast nuclei), eSTR (VIM, PECAM1, CD4, DLK1, stromal nuclei), and lSTR (VIM, PECAM1, CD14, DLK1 stromal nuclei). Only a portion of CTBs remained proliferative (Supplementary Fig. 1h), which is consistent with our previous study[4].

Supplementary Note 3

Whether the STB nuclei have transcriptional ability has been controversial, to directly address this, we performed RNA velocity analysis base on our snRNA-seq

datasets. We systematically compared the spliced and un-spliced RNA ratio among all the identified cell types. Our results showed that the ratio of spliced and un-spliced RNA are relatively the same among those cell types, indicating that nuclei in the STB have the same transcriptional capabilities with other cell types in the placenta (Supplementary Fig. 1b).

Supplementary Note 4

To explore gene expression dynamics in STB nuclei during early and late pregnancy, we employed three statistic methods including the Wilcox test to identify the differentially expressed genes (DEGs). The top 20 upregulated genes in STB from different pregnancy stages were shown in Fig. 1d and Supplementary Table 2. The expression of FLT1, ENG, LEP, and SASH1 were significantly higher in STB at early pregnancy than in STB at late pregnancy, while the expression of PAPPA, CSH1, SVEP1, and CSHL1 were higher in STB at late pregnancy than in STB at early pregnancy (Fig. 1d). The UMAP plot in Supplementary Fig. 1i illustrated distinct expression patterns of DEGs between these two pregnancy stages. GO analysis of the DEGs revealed functional differences between early and late pregnancy in STB (Fig. 1e).

Supplementary Note 5

Confirmation of nuclear subcluster identities was based on gene activity score and gene expression of canonical marker genes (Supplementary Fig. 2c). Specifically, we found that STB nuclei with specifically high expression of PAPPA were significantly overrepresented at late pregnancy while STB nuclei with specifically high expression of FLT1 were significantly decreased in proportion at late pregnancy (Supplementary Fig. 2d). Moreover, the co-existence of FLT1 positive and PAPPA positive nuclei in early STB raised the possibility of heterogeneity within STB nuclear populations (Supplementary Fig. 2d).

Supplementary Note 6

We used single-molecule fluorescence in situ hybridization (smFISH) to validate the existence of STB nuclear subtypes at early pregnancy and to determine their location in the placental tissue with probes designed to detect the RNA transcripts of SH3TC2, LEP, PSG8, PAPPA, and FLT1 (Fig. 2c and Supplementary Fig. 3h). Our observations revealed the separation of different nuclear subtypes within the STB, with probes for SH3TC2 (eSTB Nascent), LEP (eSTB at pre-matured and mature state), and PAPPA (eSTB Mature 1), FLT1 (eSTB Mature 2), and PSG8 (pan-STB subtypes) (Supplementary Fig. 3h). To confirm the even distribution of FLT1 and PAPPA positive STB nuclei in placental villi at early pregnancy as predicted by our analysis (Fig. 2a),

we calculated the percentage of these nuclei using smFISH (Fig. 2c). Our bioinformatic anticipation was confirmed by the exclusive expression of FLT1 and PAPPA with even distribution in distinct STB nuclei (Supplementary Fig. 3h).

Supplementary Note 7

To explore potential heterogeneity among STB nuclei during differentiation, we performed pseudotime trajectory analysis of snRNA-seq data to delineate STB nuclear subtype along the differentiation trajectories. We observed two major developmental trajectory of STB nuclei: from CTB Fusion (cluster 11) to eSTB Nascent (cluster 8), and ultimately towards mature states with high expression of either PAPPA (hereinafter reffered as eSTB Mature 1, cluster 10) or FLT1 (hereinafter reffered as eSTB Mature 2, cluster 3) (Fig. 2d, Supplementary Fig. 3c). To further investigate the global dynamic changes in accessibility patterns during STB nuclear differentiation, we inferred differentiation pseudotime from snATAC-seq data using a supervised method described in Granja et al[5]. The generated trajectory also revealed two branches, both emerging from a common progenitor populations, which we have named eSTB Nascent (hereinafter referred as eSTB Nasent), and leading towards the tips of the two branches, and we have named these, eSTB Mature 1 and eSTB Mature 2 (Fig. 2e).

Supplementary Note 8

To determine whether the diverse STB nuclear populations reflected varied functional consequences, we sought to identify biological pathways that were enriched among the DEGs using GO enrichment analysis. (Fig. 2f and 2g, Supplementary Fig. 3d, 3f, and 3e; Supplementary Table 4). Genes related to the EGFR and ERBB pathways were upregulated in the eSTB Nascent subtype (Supplementary Fig. 3e). The upregulated DEGs for eSTB Mature 1 were associated with the growth hormone receptor signaling pathway through the JAK-STAT pathway. For eSTB Mature 2, the upregulated DEGs were coalesced in response to oxygen levels, response to hypoxia, lipid localization, and transportation of monocarboxylic acid, and carboxylic acid (Fig. 2f and 2g). In addition, we found that polypeptide hormone genes enriched in eSTB Mature 1 were chorionic somatomammotropin CSH1/2, CSHL1, and GH2 et al, in contrast, hormone genes enriched in eSTB Mature 2 were ANG, LEP, FSTL3 et al. The unique expression patterns of these polypeptide hormone genes may indicate the functional heterogeneity of the STB nuclear subtypes (Supplementary Fig. 3g).

Supplementary Note 9

These subtypes identified in the integrated UMAP include the eCTB 1, eCTB 2, eCTB Fusion, eSTB Nascent, eSTB Premature1, eSTB Mature 1, eSTB Premature 2,

eSTB Mature 2, and eSTB Mixed. The gene expression levels of ERVFRD-1, PSG8, SH3TC2, LEP, FLT1 and PAPPA were consistent with their gene activity scores in the snATAC-seq datasets (Fig. 3a and Supplementary Fig. 5a). We calculated the overlap ratios of nuclei annotated in either snRNA-seq or snATAC-seq to the corresponding clusters on the integrated UMAP plot. Our results demonstrated an overlap ratio of 70%, 73%, and 80% for STB nascent, eSTB Mature1 and eSTB Mature 2, respectively (Supplementary Fig. 5b).

Supplementary Note 10

Along the delineated differentiation trajectory at early pregnancy, eCTB1 and eCTB Fusion demonstrated accessibility at *cis*-regulatory elements neighboring transcription start sites (TSSs) of cytotrophoblast markers such as TP73, DNMT1, and CDH1 (Fig. 3b, left). We identified accessible cCREs neighboring the TSSs of TFs OVOL1 and GCM1 et al. in eCTB Fusion. Furthermore, we identified accessible cCREs neighboring the TSSs of newly identified TFs such as ESRRG. This result suggested their specific roles for them in orchestrating identity allocations from fusion competent CTB nuclei into nascent STB nuclei (Fig. 3b and Supplementary Fig. 4). Moreover, we observed chromatin accessibility at cCREs neighboring the newly identified STB subcluster markers such as SH3TC2 for eSTB Nascent, PAPPA for eSTB Premature 1 and eSTB Mature 1, and FLT1 for eSTB Premature 2 and eSTB Mature 2 (Fig. 3b). Additionally, we observed comparable chromatin accessibility patterns in several genes, including CSHL1, GH2, AR, NPAS2, STAT4, STAT5A, STAT5B, and LEP with PAPPA (representative of eSTB Mature 1), and ENG, HIF1A, MYCN, LVRN, POU2F2, FOSL2, BACH2, and CEBPB with FLT1 (representative of eSTB Mature 2), indicating their possible roles in determining distinct features of STB nuclear heterogeneity (Supplementary Fig. 4). Upon scrutinizing genome tracks, accessible patterns of cCREs in SH3TC2, FLT1, PAPPA, ESRRG, OVOL2, STAT5A, HIF1A, and CEBPB along the STB nuclear differentiation path were revealed (Fig. 3b right and Supplementary Fig. 4). ESRRG was known to play a pivotal role in facilitating trophoblast differentiation and invasion, as well as pancreatic beta cell maturation[6]. Interestingly, ESRRG-regulated genes such as HSD11B2 and KCNQ1, showed similar chromatin accessibility with ESRRG in our dataset (Supplementary Fig. 4).

Supplementary Note 11

To explore the potential master-determining TFs in regulating differentiation of STB nuclei into two trajectories, we employed a multiomic-based strategy for TF enrichment in specific STB nuclear subclusters [6]. In total, 20 TFs were eligible and showed a strong positive correlation, suggesting that they open chromatin towards CTB to STB differentiation. The enriched motifs in eCTB 1 and eCTB 2 included TFs like TP63,

TEAD4, E2F3, TP53, RELA, GRHL3, ZNF217, and FOSL1 (Fig. 3c). The enriched motifs in eCTB Fusion included TFs like GCM1, MAFK, and MAFG. We found that the motifs for CEBPB, CEBPG, FOSL2, STAT4, STAT5A, MITF, BACH1, ZBTB21, and NFE2 et al. were enriched in STB. It is worth noting that the differentiation trajectory towards PAPPA highly expressed nuclei (eSTB Mature 1) showed the top enrichment for motifs of STAT5B, STAT5A, MITF, and STAT4, meanwhile the differentiation trajectory towards FLT1 highly expressed nuclei (eSTB Mature 2) had the top enrichment for CEBPB, CEBPG, FOSL2, and SP3 motifs, indicating these TFs may be potentially implicated in the regulation of these two differentiation trajectories of STB nuclei and can be considered as potential master regulators (Fig. 3c).

Supplementary Note 12

We quantified the distribution of canonical CTB markers such as TP63, TEAD4, ITGA6, and GATA3 in both cell lines using qPCR. hESCs-RUES2 (hereinafter referred as hESCs) served as controls. Compared to hESCs, both hTSCs-BL and hTSCs-CT30 strongly expressed trophoblast markers (Fig. 3f and 3g). Additionally, scRNA-seq analysis revealed similar features between the cellular subtypes of hTSCs-BL and hTSCs-CT30 in vitro (Supplementary Fig. 5f and 5g).

Supplementary Note 13

After stringent quality control (Supplementary Fig. 5h, 5i, and 5j), UMAP plot revealed six subclusters in data generated from hTSCs-BL and STB-BL, and nine subclusters from hTSCs-CT30 and STB-CT30. We used canonical trophoblast markers and specifically expressed genes, that we used to annotate the placental STB nuclear subclusters *in vivo,* to annotate the newly generated nuclear/cellular subclusters *in vitro* (Supplementary Fig. 6a and 6b). The CTB clusters were marked by expression of genes like CDH1 and the STB cluster was marked by expression of CGA and LEP (Fig. 3g). We integrated *in vitro* and *in vivo* snRNA-seq data from placentas to assess whether our *in vitro* trophoblast models (hTSCs-BL, hTSCs-CT30, STB-BL, and STB-CT30) could mimic the CTB subtypes and STB nuclear subclusters we identified in this study. The DEG heatmap of STB-BL, STB-CT30, and STB-villus demonstrated a range of marker gene expression, including some key master TFs, comparable to *in vivo* STB (Fig. 3i).

Supplementary Note 14

We next aimed to determine whether there is a correspondence regulatory mechanisms exist between *in vitro* and *in vivo* STB. To achieve this, we overexpressed STAT5A and MITF in both hTSCs-BL and hTSCs-CT30 using full-length CDS with a Flag-tag since their expression levels were low in *in vitro* STB relative to their *in vivo*

counterparts identified in the eSTB Mature 1 (Fig. 4a, 4b, 4c, 4d, and 4e, Supplementary Table 7). We differentiated hTSC-BL-STAT5A^{OE} into STB. Both hTSC-BL-STAT5A^{OE} and STB-BL-STAT5A^{OE} retained their normal cell morphology. Quantitative real time PCR (qRT-PCR) showed a significant increase of STAT5A expression (Fig. 4c). We identified a significant increase in the gene expression of potential targets such as PAPPA, THSD4, PDE4D, CLMN, FNDC3A, CDYL2, ICA1, and LAMA3 (Fig. 4c). Using the same strategy for MITF, we identified a significant increase in the gene expression of potential targets for MITF such as PAPPA, CSGALNACT1, GNG7, SGPP2, ABHD17C, MOCOS, PLXDC2, and LEP (Fig. 4e). This result further confirm our eGRN as shown in Fig. 3d. Similar results were obtained for hTSC-CT30 (Supplementary Fig. 6h, 6i, 6j, and 6k).

The expression of master TFs CEBPB and FOSL2, identified in the eSTB Mature 2 subcluster, was higher in STB-BL than that in the early pregnancy placenta. To assess the overlap between their downstream peaks of targets in the eGRN from *in vivo* datasets and the CUT&Tag peaks detected in STB-BL, we performed ChIP-seq and/or CUT&Tag experiments for CEBPB and FOSL2. Among the 428 CEBPB target gene peaks assigned in the eSTB Mature 2 eGRN, 248 (57.9%) overlapped with CEBPB CUT&Tag peaks in STB-BL. We performed FOSL2 CUT&Tag, and the peaks were identified using the same method as that used for CEBPB. FOSL2 peaks overlapped with target peaks by 74.6%. Additionally, we conducted a ChIP-seq experiment for CEBPB in STB-BL, with hTSC-BL as a negative control. The results confirmed STB-specific peak signals in STB-BL. This confirmed the consistent regulatory events between the eGRN in placental STB at early pregnancy and STB differentiated from hTSCs. Genomic tracks of shared target genes, including FLT1, ENG, EGLN3, HK2, INHBA, and NDRG1, were visualized (Fig. 4f). In our previous findings (Fig. 3d), we identified MITF as a possible master regulator of hormone genes, including CSH2 and LEP. To test this in *in vitro* models, we investigated the role of MITF in the developing trophoblast organoids constructed from hTSCs-BL. We generated trophoblast organoids using hTSCs-BL with DOX-inducible overexpression of MITF. ELISA assays showed a positive correlation between CSH2 secretion and the overexpression of MITF in STB in the trophoblast organoids (Fig. 4g, 4h, and 4i).

Supplementary Note 15

To ensure the robustness of our analysis, we applied stringent quality control and batch effect correction analysis (Supplementary Fig. 7a, 7b, 7c, and 7d), we annotated CTB nuclear cluster and STB nuclear subclusters in both datasets using gene expression and gene activity scores of known markers (Fig. 5a and 5b, Supplementary Fig. 7e, and 7f). For snRNA-seq datasets, we used known markers to further annotate these subclusters including ICTB (clusters 10 and 9, DNMT1, and CTB nuclei), and

lSTB (clusters 11, 6, 4, 1, 3, 2, 7, 5, and 8, PSG8, and STB nuclei, Supplementary Table 3). We observed limited expression of ERVFRD-1 in the CTB cluster (cluster 9, lCTB Fusion), indicating a decreased fusion capability of CTBs at late pregnancy (Fig. 5a and Supplementary Fig. 7e).

Supplementary Note 16

Interestingly, within the PAPPA positive population, we observed biased expression of gene encoding BMP1 and CSH2 in cluster 4 and cluster 1, which we annotated as lSTB Premature 1-a and lSTB Premature 1-b, and LAMA3 in cluster 3 and cluster 2, which we annotated as lSTB Mature 1-a and lSTB Mature 1-b (Supplementary Fig. 7e, Supplementary Table 8). The GO analysis indicated the up-regulation of genes in cluster 4 and 1 were related to “female pregnancy,” “glucocorticoid biosynthetic process,” and “response to corticosterone” (Supplementary Fig. 8a). Cluster 7, characterized by PTCHD4 expression, was a late-pregnancy-specific STB nuclear subcluster that specifically expressed CDKN1A, INPP5D, GDF15, CCDC30, and MDM2 (Supplementary Fig. 7g). PTCHD4 has been previously associated with p53 responsive pathways, and MDM2 targets p53 for proteasome degradation in response to DNA damage [7,8]. We thus annotated cluster 7 as lSTB Mature 1-c. High expression of MDM2 and PTCHD4 indicated a potential DNA damage response in the nuclei within Cluster 7 (Fig. 5a and Supplementary Fig. 7g). The identity and function of cluster 7 were predicted using GO enrichment analysis. The major biological processes that were enriched in cluster 7 were related to the “intrinsic apoptotic signaling pathway by p53 class mediator,” “cellular response to UV,” and “cell-substrate junction assembly negative regulation of intrinsic apoptotic signaling pathway by p53 class mediator”, reminiscent of the features of syncytial knot[9,10] and confirmed the key roles of nuclei in cluster 7 in response to DNA damage (Supplementary Fig. 8a), since continuous DNA damage and senescence was reported to impair embryonic development, future study focused on STB nuclei in cluster 7 in protecting embryonic development through DNA damage response will be interesting[11,12]. Cluster 8, characterized specifically by DDX60 and DDX58 expression, represents late-pregnancy-specific STB nuclear subclusters that we annotated as lSTB Mature 2-b (Fig. 5a and Supplementary Fig. 7e). DDX60 and DDX58 are interferon-stimulated genes that are involved in viral RNA degradation and extracellular vesicle formation [13]. DDX60 is also associated with several cancer [14]. GO analysis further revealed that the major terms enriched in cluster 8 were associated with collagen fibril organization and regulation of mRNA stability (Supplementary Fig. 8a). The high expression of DDX60 might be involved in additive protection against antiviral effects in the STB at late pregnancy[15].

Supplementary Note 17

The placenta villi at late pregnancy were stained with probes targeting PSG8, SH3TC2, LEP, PAPPA and FLT1. Compared with the results for the placenta villi at early pregnancy (Fig. 2e), we found that only a small number of nuclei were positive for SH3TC2, and fewer nuclei positive for LEP were present (Supplementary Fig. 9h). For the smFISH of FLT1 and PAPPA, we found that few if any FLT1-positive nuclei in the STB at late pregnancy, whereas most of the STB nuclei were positive for PAPPA (Fig. 5f). These findings were consistent with our bioinformatics analysis and suggest that PAPPA differentiation becomes the dominant feature during late-stage pregnancy.

Supplementary Note 18

Next, we sought to determine key genomic regions crucial for STB nuclear gene expression and differentiation. To achieve this goal, we employed an iterative approach for snATAC-seq data to obtain a low-dimensional embedding and cell clusters on 24,692 nuclei. The quality of these clusters was evaluated using the number of fragment counts and the ratio of reads located in gene promoter regions (Supplementary Fig. 7c and 7d). Notably the structure of the chromatin and the RNA expression representation were similar. These nuclei for snATAC-seq could be further clustered into 9 clusters based on calculated gene activity scores (Fig. 5b, Supplementary Fig. 7f). We used known markers to further annotate these subclusters including lCTB (cluster 9, DNMT1, cytotrophoblast nuclei), and lSTB (Clusters 5, 6, 4, 1, 3, 7, 2, and 8, PSG8, syncytiotrophoblast nuclei). Among them, cluster 5 was annotated as nascent STB nuclei due to high gene activity scores of SH3TC2 and PSG8 (Fig. 5b). Notably, the gene activity score of PAPPA also dominated in the snATAC-seq datasets (clusters 6, 4, 1, and 3, PAPPA, Supplementary Fig. 7f). Cluster 6 and cluster 4 were identified as two kinds of premature STB nuclei at late pregnancy with less PAPPA expression, were thus annotated as lSTB Premature1-a and lSTB Premature 1-b. In addition, cluster 1 and 3 were annotated as lSTB Mature 1-a and lSTB Mature 1-b, accordingly. Late-pregnancy-specific STB nuclear subclusters have also been identified using snATAC-seq profiles for high gene activity scores of PTCHD4 (cluster 7) and DDX60 (cluster 8), respectively. However, a very small fraction of classified nascent state of STB nuclei (cluster 2) showed slightly elevated gene activity score of FLT1 on the UMAP plot of snATAC-seq data at late pregnancy, suggesting that a failure of FLT1 expression may be responsible for the paucity of FLT1 positive nuclei at late pregnancy. To confirm this, we scrutinized the aggregated snATAC-seq tracks for the indicated cluster with cluster-specific genes, and we found the chromatin was closed around the FLT1 locus at late pregnancy (Supplementary Fig. 8b).

Supplementary Note 19

Understanding how the turnover of STB nuclei is regulated at late pregnancy is crucial for gaining insight into normal pregnancies and labor. Next, we sought to connect the gene expression patterns and differentiation trajectories to the accessibility dynamics of the regulatory elements. Notably, when compared with data set at early pregnancy, we found a slightly poor agreement of gene activity score with gene expression (Supplementary Fig. 8d), due to the existence of residual RNA of FLT1 from early pregnancy and a bundle of residual RNA of FLT1-like genes. Consequently, we applied the GLUE algorithm [16], which places emphasis on peaks and gene regulatory proximity to the linear genome, instead of correlation between gene activity score and gene expression in the liger method (Fig. 5g and Supplementary Fig 9).

Supplementary Note 20

GO enrichment analysis revealed that extracellular matrix organization (ECM) was highly associated with nuclei in lSTB Mature 1-a and lSTB Mature 1-b subcluster (Fig. 5e, Supplementary Table 8), and LAMA3 was the target gene of the STAT6 regulatory network (Fig. 5i). LAMA3 is a laminin isoforms that exist in ECM and is involved in the invasive and metastatic abilities of some types of cancer [17,18]. ECM proteins were reported to be regulated by Glutamyl-prolyl-tRNA-synthetase via the TGFβ1/STAT signaling pathway [19]. The importance of extracellular matrix during healthy pregnancy including placenta invasion and labor is gradually being uncovered, and the findings we reported here may contribute to a better understanding of the regulatory mechanisms involved in normal pregnancies and disease. Further investigation is required to understand the regulatory mechanisms of the STAT signaling pathway in controlling the composition and function of STB extracellular matrix component at late pregnancy during normal labor and preterm birth.

Supplementary Note 21

It is worth noting that methods such as SCENIC+, FigR, chromVAR, and Homer, rely on established known TF motif databases to infer the comprehensive network and regulatory intricacies of TFs that control gene expression[20,21]. Alternative methods, such as the Homer *de novo* TF motif discovery algorithm and DeepMEL[22], do not rely on known TF motifs. However, these methods may not exhibit the same simplicity and efficiency as those based on established TF motifs. Therefore, in our study, we used existing TF databases, including hg38_screen_v10_clust and cisBP, for our data analysis. Key TFs such as STAT5A and CEBPB were consistently identified, regardless of those two the TF motif database used.

Supplementary Methods

Preprocess of raw sequencing data

Quality control. We took relatively strict criteria to filter for nuclei of high quality. For snRNA-seq data, we only kept nuclei with an UMI count of at least 3500, and the max UMI count was 50,000. Nuclei were then filtered with a expressed gene number range of 1,500 to 5,000, a mitochondrial transcripts percentage of less than 5%. For snATAC-seq, we first filtered with a range of logarithmic fragments count of 3.5 to 5 and a range of fraction of reads in promoter (FRiP) of 0.2 to 0.5. We filtered peaks against the black list regions (hg38). Our analysis revealed the high reproducibility between biological replicates. The mean gene expression value (or mean peak accessibility value) of the biological replicates was tested by the Pearson correlation with an average of r-value of 0.87 for snRNA-seq donors and 0.96 for snATAC-seq donors.

Doublet and low-quality nuclei filtering. For the snRNA-seq, we perform doublet filtering by python package Scrublet[23] with a cutoff of 0.5. For the snATAC-seq dataset, we filtered nuclei with very high sequence depth (logCPM >4.698, 95% quantile) to remove doublet with the following criteria. (1) detect and filter doublets with Scrublet (python package); (2)filter high depth; (3) filter dots that are too dispersed from the cluster centroid; (4) fix final cluster result by distance (> 90th percentile) from cluster centers with manual adjustment.

Splicing analysis with RNA velocity analysis

For the splicing analysis using velocity, we applied the python package velocyto[24] (v0.17) to calculate the ratio of spliced and un-spliced reads ratio from a processed bam file in each sample's cellranger-7.0 output directory. The results were saved separately as loom files and loaded by scVelo[25] (v0.2.5). We then integrated the spliced and un-spliced data into a pre-calculated anndata object (related to Fig. 1b) and visualized it with pie-chart, bar-plot and violin-plot with the 'proportions' and 'violin' functions.

Integration and gene regulatory network analysis in late pregnancy

Similar to the analysis steps in early pregnancy, We used R packages Seurat and SnapATAC coordinately to snRNA-seq and snATAC-seq data. General dimension reduction, nuclei clustering, and differentially expressed gene identification were conducted. Monocle2[26] was used for trajectory inference. For late pregnancy integration and TF-target regulatory analysis, since the existence of remnant gene expression transcripts (FLT1 and FLT1-like genes), which results in a slightly poor

agreement of gene activity score and gene expression, it is not as satisfied as the integration of two modalities (snATAC-seq and snRNA-seq) in the early gestation stage. We applied the GLUE algorithm[108], which was developed with the emphasis on peak and gene regulatory proximity relationship on the linear genome, instead of the liger method, to see the detailed structure of the trophoblast lineage agreements between two data modalities. Accordingly, we used a more straightforward TF-mining and TF-regulatory network analysis[27]. We utilized the R package FigR (v0.1.0)[27,28], to estimate the transcription factor (TF) regulatory network (GRN) during trophoblast lineage differentiation in late pregnancy. FigR employs a three-step approach starting with identifying the domain of regulatory regions (DORCs) by correlating chromatin accessibility peaks with the expression of nearby genes. Next, FigR extracts regulatory TF candidates from DORCs by considering both the TF motif enrichment score and the TF gene expression level. Finally, a two-layer regulatory network consisting of TFs and their targets is generated.

Identification of sex-biased differentially expressed genes

We utilized various strategies to identify sex-biased DEGs: 1) To identify sex-biased DEGs of the entire placenta tissue at each stage of pregnancy (early and late), we aggregated the raw UMI count matrix of each pregnancy stage. We calculated the sum of matrix by grouping the samples (columns), without consideration of cell types, using Matrix::rowSums (three female samples and three male samples). After the transition from single cell data to pseudo-bulk data, we applied the glmQLFTest function from the R package edgeR v3.28.1. We performed three replications for each gender to identify DEGs. The resulting DEGs were visualized using a volcano plot, with DEGs plotted as blue and red dots. The top 10 genes were labeled by adding short lines to them. 2) For the identification of sex-biased DEGs specific to the STB, we employed the strategy at the single cell level. We initially split the entire Seurat object (related to Fig. 1b) including only STB nuclei by stage. Then we applied the FindAllMarkers function (min.pct = 0.25, logfc.threshold = 0.25), considering sex annotations for each pregnancy stage. The results were visualized using a scatter plot, with sex-biased DEGs depicted as blue and red dots (Supplementary Fig. 12a and 12b). The top 10 genes were labeled by adding short lines to them. We further classified the resulting STB-specific sex-biased DEGs using the Ensembl v92 human gene biotype annotation.

Immunohistochemistry

Fresh tissues were collected and fixed in 4% paraformaldehyde (PFA) at 4°C overnight. After the dehydration with ascending series of ethanol (75%, 85%, 95%, 100%, and 100%) and clear process with xylene, the placental tissues were embedded into the

wax block and sectioned at a thickness of 5 μm. For the performance of the immune staining process, paraffin was routinely removed from paraffin sections by being washed in xylene, and the tissues were next hydrated in descending series of ethanol (100%, 100%, 100%, 95%, 85%, 75%). After washing with PBS three times, the sections were microwaved in 10 mM sodium citrate buffer (pH 6.0) for 15 min to retrieve the antigen. After cooling down to RT, immunohistochemistry staining was conducted according to the instruction of the immunohistochemistry staining kit (Cat#PV-9001, ZSGB-BIO). Briefly, the sections were treated with mouse monoclonal anti-KRT7 (Cat#ZM-0071, ZSGB-BIO). Positive signals were indicated by brown staining with diaminobenzidine, and the nuclei were stained blue with haematoxylin. The immunostained specimens were imaged using a microscope (Leica Aperio VESA8).

Immunofluorescence. Trophoblast organoids were fixed with 4% PFA for 20 min, permeabilized with 0.5% Triton X-100 for 20 min, and blocked with 3% BSA for 1 h at RT. The organoids were then incubated with the primary antibodies overnight at 4℃. The following primary antibodies were used: rabbit anti-CDH1 (Cat# 3195S Cell Signaling Technology, 1:200), mouse anti-hCG (Cat#ZM-0134, ZSGB-BIO, 1:200). Secondary antibodies were Alexa Fluor 488-(Cat#A21206, Invitrogen, 1:200) and Alexa Fluor 568 (Cat#A10037, Invitrogen, 1:200) -conjugated secondary antibodies. Nuclei were stained with DAPI (25 mg/ml). Images were collected under a Zeiss LSM 880 confocal laser scanning microscope and the image processing was performed with the ZEN software.

Enzyme-linked immunosorbent assay (ELISA). The experiment was conducted according to the instructions provided by Bluegene (Cat# E01C2095). In brief, the medium of trophoblast organoids was collected at day 6 and dispensed with Balance Solution to incubate with the standard wells for 1 hour at 37°C. The Substrate A and Substrate B Solution were added and incubated for 20 minutes at 37°C after washing. The reaction was terminated with Stop Solution and the Optical Density (O.D.) was determined at 450 nm using a microplate reader immediately.

Quantitative real-time PCR. Total RNA was isolated using TRIzol reagent (Invitrogen). cDNA was synthesized using HiScript® III All-in-one RT SuperMix Perfect for qPCR (Vazyme) and amplified with TB Green® Premix Ex Taq™ II (TaKaRa) on a Touch Thermal Cycler Real-Time PCR system (Roche, LightCycler480). GAPDH expression level was used as the internal normalization control. The primers for RNA quantification used in this study are listed in Supplementary Table 5.

Supplementary reference

1. Oliva, M. *et al.* The impact of sex on gene expression across human tissues. *Science* **369**(2020).
2. Zheng, D. *et al.* Sexual dimorphism in the incidence of human cancers. *BMC Cancer* **19**, 684 (2019).
3. Ngo, S.T., Steyn, F.J. & McCombe, P.A. Gender differences in autoimmune disease. *Front Neuroendocrinol* **35**, 347-69 (2014).
4. Lu, X. *et al.* Fine-Tuned and Cell-Cycle-Restricted Expression of Fusogenic Protein Syncytin-2 Maintains Functional Placental Syncytia. *Cell Rep* **21**, 1150-1159 (2017).
5. Welch, J.D. *et al.* Single-Cell Multi-omic Integration Compares and Contrasts Features of Brain Cell Identity. *Cell* **177**, 1873-1887 e17 (2019).
6. Zou, Z., Forbes, K., Harris, L.K. & Heazell, A.E.P. The potential role of the E SRRG pathway in placental dysfunction. *Reproduction* **161**, R45-R60 (2021).
7. Chung, J.H., Larsen, A.R., Chen, E. & Bunz, F. A PTCH1 homolog transcriptionally activated by p53 suppresses Hedgehog signaling. *J Biol Chem* **289**, 33020-31 (2014).
8. Hafner, A., Bulyk, M.L., Jambhekar, A. & Lahav, G. The multiple mechanisms that regulate p53 activity and cell fate. *Nat Rev Mol Cell Biol* **20**, 199-210 (2019).
9. Fogarty, N.M., Ferguson-Smith, A.C. & Burton, G.J. Syncytial knots (Tenney-Parker changes) in the human placenta: evidence of loss of transcriptional activity and oxidative damage. *Am J Pathol* **183**, 144-52 (2013).
10. Zhu, D. *et al.* BAI1 Suppresses Medulloblastoma Formation by Protecting p53 from Mdm2-Mediated Degradation. *Cancer Cell* **33**, 1004-1016 e5 (2018).
11. Cigerciogullari, E. *et al.* The determination of normal percentages of syncytiotrophoblastic knots in various regions of placenta: where to count the syncytial knots. *Turk Patoloji Derg* **31**, 1-8 (2015).
12. Singh, V.P., McKinney, S. & Gerton, J.L. Persistent DNA Damage and Senescence in the Placenta Impacts Developmental Outcomes of Embryos. *Dev Cell* **54**, 333-347 e7 (2020).
13. Kouwaki, T. *et al.* Extracellular Vesicles Including Exosomes Regulate Innate Immune Responses to Hepatitis B Virus Infection. *Front Immunol* **7**, 335 (2016).
14. Oshiumi, H. *et al.* DDX60 Is Involved in RIG-I-Dependent and Independent Antiviral Responses, and Its Function Is Attenuated by Virus-Induced EGFR Activation. *Cell Rep* **11**, 1193-207 (2015).
15. Tannetta, D., Collett, G., Vatish, M., Redman, C. & Sargent, I. Syncytiotrophoblast extracellular vesicles - Circulating biopsies reflecting placental health. *Placenta* **52**, 134-138 (2017).
16. Cao, Z.J. & Gao, G. Multi-omics single-cell data integration and regulatory inference with graph-linked embedding. *Nat Biotechnol* (2022).
17. Sathyanarayana, U.G. *et al.* Aberrant promoter methylation of laminin-5-encoding genes in prostate cancers and its relationship to clinicopathological features. *Clin Cancer Res* **9**, 6395-400 (2003).
18. Virolle, T. *et al.* Binding of USF to a non-canonical E-box following stress results in a cell-specific derepression of the lama3 gene. *Nucleic Acids Res* **30**, 1789-98 (2002).
19. Peng, D., Fu, M., Wang, M., Wei, Y. & Wei, X. Targeting TGF-beta signal transduction for fibrosis and cancer therapy. *Mol Cancer* **21**, 104 (2022).

20. Badia, I.M.P. *et al.* Gene regulatory network inference in the era of single-cell multi-omics. *Nat Rev Genet* (2023).
21. Costa, I.G. Dissecting gene regulation with multimodal sequencing. *Nat Methods* (2023).
22. Janssens, J. *et al.* Decoding gene regulation in the fly brain. *Nature* **601**, 630-636 (2022).
23. Wolock, S.L., Lopez, R. & Klein, A.M. Scrublet: Computational Identification of Cell Doublets in Single-Cell Transcriptomic Data. *Cell Syst* **8**, 281-291 e9 (2019).
24. La Manno, G. *et al.* RNA velocity of single cells. *Nature* **560**, 494-498 (2018).
25. Bergen, V., Lange, M., Peidli, S., Wolf, F.A. & Theis, F.J. Generalizing RNA velocity to transient cell states through dynamical modeling. *Nat Biotechnol* **38**, 1408-1414 (2020).
26. Qiu, X. *et al.* Reversed graph embedding resolves complex single-cell trajectories. *Nat Methods* **14**, 979-982 (2017).
27. Ma, S. *et al.* Chromatin Potential Identified by Shared Single-Cell Profiling of RNA and Chromatin. *Cell* **183**, 1103-1116 e20 (2020).
28. Kartha, V.K. *et al.* Functional inference of gene regulation using single-cell multi-omics. *Cell Genom* **2**(2022).

Supplementary Figure Source Data

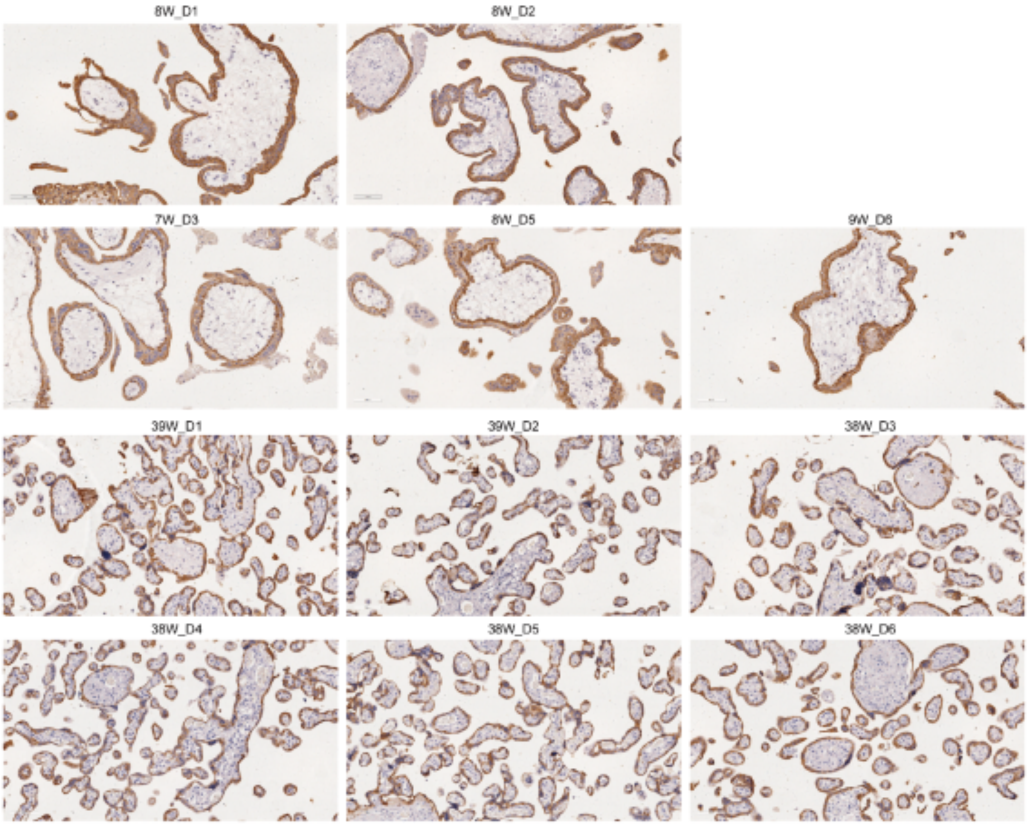

Supplementary Fig. 1a source data. Immuno-histochemistry staining of KRT7 shows the morphology of placentas used for sequencing.

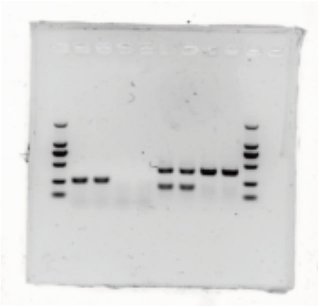

Supplementary Fig. 1c source data. Uncropped scans of the gel for PCR of two sex-linked genes before sequencing.

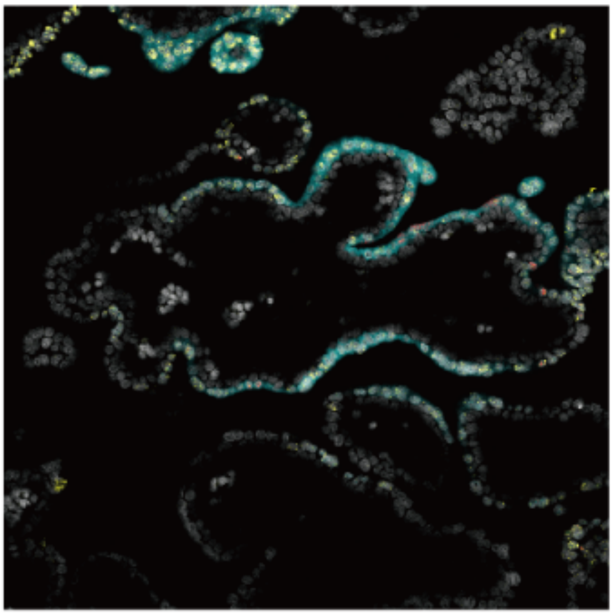

Supplementary Fig. 4h source data. smFISH staining of indicated marker genes (SH3TC2, LEP and PSG8) on human placenta in early pregnancy.

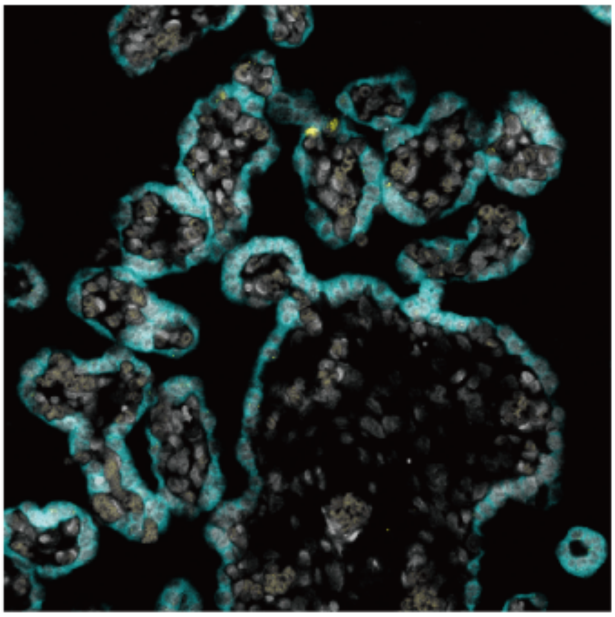

Supplementary Fig. 9h source data. smFISH staining of indicated marker genes (SH3TC2, LEP and PSG8) on human placenta in late pregnancy.
