# Supplementary material for: Single-nucleus multi-omic profiling of human placental syncytiotrophoblasts identifies cellular trajectories during pregnancy
Source: Nat Genet. 2024 Jan 24;56(2):294–305. doi: 10.1038/s41588-023-01647-w (PMC10864176; doi:10.1038/s41588-023-01647-w)
Supplement: Supplementary file 2 — Reporting Summary [file 41588_2023_1647_MOESM2_ESM.pdf]

nature portfolio

Corresponding author(s): Hongmei Wang

Last updated by author(s): Oct 13, 2023

Reporting Summary

Nature Portfolio wishes to improve the reproducibility of the work that we publish. This form provides structure for consistency and transparency in reporting. For further information on Nature Portfolio policies, see our Editorial Policies and the Editorial Policy Checklist.

Statistics

For all statistical analyses, confirm that the following items are present in the figure legend, table legend, main text, or Methods section.

n/a	Confirmed	
☐	☒	The exact sample size (*n*) for each experimental group/condition, given as a discrete number and unit of measurement
☐	☒	A statement on whether measurements were taken from distinct samples or whether the same sample was measured repeatedly
☐	☒	The statistical test(s) used AND whether they are one- or two-sided<br>*Only common tests should be described solely by name; describe more complex techniques in the Methods section.*
☒	☐	A description of all covariates tested
☐	☒	A description of any assumptions or corrections, such as tests of normality and adjustment for multiple comparisons
☐	☒	A full description of the statistical parameters including central tendency (e.g. means) or other basic estimates (e.g. regression coefficient) AND variation (e.g. standard deviation) or associated estimates of uncertainty (e.g. confidence intervals)
☐	☒	For null hypothesis testing, the test statistic (e.g. *F*, *t*, *r*) with confidence intervals, effect sizes, degrees of freedom and *P* value noted<br>*Give P values as exact values whenever suitable.*
☒	☐	For Bayesian analysis, information on the choice of priors and Markov chain Monte Carlo settings
☒	☐	For hierarchical and complex designs, identification of the appropriate level for tests and full reporting of outcomes
☐	☒	Estimates of effect sizes (e.g. Cohen's *d*, Pearson's *r*), indicating how they were calculated

Our web collection on statistics for biologists contains articles on many of the points above.

Software and code

Policy information about availability of computer code

Data collection	No software was used.
Data analysis	snRNA-seq raw data process: Cellranger v3.0.0 with gene annotation ensembl version 93 and Cellranger v7.0.1 with gene annotation gencode.v32 (https://support.10xgenomics.com/single-cell-gene-expression/software/pipelines/latest/what-is-cell-ranger)<br>snATAC-seq raw data process: Cellranger-atac v1.1.0 and Cellranger-atac v1.2.0 (https://support.10xgenomics.com/single-cell-atac/software/pipelines/latest/what-is-cell-ranger-atac)<br>Batch effect removal: Harmony v1.0 (https://portals.broadinstitute.org/harmony/index.html)<br>Standard snRNA-seq upstream analysis: Seurat v3.2.3 (https://satijalab.org/seurat/) and scanpy v1.8.2 (https://github.com/scverse/scanpy)<br>Standard snATAC-seq upstream analysis: SnapATAC v1.0.0 (https://github.com/r3fang/SnapATAC) and SnapATAC v2.2.0 (https://github.com/kaizhang/SnapATAC2)<br>Alternative snATAC-seq upstream analysis: Signac v1.1.0 (https://github.com/timoast/signac)<br>Doublet filtering: scrublet v0.2.3 (https://github.com/swolock/scrublet) and DoubletFinder v2.0.3 (https://github.com/chris-mcginnis-ucsf/DoubletFinder)<br>snRNA-seq trajectory inference: monocle 2.14 (http://cole-trapnell-lab.github.io/monocle-release/docs/)<br>snATAC-seq trajectory inference: functions in Granja et al 2019 scripts (https://github.com/GreenleafLab/ MPAL-Single-Cell-2019)<br>Differentially expressed gene identification between pregnancy stages: Seurat v3.2.3 (see above), diffxpy v0.7.4 (https://github.com/theislab/diffxpy), codes in Zemmour et al 2018 (https://static-content.springer.com/esm/art%3A10.1038%2Fs41590-018-0051-0/MediaObjects/41590_2018_51_MOESM8 _ESM.zip)<br>TF motif deviation score: chromVAR v1.8.0 (https://github.com/GreenleafLab/chromVAR)<br>TF motif database: JASPAR 2020 (https://jaspar.genereg.net/)<br>Gene activity score: bedops v2.4.39 (https://bedops.readthedocs.io/)

Gene Ontology enrichment: ClusterProfiler v3.18.1 (https://guangchuangyu.github.io/software/ clusterProfiler/). Optional GO enrichment: DAVID 2023 (https://david.ncifcrf.gov/)
Nuclei pairing: functions in Granja et al 2019 scripts (https://github.com/GreenleafLab/MPAL-Single-Cell-2019) and ArchR v1.0.1 (https://www.archrproject.com/)
Integration: liger v0.5.0 (https://github.com/welch-lab/liger)
Integration when gene chromatin openness slightly disagrees with gene expression: GLUE v0.2.3 (https://github.com/gao-lab/GLUE)
TF-motif enrichment in DAR: i-cisTarget online version (https://gbiomed.kuleuven.be/apps/lcb/i-cisTarget/) and pycistarget v1.0.2 (https://github.com/aertslab/pycistarget)
eGRN network construction in the early pregnancy by custom R codes of re-interpreting of method in Janssens et al 2022, TF regulatory network construction in the late pregnancy used figR v0.1.0 (https://github.com/buenrostrolab/FigR)
Regulatory network construction: Cytoscape v3.9.1 (https://cytoscape.org/)
splicing/unsplicing reads count: velocyto v0.17.17 (https://velocyto.org/)
splicing/unsplicing ratio calculation: scVelo v0.2.5 (https://scvelo.readthedocs.io/en/stable/)
sex-biased gene expression: edgeR v3.28.1
Platform version: R version 3.6.3 and 4.1.3, Python version: 3.8.5
Main custom code used in this study is available on GitHub (https://github.com/wangmeijiao/Human_placenta_multi-omics_snRNA-seq_snATAC-seq).
Fluorescence intensity quantification of single-molecule fluorescence in site hybridization (smFISH) with RNAScope: ZEN 2012 （blue edition, https://www.zeiss.com/microscopy/int/products/microscope-software/zen-lite.html）
Statistic plotting (for small data size): GraphPad Prism 9 (https://www.graphpad.com/updates/prism-900-release-notes)

For manuscripts utilizing custom algorithms or software that are central to the research but not yet described in published literature, software must be made available to editors and reviewers. We strongly encourage code deposition in a community repository (e.g. GitHub). See the Nature Portfolio guidelines for submitting code & software for further information.

Data

Policy information about availability of data

All manuscripts must include a data availability statement. This statement should provide the following information, where applicable:

- Accession codes, unique identifiers, or web links for publicly available datasets
- A description of any restrictions on data availability
- For clinical datasets or third party data, please ensure that the statement adheres to our policy

Sequencing data generated for this study have been deposited in the Gene Expression Omnibus (GEO) with the accession number GSE247038. There are no restrictions on data availability or use. The primary data (gene-cell expression matrix and peak-cell accessibility matrix) and the processed upstream results (R rds file) were deposited on figshare repository, with share links:

https://figshare.com/s/61db5dc9030e6363267e for 8w_atac,
https://figshare.com/s/ccec028e5a3ec5b20106 for 8w_rna; https://figshare.com/s/05fdcc5d0c6b46786047 for term_atac, https://figshare.com/s/5a435d5ea48b3d51b40b for term_rna.
https://figshare.com/s/40bc9295d533d163b738 for cell line.

The rds/h5ad files were saved with dimension reduction and nuclei clustering results, and can well matched the UMAP embeddings in Figure 2 and Figure 5. 8w: the early pregnancy, term: the late pregnancy, We also uploaded raw result tables for construction of eGRN networks (https://figshare.com/s/a3ec1630f75de248698b).

All figshare processed files are accessible to public.

We used the human genome version GRCh38 to carry out all the analysis in this study. The genome database can be downloaded from the 10X website with file name "refdata-gex-GRCh38-2020-A.tgz".

Human research participants

Policy information about studies involving human research participants and Sex and Gender in Research.

Reporting on sex and gender	A total of 18 female pregnant donors were involved in the project.
Population characteristics	The early pregnancy placentas (6 gestation weeks to 9 gestation weeks) were obtained from healthy female pregnant donors undergoing elective pregnancy termination individually. The late pregnancy placentas (from 38 gestation weeks to 39 gestation weeks) were only acquired from healthy female pregnant donors who chose to deliver by cesarean section to avoid cellular contamination from the birth canal.
Recruitment	A total of 18 patients were involved in the project. The placental specimens used in this study of snRNA-seq and snATAC-seq were collected from 6 early pregnant women (6-9 weeks of pregnancy) and 6 late pregnant women (38-39 weeks of pregnancy). Additional 3 healthy placental tissues from the 3 early pregnant women (7-8 weeks of pregnancy) and 3 placental tissues from the 3 late pregnant women (38 weeks of pregnancy) were used for experimental validation (RNAscope). All samples were randomly selected except for sex balance consideration. There is no sex-biased effect on the single nuclei data analysis, including dimension reduction, cell type annotation and differentially expressed gene identification.
Ethics oversight	This research was approved by the Ethics Committee of the Institute of Zoology, the Chinese Academy of Sciences, and the Ethics Committee of the Peking University Third Hospital (research license number: (2019) JLS No. (242-01)). All patients

participating in the study were healthy pregnant women without any exclusion. Every patient involved in the project has signed the informed consent. All the patients in our research project shared one ethical approval license number.

Note that full information on the approval of the study protocol must also be provided in the manuscript.

Field-specific reporting

Please select the one below that is the best fit for your research. If you are not sure, read the appropriate sections before making your selection.

☒ Life sciences ☐ Behavioural & social sciences ☐ Ecological, evolutionary & environmental sciences

For a reference copy of the document with all sections, see nature.com/documents/nr-reporting-summary-flat.pdf

Life sciences study design

All studies must disclose on these points even when the disclosure is negative.

Sample size	No statistical methods were used to predetermine sample size. 6 healthy placentas of 6 pregnant human donors at early pregnancy and 6 healthy placentas of 6 pregnant human donors at late pregnancy were collected, and analyzed. 50,580 single nuclei for snRNA-seq and 50,693 single nuclei for snATAC-seq were analyzed in total for human placental to capture the full nuclear repertoire of the human placental, and to reveal the dynamic features and heterogeneity of STB nuclei at early- and late pregnancy. For STB nuclear subcluster verification in situ, at least 3 replicates were performed. Figure s1a was performed in 5 healthy first-trimester placentas and 6 healthy third-trimester placentas. Figure 2g was performed in 6 healthy first-trimester placentas. Figure 3e and Figure s3h were performed in 3 healthy first-trimester placentas. Figure 5f was performed in 6 healthy third-trimester placentas. Figure s9h was performed in 3 healthy third-trimester placentas.<br>To ensure that this sample size expansion could meet the standard of the community so that we have enough data to derive accurate statistics. We further performed snRNA-seq and snATAC-seq analyses on STB nuclei obtained during early and late pregnancy. Following downstream analysis, we counted the number of STB nuclei and the percentage of STB nuclear subtypes in our dataset by adding one sample at a time (from 1 to 6 samples) to determine if there is a correlation between the potential diversity nuclei subtype and sample size. As expected, the total number of STB nuclei, as well as the numbers of Mature 1 and Mature 2 STB nuclei increased steadily as more placentas were included. However, the percentage of these two STB subtypes among the total number of STB nuclei remained relatively stable . To investigate the variability in expression profiles among STB nuclear subtypes, we identify differentially expressed genes (DEGs) cumulatively across all STB nuclei and visualize their occurrence in the heatmap. The top25 DEGs of eSTB Mature 1 and eSTB Mature 2 were consistent with our previous findings. Hence we believe that six samples (with sex balance consideration) are sufficient to decipher STB heterogeneity and carry out downstream analysis.
Data exclusions	For snRNA-seq data, we only kept nuclei with an UMI count of at least 3500 instead of 500 in some studies, and the max UMI count 50,000. Nuclei then were filtered with a expressed gene number range of 1500 to 5000, a mitochondrial transcripts percentage of less than 5%. For snATAC-seq, we first filtered for a range of logarithmic fragments count of 3.5 to 5 and a range of fraction of reads in promoter (FRiP) of 0.2 to 0.5. We filtered peaks against the black list regions (hg38).<br>For both data modalities, nuclei that distribute too far away (>90th percentile) from the centroids of each clusters were filtered out (totally less than 100 nuclei were filtered out after this step).
Replication	A total of 18 donors participated in the project. Specifically, we obtained placenta tissues from 6 healthy donors at early pregnancy (6-9 weeks of gestation) and 6 healthy donors at late pregnancy (38-39 weeks of gestation) for sequencing. Among these 12 placentas for sequencing, we ensured an equal representation of male and female placentas at each stage to eliminate potential sex-related biases. Besides, we used additional 3 healthy placentas from early pregnancy and 3 from late pregnancy for experimental verifications. To be more precise, we conducted snRNA-seq and snATAC-seq on 6 healthy placental tissues from early pregnant women and 6 healthy tissues from late pregnant women, among which 5 out of 6 early placentas and all 6 late placentas underwent immunohistochemistry to ensure typical villous architecture, and 3 out of 6 early placentas and 3 out of 6 late placentas underwent RNAscope. Additionally, we used 3 healthy placentas at early pregnancy and 3 healthy placentas at late pregnancy for RNAscope. The placentas from early pregnancy were obtained from donors undergoing elective pregnancy termination individually, while the placentas from late pregnancy were obtained from donors who opted for cesarean section to avoid cellular contamination from the birth canal.
Randomization	The early pregnancy placentas were obtained from healthy female pregnant donors undergoing elective pregnancy termination individually. The late pregnancy placentas were acquired from healthy female pregnant donors who chose to deliver by cesarean section to avoid cellular contamination from the birth canal. Placentas meeting these criteria were randomized for single-nulceus library preparation and experimental validation.
Blinding	All placental samples were processed individually for nuclear isolation, sequencing, and experimental validation. Investigators were blinded during sample collection, data collection and analysis. Placenta collection and data analysis were performed by different researchers at different locations.

Reporting for specific materials, systems and methods

We require information from authors about some types of materials, experimental systems and methods used in many studies. Here, indicate whether each material, system or method listed is relevant to your study. If you are not sure if a list item applies to your research, read the appropriate section before selecting a response.

Materials & experimental systems

n/a	Involved in the study
☐	☒ Antibodies
☐	☒ Eukaryotic cell lines
☒	☐ Palaeontology and archaeology
☒	☐ Animals and other organisms
☒	☐ Clinical data
☒	☐ Dual use research of concern

Methods

n/a	Involved in the study
☐	☒ ChIP-seq
☒	☐ Flow cytometry
☒	☐ MRI-based neuroimaging

Antibodies

Antibodies used

anti-KRT7(ZM-0071; ZSGB-BIO);
http://www.zsbio.com/product/ZM-0071

anti-CDH1 (3195T; Cell Signaling Technology);
https://www.cellsignal.com/products/primary-antibodies/e-cadherin-24e10-rabbit-mab/3195?site-search-type=Products&N=4294956287&Ntt=3195t&fromPage=plp&_requestid=1419924

anti-hCG (ZM-0134; ZSGB-BIO);
http://www.zsbio.com/product/ZM-0134

anti-CEBPB (23431-1-AP; Proteintech);
https://www.ptglab.com/products/CEBPB-Antibody-23431-1-AP.htm

anti-FOSL2(351814; USBiological);
https://www.usbio.net/antibodies/351814/FOSL2-FRA2-Fosrelated-Antigen-2

anti-IgG(ab172730; Abcam);
https://www.abcam.cn/products/primary-antibodies/rabbit-igg-monoclonal-epr25a-isotype-control-ab172730.html

Validation

Validations are available for all antibodies from the manufacturer. Please refer to references contained in the provided links.

Eukaryotic cell lines

Policy information about cell lines and Sex and Gender in Research

Cell line source(s)

CTB-derived hTSCs (hTSCs-CT30) were derived in Okae lab.
Blastocyst-derived hTSCs (hTSCs-BL) were derived in Wang lab.
hESCs-RUES2 derived in were Brivanlou lab.
HEK293T cells were preserved in our lab.

Authentication

The hTSC cell line was authenticated by morphology, karyotyping and immunostaining with TP63/GATA3/TEAD4.

Mycoplasma contamination

The cell line is negative for mycoplasma contamination.

Commonly misidentified lines
(See ICLAC register)

No commonly misidentified cell lines were used.

ChIP-seq

Data deposition

☒ Confirm that both raw and final processed data have been deposited in a public database such as GEO.

☒ Confirm that you have deposited or provided access to graph files (e.g. BED files) for the called peaks.

Data access links
May remain private before publication.

https://www.ncbi.nlm.nih.gov/geo/query/acc.cgi?acc=GSE247035

Files in database submission

GSE247035_RAW.tar

Genome browser session
(e.g. UCSC)

no longer applicable.

Methodology

Replicates

We sequenced two biological replications for antibody CEBPB and control for two cell lines (hTSC and hTSC-derived STB). There is

Replicates	good correlation among two biological replications.
Sequencing depth	We sequenced 10.7M,24.2M, 26M, 34M raw reads for two replications of two cell lines with 150bp pair-end library construction strategy. After Bowtie2 mapping, totally 10307456,22596908, 24584898, 30388418 read pairs were mapped to the human genome (GRCh38). Of these 8191223, 17371860, 17923579, 20904903 read pairs were unique mapped reads.
Antibodies	anti-CEBPB (23431-1-AP; Proteintech)
Peak calling parameters	macs2 callpeak -t STB-CEBPB-1.chipseq.bam -c STB-IgG.chipseq.bam --outdir out_STB_CEBPB1_vs_STB_IgG_q0.01 --tempdir . -g hs -f BAMPE -n STB_CEBPB1_vs_STB_IgG --keep-dup all --qval 0.01 -B --SPMR
Data quality	We used fastqc toolkits to check fastq quality. The trim_glore program was used to filter for high quality reads if necessary. After MACS2 peak calling step, totally 79339, 128033, 45165, 31440 peaks were obtained with FDR >= 0.01 and >5 fold thresholds.
Software	We applied bowtie2 (v2.4.2) with default parameters for raw fastq reads mapping to human (hg38) reference genome and used MACS2 to call peaks for CUT&Tag and ChIP-seq datasets. We used the 'reduce' function from the R package GenomicRanges for merge of peaks of each replications.
