# Supplementary figures and images for: Single-nucleus multi-omic profiling of human placental syncytiotrophoblasts identifies cellular trajectories during pregnancy
Source: Nat Genet. 2024 Jan 24;56(2):294–305. doi: 10.1038/s41588-023-01647-w (PMC10864176; doi:10.1038/s41588-023-01647-w)

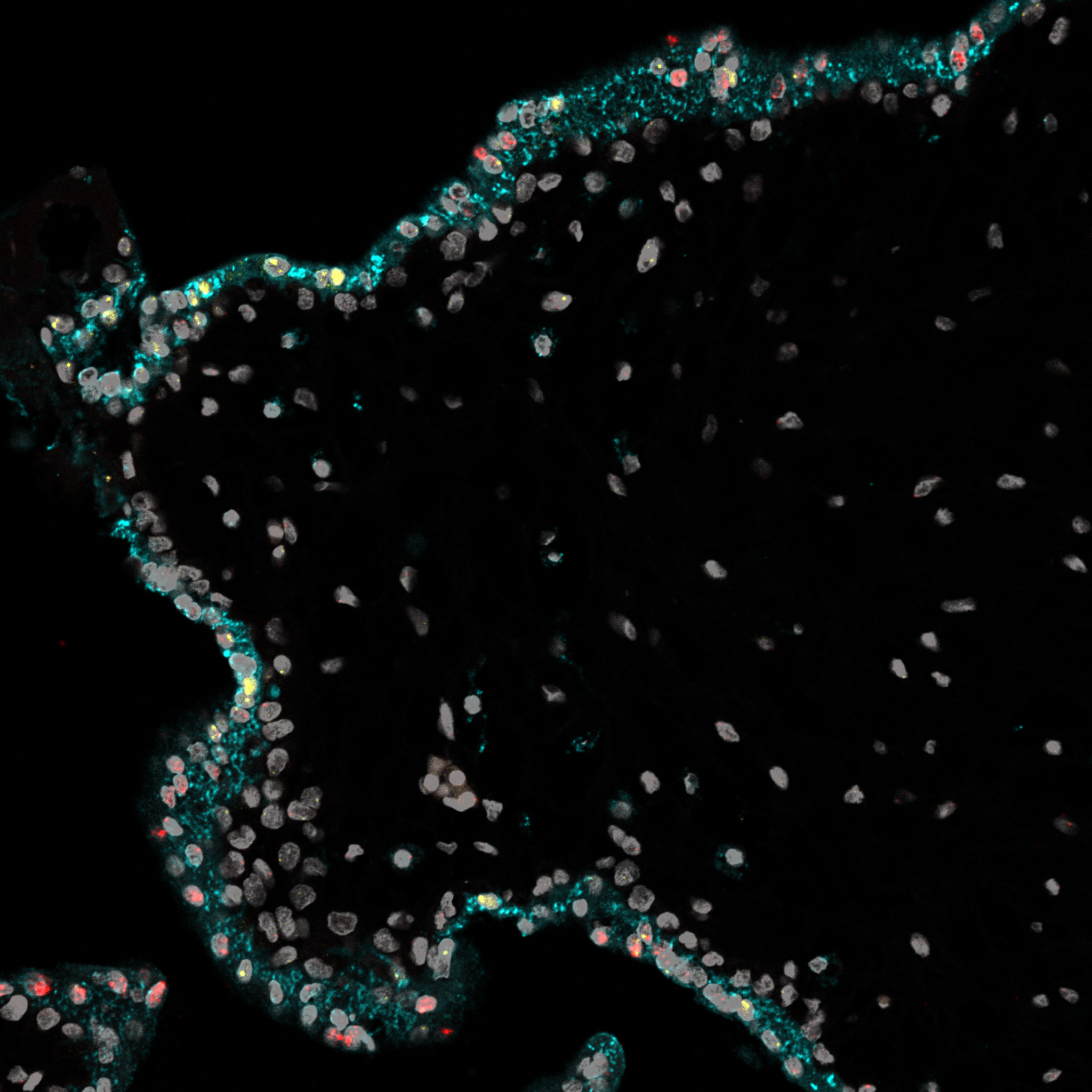

Supplement: Supplementary file 6 — Normalized gene expression matrix for comparison of in vivo and in vitro STB population transcriptional signature. [file 41588_2023_1647_MOESM6_ESM.tif]

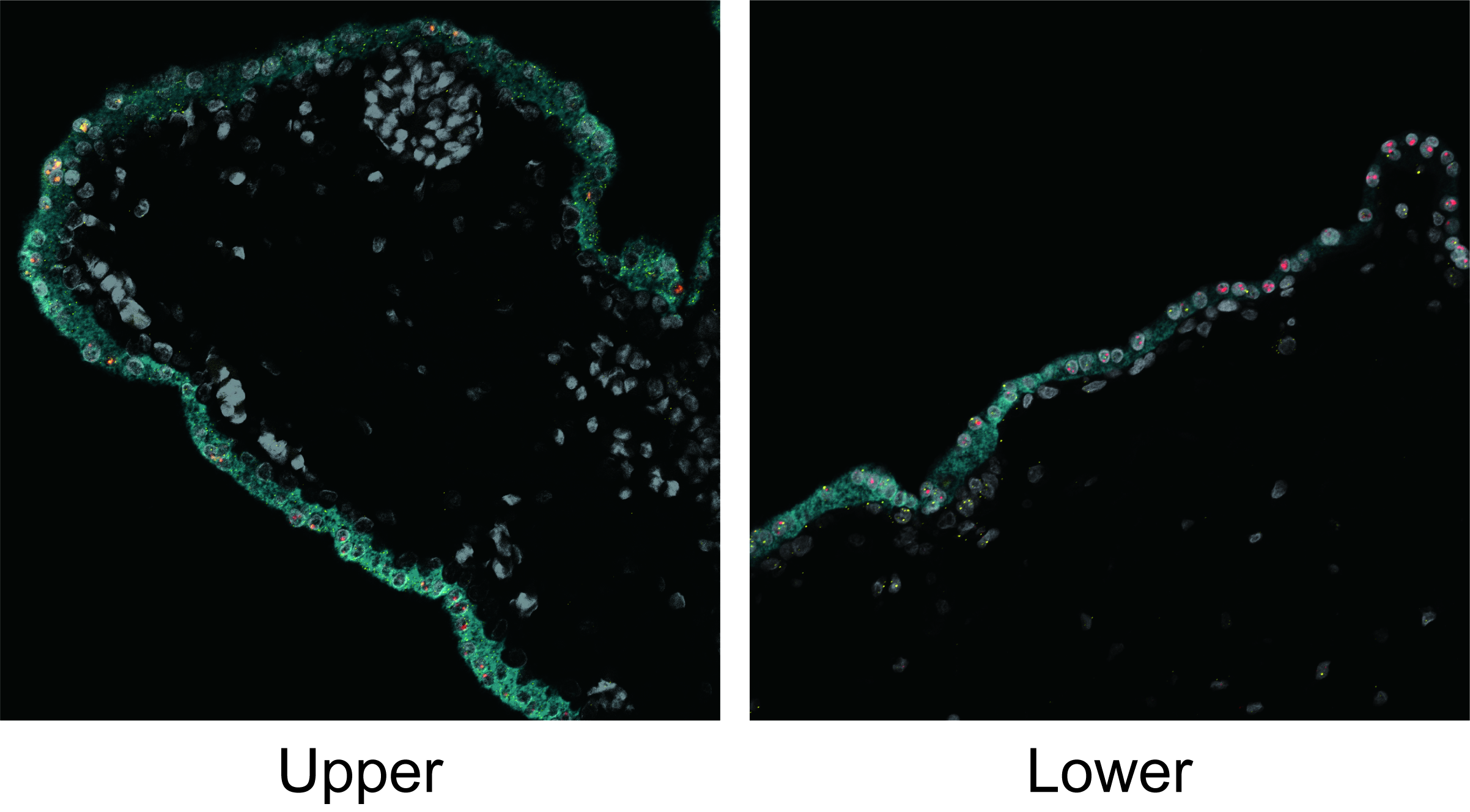

Supplement: Supplementary file 7 — smFISH staining of TFs (STAT5A and FOSL2) and representative genes (PAPPA and FLT1) of eSTB mature 1 and eSTB mature 2. [file 41588_2023_1647_MOESM7_ESM.tif]

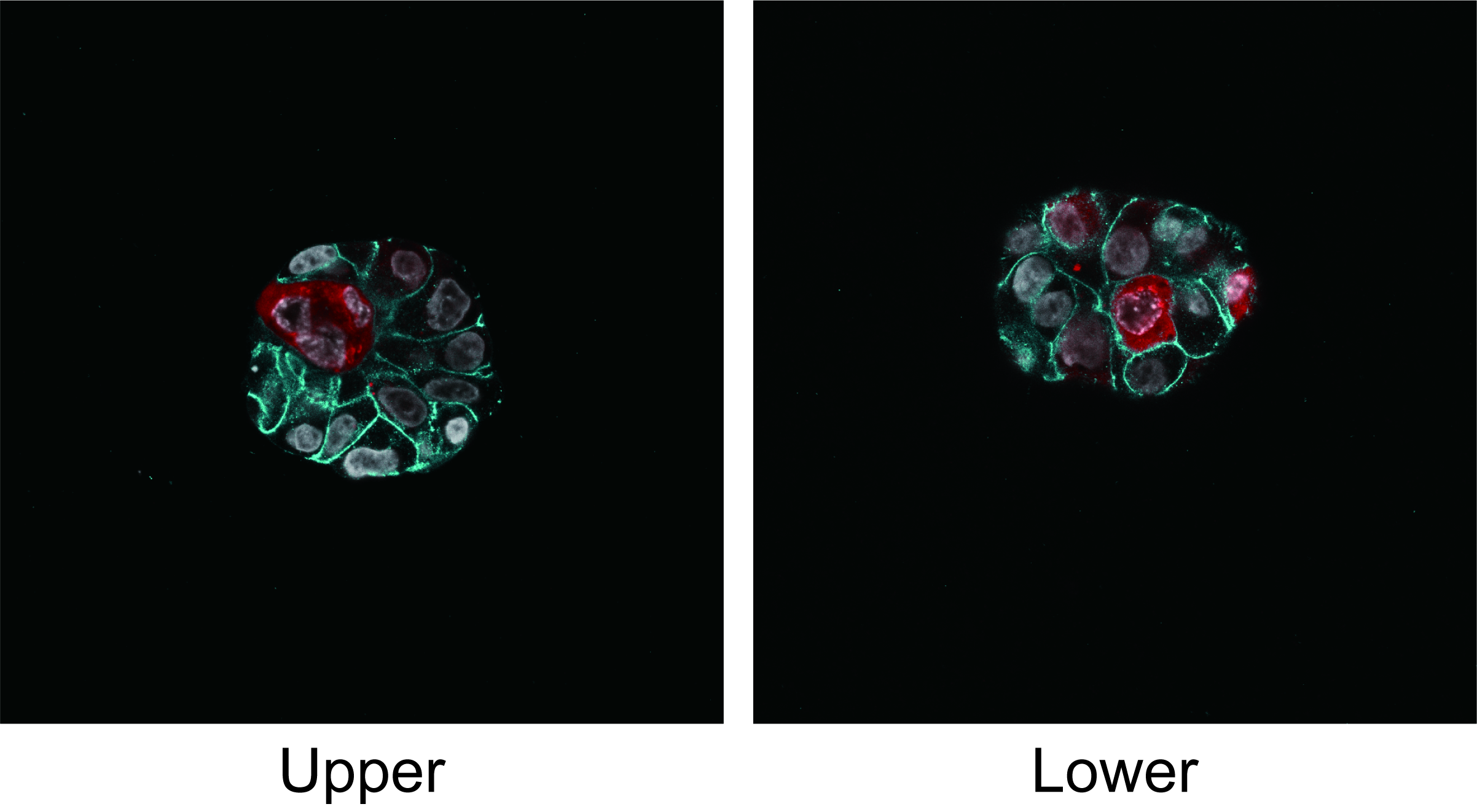

Supplement: Supplementary file 8 — Immunofluorescence images of trophoblast markers (CDH1 and hCG) in TO derived from hTSCs-BL and MITF-overexpressing hTSCs-BL. [file 41588_2023_1647_MOESM8_ESM.tif]

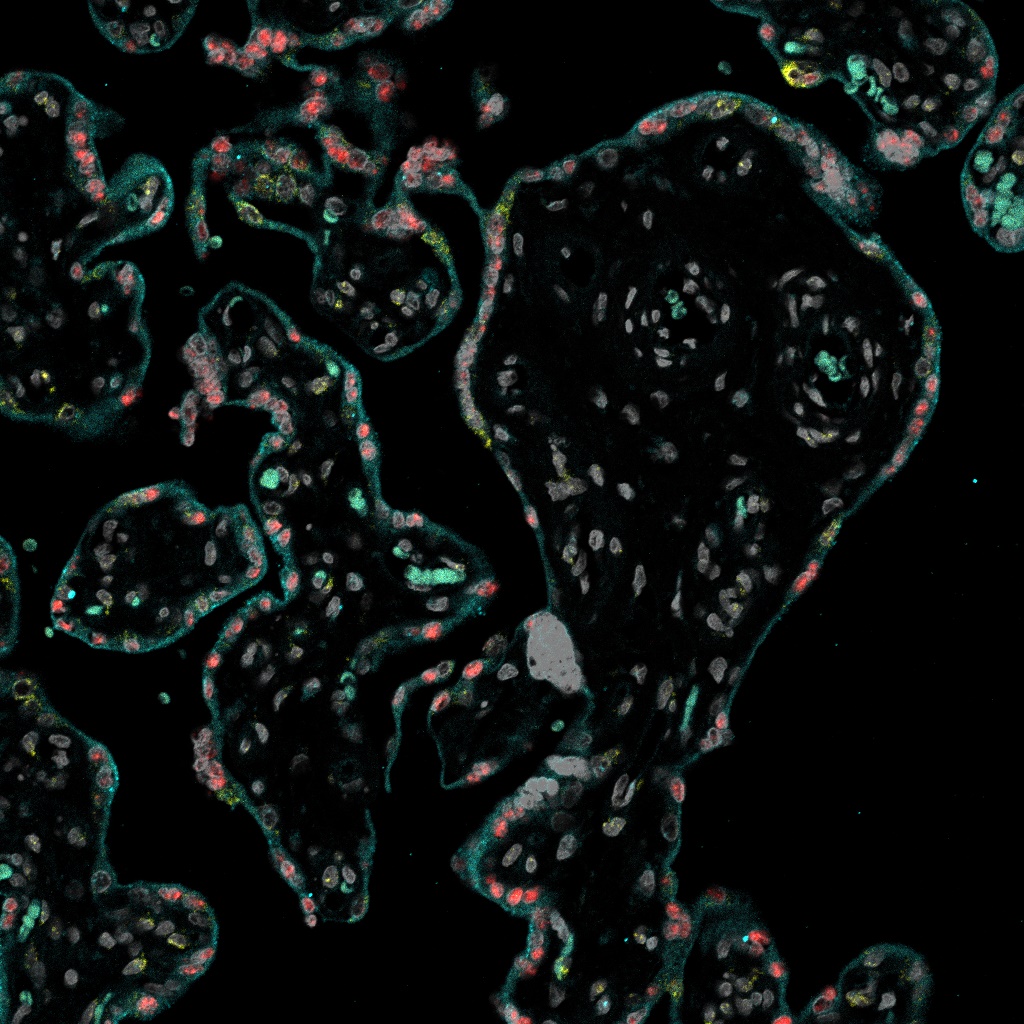

Supplement: Supplementary file 9 — smFISH staining of indicated marker genes (PAPPA, FLT1 and hCG) characterizes lSTB mature 1 and lSTB mature 2 in late pregnancy. [file 41588_2023_1647_MOESM9_ESM.jpg]
